# Efferocytosis‐Driven Polyamine Metabolism in Macrophages Enhances Cancer Stem Cell Enrichment after Chemotherapy in Ovarian Cancer

**DOI:** 10.1002/advs.202512508

**Published:** 2025-11-21

**Authors:** Wenhan Li, Feiquan Ying, Xinkai Pang, Qiulei Wu, Guoqing Li, Lin Huang, Jiayu Xin, Xiaoli Liu, Pan Liu, Xiaohan Xu, Shuran Tan, Yumei Gao, Tong Liu, Si Sun, Xiaoman Wang, Yiping Wen, Liqiong Cai, Shi Du, Yuan Zhang, Jing Cai

**Affiliations:** ^1^ Department of Obstetrics and Gynecology Union Hospital Tongji Medical College Huazhong University of Science and Technology Wuhan 430000 China; ^2^ Department of Obstetrics and Gynecology The Second Affiliated Hospital of Chongqing Medical University Chongqing 400010 China; ^3^ Department of Obstetrics and Gynecology The First Affiliated Hospital Sun Yat‐sen University Guangzhou 510080 China; ^4^ Department of Gynecology Qingdao Municipal Hospital Qingdao 266000 China; ^5^ Department of Gynecology Xiangya Hospital Central South University Changsha 410008 China

**Keywords:** cancer stemness, chemotherapy, efferocytosis, macrophage, ovarian cancer

## Abstract

Chemotherapy‐induced enrichment of cancer stem cells (CSCs) is a key mechanism underlying acquired chemoresistance and recurrence of epithelial ovarian cancer (OC). Although chemotherapy may enrich CSCs through selection or by inducing dedifferentiation, the dynamic changes in the tumor niche and their impact on CSCs during chemotherapy remain unclear. In this study, single‐cell sequencing and multiplex immunohistochemical analysis are used to define microenvironmental changes, and a post‐chemotherapy increase in efferocytotic macrophages that phagocytosed chemotherapy‐induced apoptotic tumor cells is identified. Efferocytotic macrophages are associated with poor prognosis and CSCs in OC. Their conditioned medium facilitates OC stemness in vitro. Meanwhile, targeting efferocytosis suppresses CSC enrichment, chemoresistance, and regrowth in vivo. Mechanistically, it is demonstrated that enhanced expression of ODC1 driven by efferocytosis increases polyamine flux, particularly putrescine, by integrating metabolomics and transcriptomics. The increase in putrescine content leads to the *SPP1* and OPN overexpression in macrophages, conferring cancer stemness to OC cells through the OPN‐CD44 axis. Treatment with an ODC1 selector inhibitor mitigates CSC enrichment, sensitizes tumors to cisplatin, and restricts tumor regrowth. Together, the study shows that efferocytosis and associated polyamine metabolic reprogramming support the chemotherapy‐induced enrichment of CSCs, providing new targets for addressing chemoresistance and recurrence of OC.

## Graphical abstract

Chemotherapy‐induced efferocytosis drives ovarian cancer stem cell enrichment. By engulfing apoptotic cancer cells, macrophages upregulate ODC1 and produce putrescine, which elevates osteopontin (OPN) expression. Secreted OPN then activates the CD44 receptor on cancer cells, promoting stemness and chemoresistance. This study uncovers a macrophage–polyamine–OPN axis as a crucial pathway in post‐chemotherapy relapse.

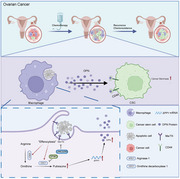

## 1 Introduction

The treatment of epithelial ovarian cancer (EOC) is particularly challenging, mainly due to its frequent diagnosis at advanced stages, where surgery alone is unlikely to achieve a cure, and chemotherapy is essential. Despite advances in systemic therapy, especially integrating bevacizumab and poly ADP‐ribose polymerase inhibitors into traditional platinum‐based chemotherapy, the 5‐year survival rate of EOC patients has not been significantly improved.^[^ ^1^, ^2^ ^]^ Although nearly 80% of EOC patients respond to primary platinum‐based chemotherapy, most eventually experience tumor recurrence and gradually develop resistance to platinum agents, and such platinum‐resistant recurrences are largely incurable.^[^ ^2^ ^]^ Cancer stem cells (CSCs) have been recognized as the driver of cancer recurrence and chemoresistance,^[^ ^3^ ^]^ owing to their self‐renewal,^[^ ^4^ ^]^ immune‐escape,^[^ ^5^ ^]^ and chemo‐resistant properties.^[^ ^6^, ^7^ ^]^ Our previous study demonstrated that ovarian cancer stem cells were enriched in both human and murine models after chemotherapy,^[^ ^8^ ^]^ suggesting that the chemotherapy kills most tumor cells and leads to clinical remission; meanwhile, it may leave CSCs behind for future recurrence. Therefore, elucidating the mechanisms underlying CSCs enrichment during chemotherapy and developing targeted strategies to counteract this process are crucial for improving patients’ survival.

Recent studies have demonstrated that CSC enrichment post‐cytotoxic therapy results from selective survival pressure and therapy‐induced tumor cell‐intrinsic reprogramming.^[^ ^3^ ^]^ However, the role of the tumor microenvironment in this process remains poorly explored. Cytotoxic therapy‐induced tissue damage activates tissue repair responses and recruits neutrophils, macrophages, fibroblasts, lymphocytes, and endothelial cells. Among them, macrophages participate in all the three stages of tissue repair, including inflammation, resolution, and regeneration, highlighting their critical role due to their heterogeneity.^[^ ^9^ ^]^ During the inflammation phase, macrophages migrate to the damaged sites to remove dead cells.^[^ ^9^ ^]^ In the resolution phase, they adopt an anti‐inflammatory phenotype to attenuate inflammation.^[^ ^10^, ^11^, ^12^ ^]^ In the regeneration and remodeling phases, they activate stem cells, promote angiogenesis, and remodel the extracellular matrix to restore tissue homeostasis.^[^ ^9^, ^13^ ^]^ However, whether their stem cell activation function is hijacked by cancer cells to permit cancer cells to survive under cytotoxic therapy and renew themselves to induce recurrence remains underexplored.

Here, by integrating patient‐derived xenograft (PDX) models, single‐cell RNA sequencing (scRNA‐seq), bulk RNA sequencing, and multiplex immunohistochemical (mIHC) analysis, we identified that chemotherapy drives the expansion of a monocyte‐derived tissue repair macrophage cluster associated with poor prognosis in ovarian cancer patients. Macrophages typically phagocytose apoptotic cells in tissue damage, triggering metabolic reprogramming toward a tissue repair phenotype.^[^ ^10^, ^14^ ^]^ The process by which phagocytic cells remove dead cells via phagocytosis is termed efferocytosis.^[^ ^15^ ^]^ Following enrichment analysis, mIHC, and in vitro efferocytosis assays, the tissue repair macrophage cluster was identified as an efferocytotic macrophage cluster. Although macrophages have been reported to suppress immune responses via efferocytosis during cytotoxic therapy, which is utilized by cancer cells for progression,^[^ ^16^, ^17^ ^]^ the role of efferocytosis in the maintenance of cancer stemness requires further investigation. Consequently, we investigated the role of efferocytosis and its associated metabolic processes in supporting cancer stemness of ovarian cancer cells using in vitro experiments, metabolomics, RNA sequencing, and mouse models, including subcutaneous, intraperitoneal, and orthotopic xenografts, as well as PDX models. Our study revealed that targeting efferocytosis and its associated metabolic processes can inhibit post‐chemotherapy CSCs enrichment, thereby revealing promising therapeutic targets to address recurrence and chemoresistance in EOC.

## 2 Results

### 2.1 Macro_c2 Population Expands after Chemotherapy and is Associated with Poor Prognosis in OC

**Figure 1:**
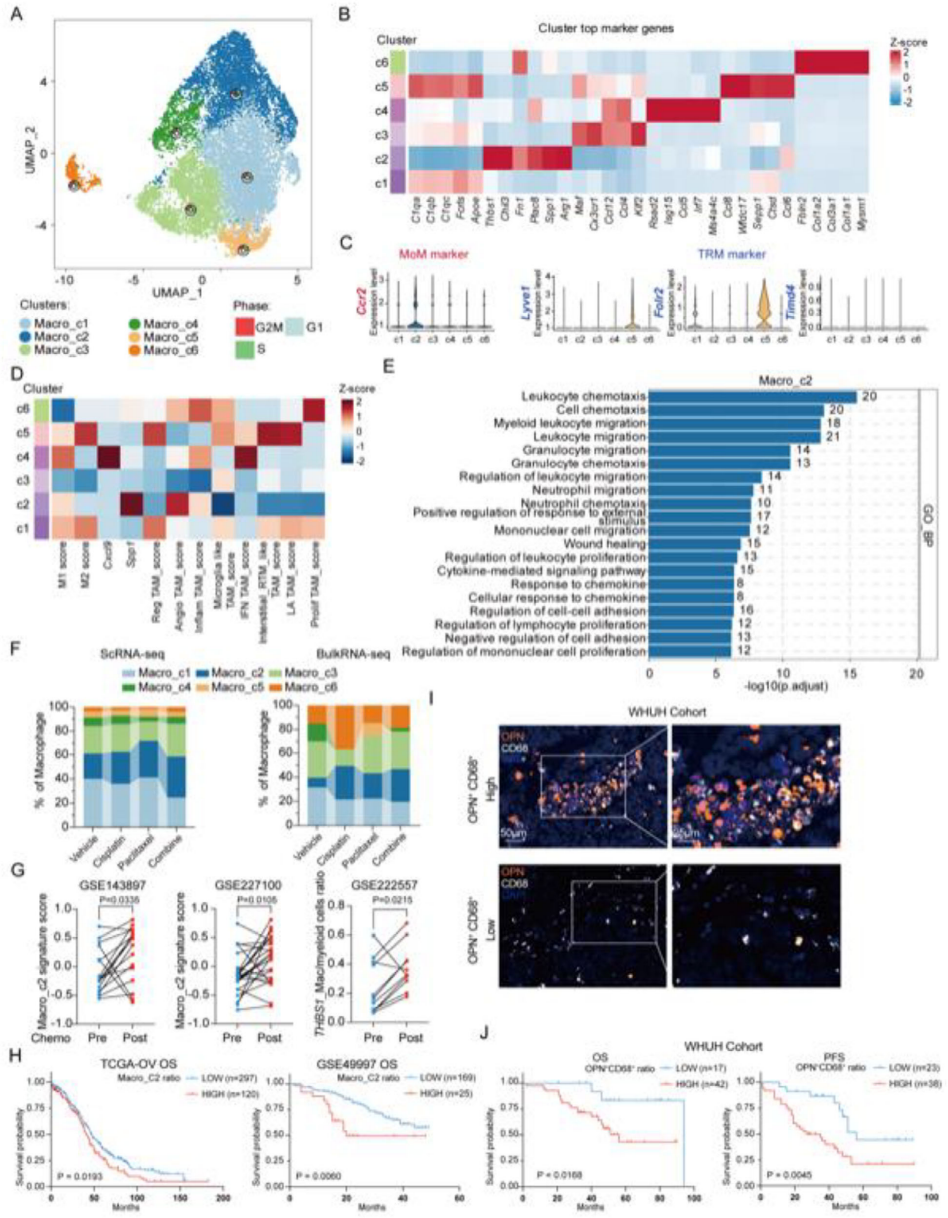
Single‐cell sequencing revealed evolving macrophage diversity in the tumor microenvironment (TME) during chemotherapy. A) Uniform Manifold Approximation and Projection (UMAP) visualization of macrophages from patient‐derived xenograft (PDX) single‐cell RNA sequencing (scRNA‐seq). Points are colored by macrophage cluster identity. Cycling plots display the cell‐cycle phase distribution across macrophage clusters. B) Heatmap showing the top differentially expressed genes (DEGs) expression across different macrophage clusters. C) Violin plots showing the expression of monocyte‐derived macrophage (MoM) markers (*Ccr2*) and tissue‐resident macrophage (TRM) markers (*Lyve1*, *Flt2*, *Timd4*) across macrophage clusters. D) Heatmap showing M1 score, M2 score, *Cxcl9* expression, *Spp1* expression, Reg (regulatory) TAM score, Angio (angiogenesis) TAM score, Inflam (inflammation) TAM score, Microglia like TAM score, IFN TAM score, interstitial RTM (resident tissue macrophage) like TAM score, LA (lipid associated) TAM score, and Prolif (proliferation) TAM score across different macrophage clusters. All scores were calculated by Gene Set Variation Analysis (GSVA). E) GO:BP (Gene Ontology: biological process) enrichment analysis in Macro_C2 based on its DEGs. F) Left, stacked bar chart showing the ratios of macrophage clusters between scRNA‐seq of vehicle, cisplatin, paclitaxel, and combined chemotherapy groups. Right, stacked bar chart showing the ratios of macrophage clusters between bulk RNA‐sequencing of vehicle, cisplatin, paclitaxel, and combined chemotherapy groups. The ratios were calculated by deconvolution analysis through cibersorX. G) Left and center: Paired line plots comparing Macro‐c2 signature scores in pre‐chemotherapy and post‐chemotherapy groups using GSE143897 and GSE227100 datasets. Right: Paired line plots comparing the ratio of THBS1^+^ macrophage/myeloid cells in pre‐chemotherapy and post‐chemotherapy groups using GSE222557. Statistical significance was assessed using paired t‐tests. H) Kaplan–Meier analysis of overall survival (OS) for ovarian cancer patients in TCGA‐OV and GSE49997 between macro_c2 high ratio group and macro_c2 low ratio group. Statistical significance was tested using the log‐rank (Mantel‐Cox) test. I) Representative mIHC (multiplex immunohistochemistry) images of a high OPN^+^ CD68^+^ ratio patient sample and a low OPN^+^ CD68^+^ ratio patient sample. OPN, orange; CD68, white. K) Kaplan–Meier analysis of OS and progression‐free survival (PFS) for ovarian cancer patients in the WHUH cohort between the OPN^+^ CD68^+^ high ratio group and the OPN^+^ CD68^+^ low ratio group. Statistical significance was tested using the log‐rank (Mantel‐Cox) test. For all survival analyses, patients were divided into low‐ratio and high‐ratio groups based on the optimal cut‐off value calculated using the R package survminer (version 0.5.0).

To investigate the alterations in macrophages in patients with ovarian cancers undergoing chemotherapy, PDX models of a high‐grade serous ovarian cancer were established and treated with vehicle, cisplatin, paclitaxel, or a combination of cisplatin and paclitaxel, followed by scRNA‐seq of the xenografts (Figure S1A,B, Supporting Information). After quality control, 46 094 single‐cell transcriptomes were used for subsequent analysis. Cells could be grouped into five main cell types (nine clusters): fibroblasts, macrophages, dendritic cells, neutrophils, and endothelial cells, which were annotated using known markers (Figure S1C,D, Supporting Information). The composition and gene expression of the macrophages were analyzed. Re‐clustering of 14,490 macrophages revealed six diverse cell subpopulations (Macro_c1‐c6) (**Figure** **1**A), and their marker genes were identified (Figure 1B). Given that both tissue‐resident macrophages (TRMs) and monocyte‐derived macrophages (MoMs) can be present in the tumor microenvironment (TME), we identified the lineage of the six macrophage clusters using their markers. We found that Macro_c2 highly expressed the MoM marker *Ccr2*, while Macro_c5 highly expressed the TRM markers *Lyve1* and *Flor2*. Macro_c1 also showed relatively high *Flor2* signals. (Figure 1C).

Advanced single‐cell sequencing has revealed diverse macrophage phenotypes and *CXCL9*/*SPP1* polarity in the TME extending beyond the classical M1/M2 polarization.^[^ ^18^, ^19^ ^]^ To analyze these phenotypes, Gene Set Variation Analysis (GSVA) was performed on six macrophage subpopulations based on their molecular signatures. Among the M1/M2‐like phenotypes, Macro_c4 exhibited the highest M1 score, while Macro_c5 displayed the highest M2 score. Although Macro_c2 did not show a high M2 score, it was enriched for M2‐associated genes such as *Spp1*, *Arg1*, and *Chil3*, indicating a M2‐like phenotype. In terms of *CXCL9*/*SPP1* polarity, Macro_c4 demonstrated high *Cxcl9* expression with low *Spp1* expression, whereas Macro_c2 showed the opposite profile, with high *Spp1* and low *Cxcl9* expression. Additionally, Macro_c1 exhibited high scores for regulatory tumor‐associated macrophage (regulatory TAM) and interstitial RTM‐like TAM, while Macro_c2 presented the highest angiogenesis (Angio) TAM score, suggesting a proangiogenic phenotype. Macro_c3, on the other hand, was associated with an elevated microglia TAM score, and Macro_c4 exhibited high scores for inflammation (Inflam) and interferon (IFN) TAM. Notably, Macro_c5 showed the highest regulatory TAM, lipid‐associated (LA) TAM, and interstitial RTM‐like TAM scores, whereas Macro_c6 demonstrated a prominent proliferation (Prolif) TAM score (Figure 1D).

Gene Ontology Biological Process (GO BP) enrichment analysis further characterized the macrophage populations (Figure 1E; Figure S2A–E, Supporting Information). Macro_c1 was associated with lipid storage (Figure S2A, Supporting Information), while Macro_c2 exhibited enriched pathways related to myeloid leukocyte migration, positive regulation of responses to external stimuli, wound healing, and regulation of lymphocyte proliferation, consistent with a tissue repair phenotype (Figure 1E). Macro_c4 showed significant enrichment in antigen processing and presentation (Figure S2C, Supporting Information), whereas Macro_c6 was enriched for extracellular matrix and collagen fibril organization (Figure S2E, Supporting Information). Given the crucial role of macrophages in immune checkpoint regulation,^[^ ^20^ ^]^ we next analyzed the expression of immune checkpoint genes across macrophage subsets. Our analysis revealed distinct expression patterns, with Macro_c2 showing significant upregulation of inhibitory immune checkpoint genes, while Macro_c4 expressed stimulatory immune checkpoint genes (Figure S2F, Supporting Information).

To assess macrophage dynamics during chemotherapy, we analyzed subcluster abundance. Macro_c2 abundance increased across all chemotherapy regimens compared to vehicle controls, as confirmed by scRNA‐seq and bulk RNA‐seq deconvolution (Figure 1F). To examine whether the changes in the Macro C2 in clinical samples before and after chemotherapy were consistent with those observed in our experimental models, we analyzed the public datasets and found Macro_c2 signature was also upregulated in post‐chemotherapy samples compared to pre‐chemotherapy samples in the GSE143897 and GSE227100 datasets (Figure 1G). In the public scRNA‐seq GSE222557 dataset, the ratio of macrophages with high *THBS1* expression, a top key Macro_c2 marker, also increased after chemotherapy (Figure 1G; Figure S2G, Supporting Information). These results suggest that the Macro_C2 population may increase following chemotherapy. To explore the clinical relevance of these macrophage populations, we estimated their abundance in patient samples from the TCGA‐OV and GSE49997 cohorts using CIBERSORTx. High Macro_c2 abundance correlated with poor overall survival (OS), while Macro_c4 abundance was associated with favorable OS, consistent with the *CXCL9*:*SPP1* polarity^[^ ^19^ ^]^ (Figure 1H). Further analysis in our ovarian cancer cohort using Macro_c2 marker OPN (encoded by *SPP1*) and macrophage marker CD68 revealed that patients with a low OPN^+^ CD68^+^ ratio exhibited significantly prolonged OS (P = 0.0168) and progression‐free survival (PFS) (P = 0.0045) (Figure 1I,J).

In summary, our data identify diverse macrophage populations in the chemotherapy‐associated TME. Notably, Macro_c2, potentially monocyte‐derived and expanded post‐chemotherapy, exhibits immunosuppressive and tissue repair phenotypes, correlating with poor survival outcomes in ovarian cancer patients.

### 2.2 Macro_c2 Population Exhibits Phenotype of Efferocytotic Macrophages

**Figure 2:**
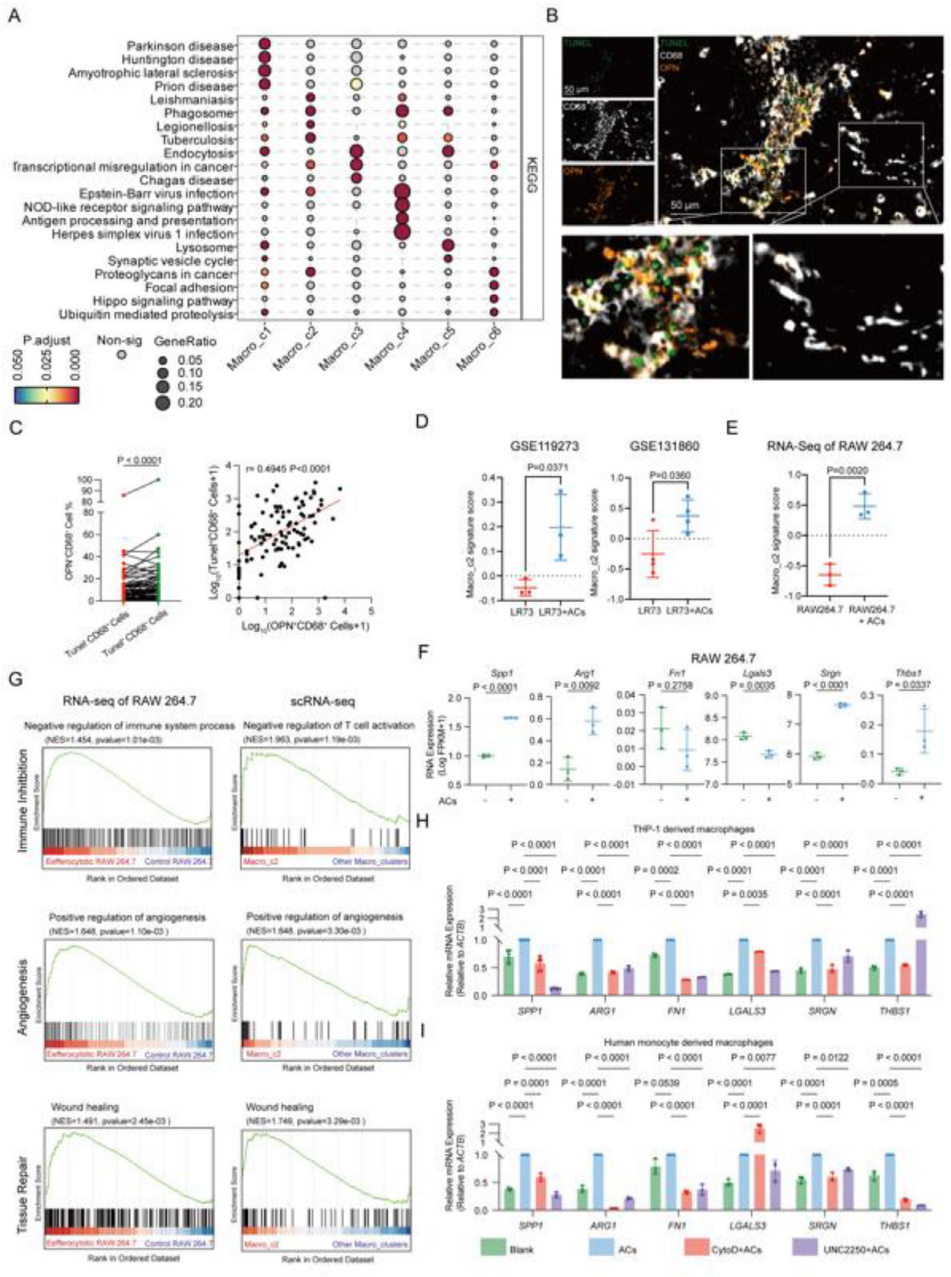
Identification of Macro_C2 as efferocytotic macrophages. A) Dot plots showing the results of KEGG (Kyoto Encyclopedia of Genes and Genomes) enrichments across the macrophage clusters. B) Representative mIHC (multiplex immunohistochemistry) images of ovarian cancer tisssues. Tunel, green; OPN, orange; CD68, white. C) Left, paired line plot showing OPN^+^ CD68^+^ cell ratio in Tunel^+^ CD68^+^cells or Tunel^−^ CD68^+^cells, Statistical significance was tested using the paired t‐test. Right, Correlation between Tunel^+^ CD68^+^ cells and OPN^+^ CD68^+^ cells (n = 104). Statistical significance was tested using Spearman correlation analysis. D) Scatter plots showing the Macro_C2 signature scores in LR73 cells versus LR73 cells co‐cultured with apoptotic cells (ACs) from the GSE119273 (n = 3) and GSE131860 (n = 4) dataset. Statistical significance was assessed using Student's t‐test. E) Scatter plot comparing the Macro_C2 signature scores in RAW264.7 cells (n = 3) versus RAW264.7 cells co‐cultured with ACs (n = 3). Statistical significance was assessed using Student's t‐test. F) Scatter plots showing the expressions of *Spp1*, *Arg1*, *Fn1*, *Lgals3*, *Srgn*, and *Thbs1* in RAW264.7 cells and RAW264.7 cells co‐cultured with ACs. Statistical significance was tested using Student's t‐test. G) GSEA (Gene Set Enrichment Analysis) results highlighting pathways associated with immune inhibition, angiogenesis, and tissue repair in RAW264.7 cells co‐cultured with ACs and Macro_C2. H) qRT‐PCR (Quantitative real‐time PCR) analysis of relative mRNA expression in THP‐1‐derived macrophages with vehicle, vehicle+ACs, ACs+UNC2250, and ACs+CytoD. Before adding ACs, macrophages were incubated with vehicle, CytoD, and UNC2250 for 2 h. qRT‐PCR analysis of *Spp1* (n = 6 independent experiments) and other genes (n = 3 independent experiments). Statistical significance was tested using one‐way ANOVA and Dunnett's multiple comparisons test. I) qRT‐PCR analysis of relative mRNA expression in human monocyte‐derived macrophages with vehicle, vehicle+ACs, ACs+UNC2250, and ACs+CytoD. Before adding ACs, macrophages were incubated with vehicle, CytoD, and UNC2250 for 2 h (n = 3 independent experiments). Statistical significance was tested using one‐way ANOVA and Dunnett's multiple comparisons test. All data are presented as Mean ± SD.

Efferocytosis, the process by which macrophages clear dying cells, inhibits inflammation, accelerates wound healing, and regulates tissue repair, is a key function of macrophages in the TME.^[^ ^9^, ^15^ ^]^ We hypothesized that chemotherapy‐induced cell death leads to tissue repair and immunosuppressive phenotypes of macrophages through efferocytosis. Notably, both efferocytotic macrophages and Macro_c2 cells exhibit similar tissue repair and immunosuppressive functions. Besides, Macro_c2 and efferocytotic macrophages shared functional similarities: enriched phagosomes but impaired antigen processing and presentation, as evidenced by KEGG (Kyoto Encyclopedia of Genes and Genomes) enrichment, while the two terms were both significantly enriched in Macro_c4 (**Figure** **2**A). In colon cancers, *SPP1* ^+^ TAMs, *ISG5* ^+^ TAM, and *CXCL9* ^+^ TAM have been identified as phagocytic macrophages, and *SPP1* ^+^ TAMs lack antigen processing and presentation compared to the other two phagocytizing TAMs,^[^ ^21^ ^]^ which is consistent with our enrichment analysis in Macro_c2 (*Spp1* high expression) and Macro_c4 (*Isg5* and *Cxcl9* high expression). To validate our hypothesis in ovarian cancer, we observed that OPN^+^ CD68^+^ cells (identified as Macro_c2 cells) were located in TUNEL‐positive areas (Figure 2B). The ratio of OPN^+^ CD68^+^ cells in TUNEL^+^ CD68^+^ cells was higher than that in TUNEL^−^ CD68^+^ cells. The number of OPN^+^ CD68^+^ cells was also positively correlated with that of TUNEL^+^ CD68^+^ cells in our ovarian cancer patient cohort, suggesting that Macro_c2 is associated with efferocytotic macrophages (Figure 2C; Table S5, Supporting Information).

Consistent with our hypothesis, a higher Macro_c2 signature score was observed in LR73 cells (hamster phagocytes) co‐cultured with apoptotic Jurkat cells compared to LR73 cells alone (Figure 2D; Figure S4D, Supporting Information). To further validate this, we performed bulk RNA sequencing on RAW264.7 cells engulfing apoptotic human ovarian cancer A2780 cells (Figure S4A–C, Supporting Information). Similarly, a higher Macro_c2 signature score was found in RAW264.7 cells co‐cultured with apoptotic A2780 cells compared to RAW264.7 cells alone (Figure 2E; Figure S4D, Supporting Information). Moreover, RAW264.7 cells incubated with apoptotic cells (ACs) expressed higher levels of Macro_c2 marker genes (*Spp1*, *Arg1*, *Srgn*, *Thbs1*) than control RAW264.7 cells (Figure 2F). These cells also exhibited phenotypes similar to Macro_c2, including pro‐angiogenesis, immunosuppression, and tissue repair, as determined by GSEA analysis (Figure 2G).

To confirm that these changes were induced by efferocytosis, we inhibited the process using MerTK inhibitor UNC2250 and actin polymerization inhibitor Cytochalasin D (CytoD). As expected, both inhibitors reduced efferocytosis in macrophages co‐cultured with apoptotic ovarian cancer cells (Figure S4E,F, Supporting Information). Treatment with apoptotic cells led to increased expression of *SPP1*, *FN1*, *ARG1*, *LGALS3*, *SRGN*, and *THBS1*, whereas co‐treatment with the inhibitors reduced these expressions (Figure 2H,I).

In conclusion, these findings demonstrate that Macro_c2 and efferocytotic macrophages exhibit similar phenotypes, gene expression profiles, and locations within the TME, thereby classifying Macro_c2 as an efferocytotic macrophage.

### 2.3 Inhibition of Efferocytosis Constrains Tumor Chemoresistance and CSCs Enrichment In Vivo

**Figure 3:**
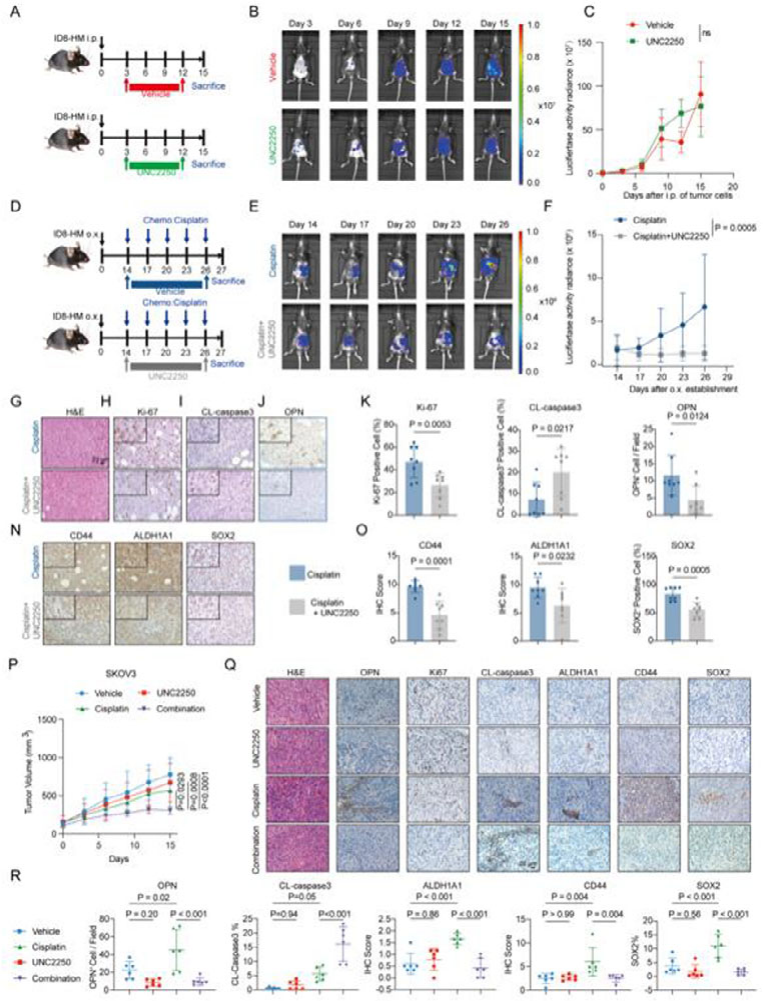
Inhibiting efferocytosis halts tumor regrowth and chemoresistance in vivo. A) Treatment of ID8‐HM tumor‐bearing mice using UN2250 or vehicle. ID8‐HM cells were intraperitoneally injected into C57BL/6J mice. Treatment was initiated at the 3rd day after the injection and sustained for a duration of 9 days. B) Representative images of the intraperitoneal tumor xenografts treated with UN2250 or vehicle. Tumor burden was assessed by performing in vivo imaging using a live‐imaging system on a schedule of every 3 days. C) Tumor growth curves for mice treated with UNC2550 (n = 4) and vehicle (n = 4). Statistical significance was tested using two‐way ANOVA and Šídák's multiple comparisons test. ns, not significant. D) Treatment of ID8‐HM tumor‐bearing mice using cisplatin combined with UN2250 or vehicle. Tissue blocks of subcutaneous xenografts derived from ID8‐HM cells were transplanted into the ovarian bursae of C57BL/6J mice to establish orthotopic xenografts. Treatment of UNC2250 or vehicle was initiated on the 14th day after the orthotopic xenografts establishment and sustained for a duration of 12 days. Treatment of cisplatin was initiated on the 14th day after the orthotopic xenografts establishment, then every 3 days. E) Representative images of the orthotopic tumor xenografts treated with cisplatin combined with UN2250 or vehicle. Tumor burden was assessed by performing in vivo imaging using a live ‐ imaging system on a schedule of every 3 days. F) Tumor growth curves for mice treated with cisplatin combined with UN2250 (n = 8) or vehicle (n = 8). Statistical significance was tested using two‐way ANOVA and Šídák's multiple comparisons test. G) Representative H&E (Hematoxylin and eosin) staining images of in the orthotopic tumor xenografts treated with cisplatin combined with UN2250 or vehicle. H) Representative IHC (Immunohistochemistry) images of Ki67 staining. The images in the upper left corner with double the magnification. I) Representative IHC images of cleaved‐caspase3 staining. The images in the upper left corner with double the magnification. J) Representative IHC images of OPN staining. The images in the upper left corner with double the magnification. K) Bar graph showing the percentage of cleaved‐caspase3^+^cell, the percentages of Ki67^+^ cells, and the number of OPN^+^ cells in the orthotopic tumor xenografts treated with cisplatin combined with UN2250 (n = 8) or vehicle (n = 8). Statistical significance was tested using the Student's t‐tests. N) Representative IHC images of CD44, ALDH1A1, and SOX2 staining in the orthotopic tumor xenografts treated with cisplatin combined with UN2250 or vehicle. The images in the left upper corner with twice magnification. O) Bar graphs showing the CD44, ALDH1A1 levels, and percentage of SOX2^+^cell in the orthotopic tumor xenografts treated with cisplatin combined with UN2250 (n = 8) or vehicle (n = 8). Statistical significance was tested using the Student's t‐tests. P) Tumor of SKOV3 cells growth curves for mice treated with UNC2550 (n = 6), vehicle (n = 6), cisplatin (n = 6), and combination (n = 6). Statistical significance was tested using two‐way ANOVA and Tukey's multiple comparisons test. Q) Representative H&E staining images of P, and representative IHC images of ΟPN, Ki67, cleaved‐caspase3, CD44, ALDH1A1, and SOX2 staining οf P. R) Bar graphs showing the percentage of cleaved‐caspase3^+^cells, the number of OPN^+^ cells, CD44, ALDH1A1 levels, and percentage of SOX2^+^cells in P. Statistical significance was tested using οne‐way ANOVA and Šídák's multiple comparisons test. All data are presented as Mean ± SD.

Given the association of efferocytotic macrophages with poor outcomes, we tested whether blocking efferocytosis could affect ovarian cancer progression. Using UNC2250, we treated ovarian cancer models (ID8‐HM cells and SKOV3 cells) with or without chemotherapy. In intraperitoneal tumors of ID8‐HM cells, UNC2250 alone did not affect tumor growth (**Figure** **3**A–C). In mice receiving cisplatin and paclitaxel with or without UNC2250 (Figure S5A, Supporting Information), both chemotherapy regimens inhibited tumor growth and decreased tumor burden, as is commonly observed in ovarian cancer; however, the tumor burden in the chemotherapy‐only group was much higher than that in the chemotherapy plus UNC2250 group on day 6 after treatment. Tumors in the chemotherapy‐only group exhibited rapid regrowth by day 6, whereas no regrowth was observed in the combination group. Moreover, UNC2250 treatment reduced the number of OPN⁺ cells and the levels of CD44 in omental metastatic nodules (Figure S5B–G, Supporting Information). In orthotopic tumors of ID8‐HM cells, tumor growth in the cisplatin plus UNC2250 group was significantly inhibited (Figure 3H–F). IHC confirmed that blocking efferocytosis during chemotherapy decreased proliferation (lower Ki67^+^ cells ratio) and increased apoptosis (higher cleaved‐caspase3^+^ cells ratio) compared to cisplatin alone (Figure 3G–K). Importantly, UNC2250 also lowered the number of OPN^+^ cells (Figure 3J). We hypothesized that chemotherapy resistance and regrowth are driven in part by CSC enrichment. Indeed, UNC2250 treatment during cisplatin prevented the rise of CSC markers (CD44, ALDH1A1, SOX2) in ID8‐HM tumors (Figure 3N,O). In subcutaneous tumors of SKOV3 cells, consistent effects on tumor burden and CSC marker expression were observed, similar to those in the ID8‐HM models (Figure 3P–R; Figure S4H,I, Supporting Information). Taken together, pharmacologic inhibition of efferocytosis during chemotherapy halted tumor regrowth, enhanced chemosensitivity, and reduced CSC enrichment post‐chemotherapy.

### 2.4 Efferocytosis is Associated with CSCs and Drives Cancer Stemness in OC

**Figure 4:**
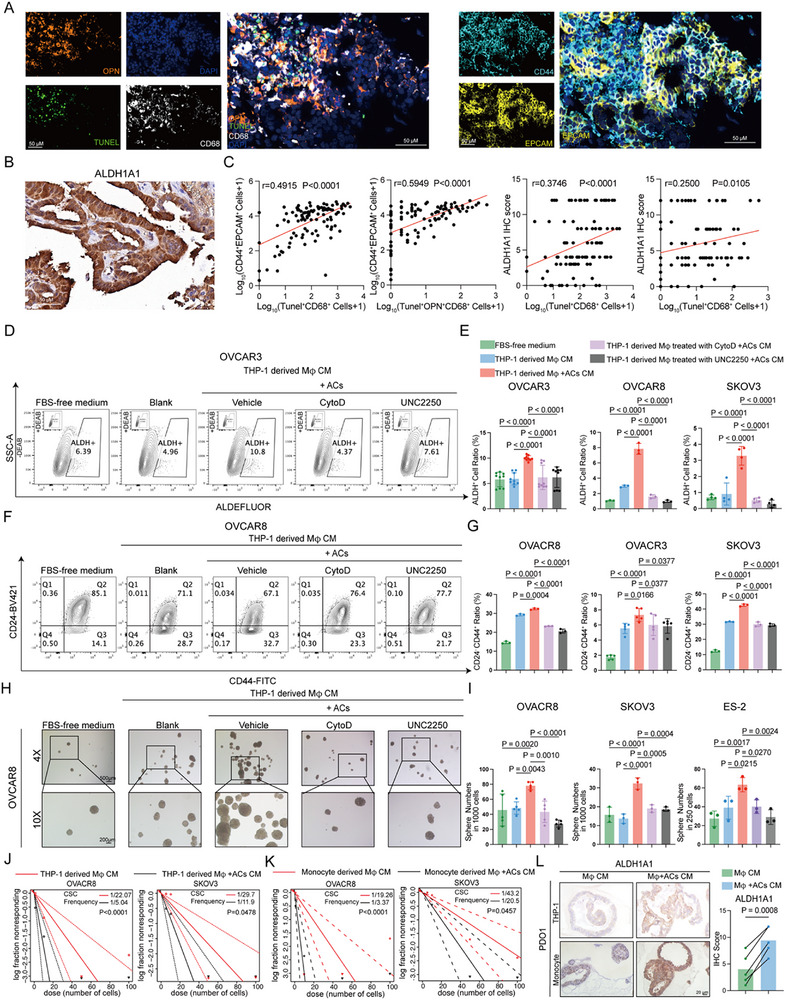
Efferocytosis enhances ovarian cancer stemness. A) Representative mIHC (multiplex immunohistochemistry) images of ovarian cancer samples. Tunel, Green; OPN, orange; CD68, white; CD44, light blue; dark blue, DAPI. B) Representative IHC (immunohistochemistry) images of ALDH1A1 staining in ovarian cancer samples. C) Correlation analysis between Tunel^+^ CD68^+^ cells or OPN^+^ Tunel^+^ CD68^+^ cells, and CD44^+^ EPCAM^+^ cells or ALDH1A1 levels in ovarian cancer samples (n = 104). Statistical significance was tested using Spearman correlation analysis. D) Representative flow cytometry images of ALDEFLUOR assays in OVCAR3 treated with FBS‐free medium, THP‐1 derived Mφ (macrophages) CM, THP‐1 derived Mφ +ACs (apoptotic cells) CM, THP‐1 derived Mφ treated with CytoD (cytochalasin D) +ACs CM, THP‐1 derived Mφ treated with UNC2250 +ACs CM separately for 48 h. The ALDH^+^ gate was identified by the DEAB group. E) Bar graphs showing the proportion of ALDH^+^ cells in OVCAR3 (n = 9 independent experiments), OVCAR8 (n = 3 independent experiments), SKOV3 (n = 4 independent experiments) treated with FBS‐free medium, THP‐1 derived Mφ CM, THP‐1 derived Mφ +ACs CM, THP‐1 derived Mφ treated with CytoD +ACs CM, THP‐1 derived Mφ treated with UNC2250 +ACs CM separately for 48 h. Statistical significance was tested using one‐way ANOVA and Dunnett's multiple comparisons test. F) Representative flow cytometry images of CD24 and CD44 in OVCAR8 treated with FBS‐free medium, THP‐1 derived Mφ CM, THP‐1 derived Mφ +ACs CM, THP‐1 derived Mφ treated with CytoD +ACs CM, THP‐1 derived Mφ treated with UNC2250 +ACs CM separately for 48 h. G) Bar graphs showing the CD24^−^ CD44^+^ cell ratio in OVCAR8 (n = 3 independent experiments), OVCAR3 (n = 5 independent experiments), SKOV3 (n = 3 independent experiments) cells treated with FBS‐free medium, THP‐1 derived Mφ CM, THP‐1 derived Mφ +ACs CM, THP‐1 derived Mφ treated with CytoD +ACs CM, THP‐1 derived Mφ treated with UNC2250 +ACs CM separately for 48 h. Statistical significance was tested using one‐way ANOVA and Dunnett's multiple comparisons test. H) Representative images of sphere formation assays in OVCAR8 treated with FBS‐free medium, THP‐1 derived Mφ CM, THP‐1 derived Mφ +ACs CM, THP‐1 derived Mφ treated with CytoD +ACs CM, THP‐1 derived Mφ treated with UNC2250 +ACs CM separately. I) Bar graphs showing the sphere numbers in OVCAR8 (n = 5 independent experiments), SKOV3 (n = 3 independent experiments), ES‐2 (n = 3 independent experiments) cells treated with FBS‐free medium, THP‐1 derived Mφ CM, THP‐1 derived Mφ +ACs CM, THP‐1 derived Mφ treated with CytoD +ACs CM, THP‐1 derived Mφ treated with UNC2250 +ACs CM separately. Statistical significance was tested using one‐way ANOVA and Dunnett's multiple comparisons test. J) ELDA (Extreme Limiting Dilution Analysis) assays in OVCAR8 and SKOV3 cells treated with THP‐1 derived Mφ CM or THP‐1 derived Mφ +ACs CM. CSC frequency and statistical significance were calculated by using the ELDA software. K) ELDA assays in OVCAR8 and SKOV3 cells treated with monocyte‐derived Mφ CM or monocyte‐derived Mφ +ACs CM. CSC frequency and statistical significance were calculated by using the ELDA software. L) Representative IHC images of ALDH1A1 staining in ovarian cancer PDOs (Patient‐derived organoids) treated with macrophage CM and macrophage +ACs CM. The paired line plots showed ALDH1A1 levels between PDOs treated with macrophage CM and macrophage +ACs CM (n = 5 independent experiments). Statistical significance was tested using the paired t‐test. All data are presented as Mean ± SD.

Our previous study demonstrated that chemotherapy increases the population of ovarian cancer stem cells in humans and mice.^[^ ^8^ ^]^ Given that pharmacological inhibition of efferocytosis during chemotherapy impairs tumor chemoresistance, growth after chemotherapy, and CSCs enrichment in vivo, we sought to further investigate whether efferocytotic macrophages would affect cancer stemness. We first analyzed the correlations between efferocytotic receptors and cancer stemness‐associated genes and found positive correlations between them (Figure S6A, Supporting Information). The mIHC also showed that the number of TUNEL ^+^ CD68^+^ cells and the number of OPN^+^ TUNEL^+^ CD68^+^ cells were significantly positively associated with the number of CD44^+^ EPCAM^+^ cells and ALDH1A1 levels in ovarian cancer tissues (**Figure** **4**A–C). This links efferocytosis to CSC enrichment. Conditioned mediums (CM) were harvested from macrophages and macrophages co‐cultured with apoptotic cells and treated with the vehicle, cytochalasin D, or UNC2250 (Figure S6B, Supporting Information). Cancer cells exposed to CM from efferocytotic macrophages showed increased expression of stemness genes (*SOX2, ALDH1A1, ALDH1A3*), whereas CM from macrophages with blocked efferocytosis had the opposite effect (Figure S6C, Supporting Information). Meanwhile, efferocytotic CM increased the proportion of ALDH^+^ cells (Figure 4D,E; Figure S7A–E, Supporting Information), SOX2 levels (Figure S7F, Supporting Information), the ratio of CD24^−^CD44^+^ cells (Figure 4F,G; Figure S8A, Supporting Information), and increased sphere‐forming capacity (Figure 4H,I; Figure S8B, Supporting Information), all of which were reversed by inhibitors of efferocytosis (Figure 4D–I; Figures S7A–D and S8A,B, Supporting Information). To further test the stem cell frequency in ovarian cancer cells, Extreme Limiting Dilution Analysis (ELDA) was performed, which showed a higher stem cell frequency in efferocytotic CM (Figure 4J,K; Figure S9A,B, Supporting Information). CSCs typically show enhanced chemoresistance.^[^ ^22^ ^]^ Cell viability assessed via MTT assays demonstrated higher IC_50_ values for cisplatin and paclitaxel in ovarian cancer cells cultured with efferocytotic CM than in those cultured with other CMs (Figure S9C, Supporting Information). Finally, we used macrophage CM and efferocytotic CM to treat ovarian cancer PDO. IHC staining revealed elevated CSC markers levels in efferocytotic CM‐treated PDOs compared to those treated with macrophage CM (Figure 4L; Figure S9D–F, Supporting Information). Collectively, these results demonstrate that efferocytosis drives cancer stemness in OC.

### 2.5 Efferocytosis‐Mediated Polyamine Metabolism Induces CSC Enrichment

**Figure 5:**
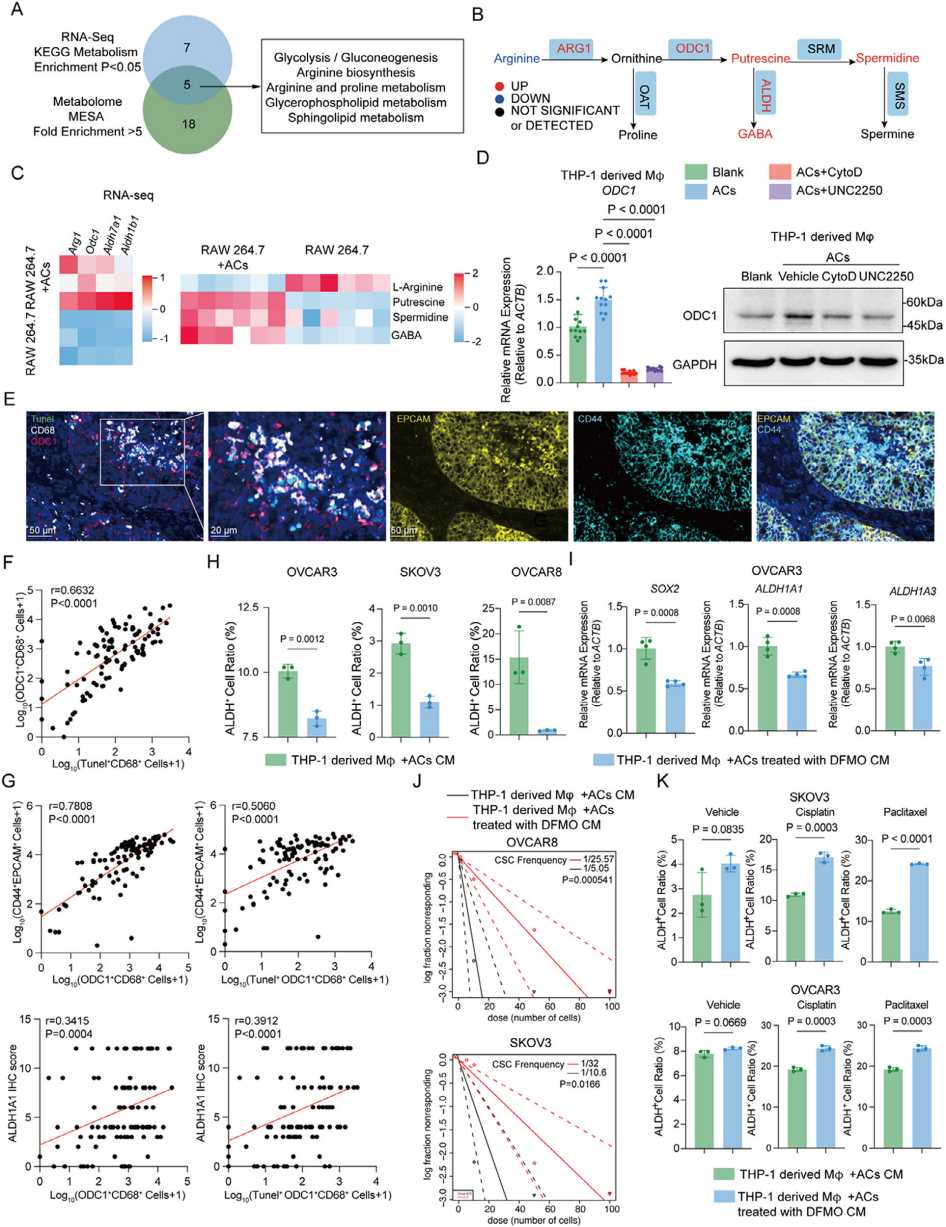
Efferocytosis sustains cancer stemness through polyamine reprogramming. A) Venn Diagram showing the overlapped metabolic pathway between analysis on RNA sequencing and metabolome of RAW 264.7 and RAW 264.7 + ACs (apoptotic cells). B) Metabolic pathway diagram of arginine to polyamines and their downstream metabolites. C) Heatmaps showing differential expression genes and metabolites in polyamine metabolism between RAW 264.7 and RAW 264.7 + ACs. D) Left, qRT‐PCR (Quantitative real‐time PCR) analysis of relative *ODC1* mRNA expression in THP‐1 derived Mφ with different treatments (n = 9 independent experiments). Right, representative western blotting image of ODC1 levels in THP‐1 derived Mφ with different treatments. E) Representative mIHC (multiplex immunohistochemistry) images of ovarian cancer samples. Tunel, Green; ODC1, red; CD68, white; CD44, light blue; DAPI, dark blue; EPCAM, yellow. F) Correlation analysis between Tunel^+^ CD68^+^ cells and ODC1^+^ CD68^+^ cells in ovarian cancer samples (n = 104). G) Correlation analysis between Tunel^+^ ODC1^+^ CD68^+^ cells and CD44^+^ EPCAM^+^ cells or ALDHA1A1 levels in ovarian cancer samples (n = 104). Statistical significance of (F,G) was tested using Spearman correlation analysis. H) Bar graphs showing the proportion of ALDH+ cells in OVCAR3 (n = 3 independent experiments), OVCAR8 (n = 3 independent experiments), SKOV3 (n = 3 independent experiments) treated with THP‐1 derived Mφ +ACs CM, THP‐1 derived Mφ+ACs treated with DFMO CM separately for 48 h. Statistical significance was tested using Student's t‐test. I) Bar graph showing the relative mRNA expression (relative to *ACTB*) of *SOX2* (n = 4 independent experiments), *ALDH1A1* (n = 4 independent experiments), and *ALDH1A3* (n = 4 independent experiments) of OVCAR3 treated with THP‐1 derived Mφ +ACs CM, THP‐1 derived Mφ+ACs treated with DFMO CM separately for 48 h. Statistical significance was tested using Student's t‐test. J) ELDA (Extreme Limiting Dilution Analysis) assays in OVCAR8 and SKOV3 cells treated with THP‐1 derived Mφ+ACs CM or THP‐1 derived Mφ +ACs treated with DFMO CM. CSC frequency and statistical significance were calculated by using the ELDA software. K) Bar graph showing apoptosis cell ratios of SKOV3 (n = 3 independent experiments) and OVCAR3 (n = 3 independent experiments) treated with THP‐1 derived Mφ +ACs CM, THP‐1 derived Mφ+ACs treated with DFMO CM, then received vehicle, paclitaxel, and cisplatin treatments. Statistical significance was tested using Student's t‐test. All data are presented as Mean ± SD.

Because efferocytosis is known to rewire macrophage metabolism, we profiled metabolites in RAW264.7 macrophages after efferocytosis of apoptotic ovarian cells. Liquid chromatography–mass spectrometry (LC–MS) revealed 134 upregulated and 115 downregulated metabolites (Figure S10A–C, Supporting Information). Pathway analysis of combined metabolite and gene expression data highlighted upregulation of arginine‐related metabolism in efferocytotic macrophages (Figures S10D and S11A; **Figure** **5**A). Our RNA‐seq analysis and metabolites showed that arginine decreased in RAW264.7 macrophages with ACs, while its downstream metabolites putrescine, GABA, and spermidine increased (Figure 5B). Meanwhile, the expression of enzymes in arginine and polyamines metabolism, *Arg1*, *Odc1*, *Aldh1b1*, and *Aldh7a1* were also up‐regulated in RAW264.7 macrophages with ACs (Figure 5B). Interestingly, analysis of macrophage metabolic pathways using scMetabolism revealed that the arginine‐related pathways showed increased activity in Macro_c2 (Figure S11B–C, Supporting Information). These results indicated that arginine was converted into polyamines and their derivative metabolites during continuous efferocytosis (Figure 5C). Among them, ODC1 is the rate‐limiting enzyme converting ornithine to putrescine in polyamine synthesis.^[^ ^23^ ^]^ qRT‐PCR and western blotting analyses validated the upregulation of ODC1 in efferocytotic macrophages, and this increase was reversed by efferocytosis inhibitors (Figure 5D; Figure S12A,B, Supporting Information). In ovarian cancer tissues, the number of TUNEL ^+^ CD68^+^ cells was positively correlated with the number of ODC1^+^ CD68^+^ cells, linking efferocytosis to ODC1 upregulation in macrophages (Figure 5E,F). To determine whether cancer stemness maintained by efferocytosis is dependent on polyamine reprogramming in macrophages, we first examined the correlation between ODC1‐positive macrophages and ovarian cancer stem cells. The mIHC analysis showed that the numbers of ODC1^+^ CD68^+^ cells and TUNEL^+^ ODC1^+^ CD68^+^ cells were positively correlated with the number of CD44^+^ EPCAM^+^ cells and ALDH1A1 levels in ovarian cancer tissues, implicating the correlation between macrophage polyamine metabolism and CSCs (Figure 5E–G). To test if inhibiting this pathway affects CSCs, we treated efferocytotic macrophages with DFMO (an ODC1‐selective inhibitor) for 24 h and collected their CM. DFMO‐treated CM significantly reduced the proportion of ALDH^+^ cancer cells and reversed efferocytosis‐induced upregulation of CSC genes (Figure 5H,I; Figure S12B,C, Supporting Information). ELDA assays confirmed that DFMO prevented the increase in CSC frequency induced by efferocytotic CM (Figure 5J; Figure S12D, Supporting Information). Similarly, DFMO‐treated CM increased the apoptosis rate of ovarian cancer cells exposed to cisplatin and paclitaxel (Figure 5K; Figure S12E, Supporting Information). Besides inhibiting ODC1, we also knocked down ODC1 expression in macrophages and found that knockdown of ODC1 in efferocytotic macrophages decreased the proportion of ALDH^+^ cancer cells, SOX2 levels, and CSC frequency (Figure S13A–D, Supporting Information). Efferocytosis‐driven upregulation of ODC1 and polyamine synthesis in macrophages is required to sustain CSCs. Inhibiting ODC1 reverses this effect, linking macrophage polyamine reprogramming to CSC enrichment

### 2.6 Inhibition of Polyamine Synthesis Suppresses Tumor Regrowth, Chemoresistance, and CSC Enrichment In Vivo

**Figure 6:**
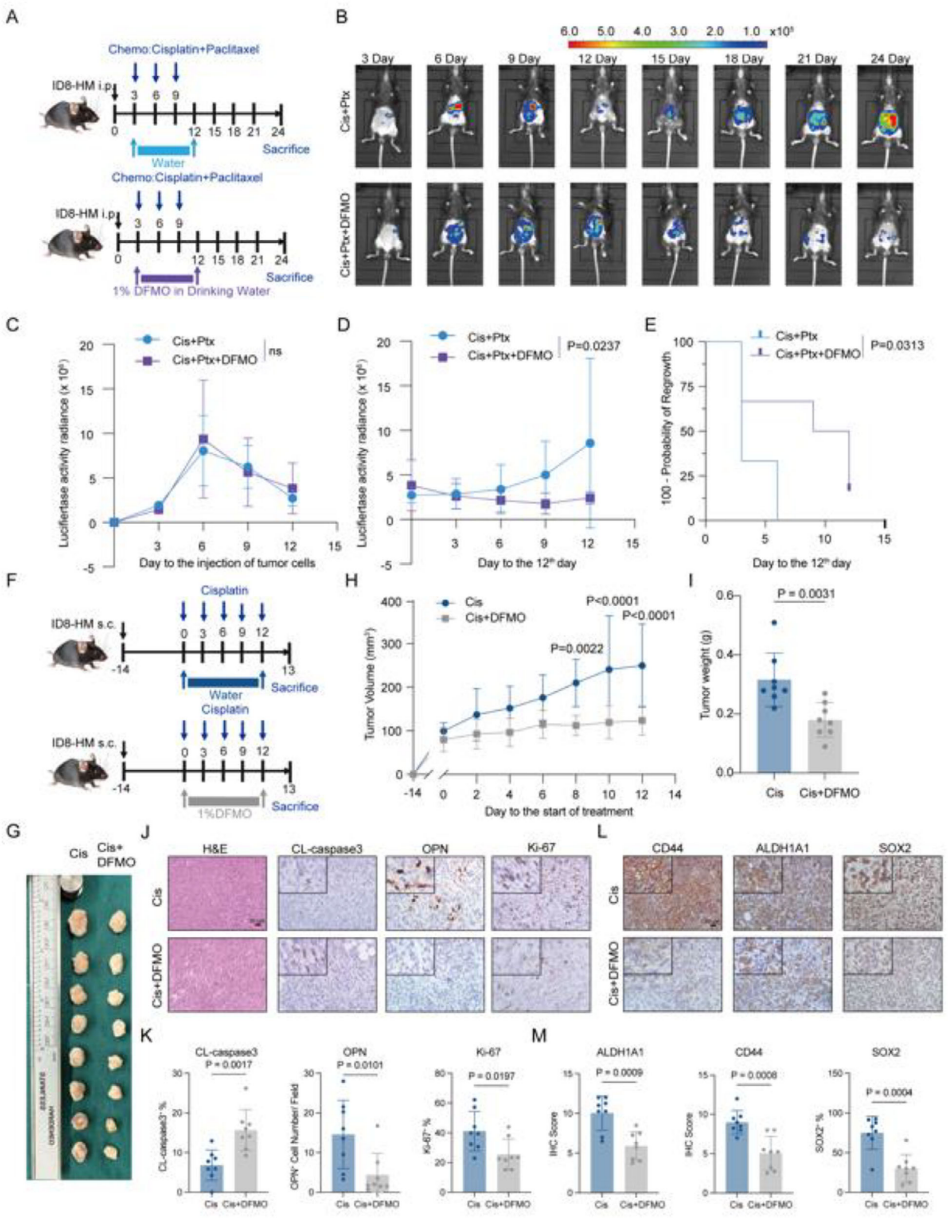
Targeting ODC1 inhibits tumor regrowth and chemoresistance development after chemotherapy in vivo. A) Treatment of ID8‐HM tumor‐bearing mice using cisplatin and paclitaxel combined with ODC1 selected inhibitor DFMO or without DFMO. ID8‐HM cells were intraperitoneally injected into C57BL/6J mice. Treatment of DFMO was initiated at the 3rd day after the injection and sustained for a duration of 9 days. Treatment of cisplatin and paclitaxel was initiated the 3rd day after the injection of tumor cells, then every 3 days for a duration of 9 days. B) Representative images of the intraperitoneal tumor xenografts treated with cisplatin and paclitaxel with DFMO or without DFMO. Tumor burden was assessed by performing in vivo imaging using a live‐imaging system on a schedule of every 3 days. C) Tumor growth curves for mice treated with cisplatin and paclitaxel combined with DFMO (n = 6) or without DFMO (n = 6) from day 0 to day 12. Statistical significance was tested using two‐way ANOVA. ns, not significant. D) Tumor growth curves for mice treated with cisplatin and paclitaxel combined with DFMO (n = 6) or without DFMO (n = 6) after the 12th day. Statistical significance was tested using two‐way ANOVA and Šídák's multiple comparisons test. E) Kaplan–Meier analysis of tumor regrowth for cisplatin and paclitaxel only group (n = 6) and cisplatin and paclitaxel with DFMO group (n = 6). Statistical significance was tested using the log‐rank (Mantel‐Cox) test. F) Treatment of ID8‐HM tumor‐bearing mice with cisplatin and paclitaxel combined with DFMO or without DFMO. ID8‐HM cells were subcutaneously injected into C57BL/6J mice. DFMO treatment was initiated on the 14th day after the injection and sustained for a duration of 12 days. Treatment of cisplatin was initiated on the 14th day after the injection of tumor cells, then every 3 days for a duration of 9 days. G) Representative images of subcutaneous tumors from the ID8‐HM tumor‐bearing mice treated with cisplatin only (n = 8) or cisplatin plus DFMO (n = 8). H) Tumor growth curves of (F), tumor volumes were assessed every 2 days. Statistical significance was tested using two‐way ANOVA and Šídák's multiple comparisons test. I) Bar graph showed the end‐point tumor weights of (F) Statistical significance was tested using Student's t‐test. J) Representative H&E (Hematoxylin and eosin) staining images of Subcutaneous tumors, and representative IHC (immunohistochemistry) images of cleaved‐caspase3, OPN, and Ki‐67 staining in the subcutaneous tumors treated with cisplatin or cisplatin plus DFMO. The images in the upper left corner with double the magnification. K) Bar graphs showing percentage of cleaved‐caspase3^+^, numbers of OPN^+^ cells, and percentage of Ki‐67 ^+^ cells in the subcutaneous tumors treated with cisplatin (n = 8) or cisplatin plus DFMO (n = 8). Statistical significance was tested using Student's t‐test. L) Representative IHC (immunohistochemistry) images of CD44, ALDH1A1, and SOX2 staining in the subcutaneous tumors treated with cisplatin or cisplatin plus DFMO. The images in the upper left corner with double the magnification. M) Bar graphs showing CD44, ALDH1A1 levels, and percentage of SOX2^+^ cells in the subcutaneous tumors treated with cisplatin (n = 8) or cisplatin plus DFMO (n = 8). Statistical significance was tested using Student's t‐test. All data are presented as Mean ± SD.

We next tested whether blocking ODC1 in vivo could prevent chemoresistance, regrowth, and CSC enrichment. In ID8‐HM ovarian cancer models, DFMO alone cannot inhibit tumor growth effectively (Figure S14A, Supporting Information). Then, Mice were treated with cisplatin plus paclitaxel ± DFMO (**Figure** **6**A). During chemotherapy, all treated groups showed tumor growth inhibition (Figure 6B,C). However, after treatment cessation, tumors in the chemo‐only group rapidly regrew (Figure 6D,E), and IHC staining showed an elevated number of OPN^+^ cells and CD44 levels of the chemo‐only group tumors (Figure S14B, Supporting Information). In subcutaneous tumors, mice received cisplatin ± DFMO for 12 days (Figure 6F). The cisplatin +DFMO group had significantly smaller tumors, with lower weight and volume, than cisplatin alone (Figure 6G–I). IHC showed that cisplatin +DFMO tumors had fewer proliferating cells (Ki67^+^ cells) as well as OPN^+^ cells, and more apoptotic cells (cleaved caspase3^+^ cells) than cisplatin‐only (Figure 6J–L). Crucially, DFMO treatment suppressed CSC marker levels (SOX2, ALDH1A1, CD44) in tumors compared to cisplatin alone (Figure 6J–M). These findings indicate that ODC1‐mediated reprogramming of polyamine metabolism in efferocytotic macrophages is essential for cancer stemness promotion induced by efferocytosis.

### 2.7 ODC1‐Putrescine‐OPN Axis in Efferocytotic Macrophage Drives Cancer Stemness in OC Cells

**Figure 7:**
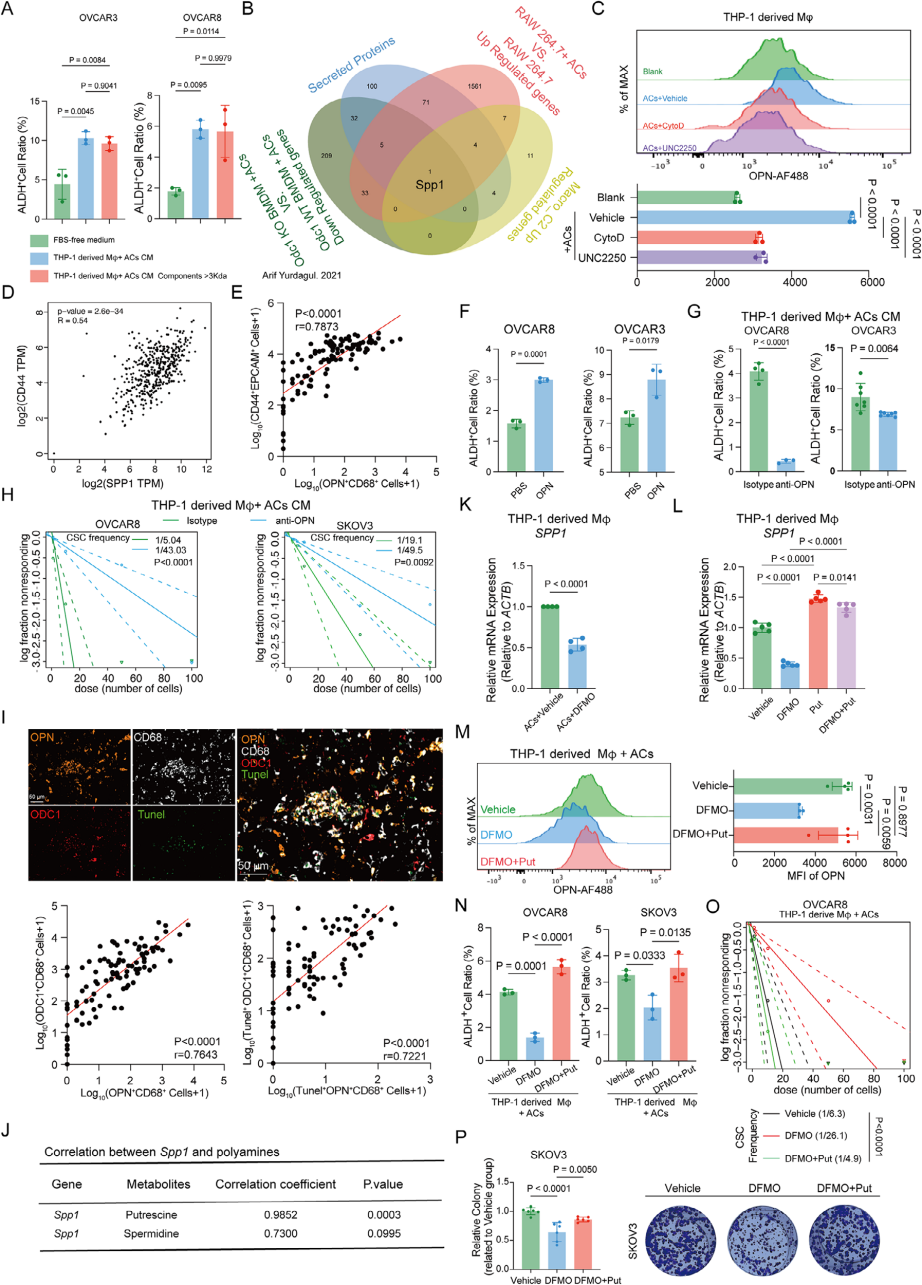
Efferocytotic macrophages upregulate OPN via ODC1‐putrescine axis to sustain ovarian cancer stemness. A) Bar graphs showing the ALDH^+^ cell ratio in OVCAR3 (n = 3, independent experiments), OVCAR8 (n = 3 independent experiments) treated with FBS‐free medium, THP‐1 derived Mφ +ACs CM, THP‐1 derived Mφ+ACs CM components > 3 KDa separately for 48 h. Statistical significance was tested using one‐way ANOVA and Dunnett's multiple comparisons test. B) The Venn Diagram showed the overlapped metabolic genes among efferocytotic Odc1 KO BMDM down‐regulated genes, genes encoding secreted proteins, efferocytotic RAW 264.7 up‐regulated genes, and Macro_c2 up‐regulated genes. C) Representative flow cytometry analysis images of OPN levels in THP‐1 derived Mφ with different treatments and the bar graph (n = 3 independent experiments). Statistical significance was one‐way ANOVA and Dunnett's multiple comparisons test. D) Correlation analysis of *SPP1* expression and *CD44* expression in TCGA‐OV. Statistical significance was tested using Spearman correlation analysis. E) Correlation analysis between OPN^+^ CD68^+^ cells and CD44^+^ EPCAM^+^ cells in ovarian cancer samples (n = 104). Statistical significance was tested using Spearman correlation analysis. F) Bar graphs showing the ALDH^+^ cell ratio in OVCAR3 (n = 3 independent experiments), OVCAR8 (n = 3 independent experiments) treated with PBS and recombinant human OPN separately for 48 h. Statistical significance was tested using the Student t test. G) Bar graphs showing the ALDH^+^ cell ratio in OVCAR3 (n = 7 independent experiments), OVCAR8 (n = 3 independent experiments) treated with THP‐1 derived Mφ +ACs CM+IgG and THP‐1 derived Mφ +ACs CM+anti‐OPN. Statistical significance was tested using the Student t test. H) Representative images of ELDA experiments in OVCAR8 and SKOV3 treated with THP‐1 derived Mφ +ACs CM+IgG and THP‐1 derived Mφ +ACs CM+anti‐OPN. CSC frequency and statistical significance were calculated by using the ELDA software. I) Representative mIHC images of ovarian cancer patients. Tunel, green; ODC1, red; OPN, orange; CD68, white, DAPI, dark blue. And Correlation between OPN^+^ CD68^+^ cells and ODC1^+^ CD68^+^ cells, and correlation between Tunel^+^ OPN^+^ CD68^+^ cells and Tunel^+^ ODC1^+^ CD68^+^ cells in ovarian cancer samples (n = 104). Statistical significance was tested using Spearman correlation analysis. J) Correlation analysis between *Spp1* mRNA and polyamines in RNA sequencing and metabolome of RAW 264.7 and RAW 264.7 + ACs. Statistical significance was tested using Pearson correlation analysis. K) Bar graph showing the relative mRNA expression (relative to *ACTB*) of *SPP1* of THP‐1 derived Mφ +ACs treated with vehicle and DFMO (n = 4 independent experiments). Statistical significance was tested using the Student's t‐test. L) Bar graph showing the relative mRNA expression (relative to *ACTB*) of *SPP1* of THP‐1 derived Mφ treated with vehicle, DFMO, putrescine, and DFMO plus putrescine (n = 5 independent experiments). Statistical significance was one‐way ANOVA and Dunnett's multiple comparisons test. M) Representative flow cytometry analysis images of OPN levels in THP‐1 derived Mφ +ACs with different treatments and the bar graph (n = 4 independent experiments). Statistical significance was one‐way ANOVA and Dunnett's multiple comparisons test. N) The bar graphs showed the ALDH^+^ cell ratio in OVCAR8 and SKOV3 (n = 3 independent experiments) treated with THP‐1 derived Mφ +ACs CM, THP‐1 derived Mφ +ACs +DFMO CM, and THP‐1 derived Mφ +ACs +DFOM +putrescine CM separately for 48 h. O) OVCAR8 cells in a serial dilution were treated with THP‐1 derived Mφ +ACs CM, THP‐1 derived Mφ +ACs +DFMO CM, and THP‐1 derived Mφ +ACs +DFOM +putrescine CM separately. CSC frequency and statistical significance were calculated by using the ELDA software. P) Representative images of colony formation assays in SKOV3 cells treated with THP‐1 derived Mφ +ACs CM, THP‐1 derived Mφ +ACs +DFMO CM, and THP‐1 derived Mφ +ACs + DFMO +putrescine CM separately. Statistical significance was tested using one‐way ANOVA and Dunnett's multiple comparisons test. All data are presented as Mean ± SD. Put, putrescine.

Growing evidence has highlighted the critical role of polyamines in regulating cancer stemness.^[^ ^24^, ^25^ ^]^ We investigated how polyamine metabolism in macrophages regulates efferocytosis‐driven cancer stemness. First, we tested whether exogenous polyamines associated metabolites could mimic the effect of efferocytotic CM. OC cells were cultured with putrescine, spermidine, or GABA (Figure S14C–E, Supporting Information). Only GABA increased the ALDH^+^ cell ratio of OVCAR3 cells, rather than SKOV3 and OVCAR8 cells, while putrescine and spermidine did not affect the ALDH^+^ cell ratio of OC cells. This suggests that secreted polyamines are not the CSC‐promoting factor in OC. We therefore hypothesized that secreting proteins regulate cancer stemness. To test whether the secreted proteins induced cancer stemness, ovarian cancer cells were cultured with efferocytotic CM or with components (>3kDa) after efferocytotic CM ultrafiltration (Figure S14A, Supporting Information). The results showed that both the components and efferocytotic CM elevated the ratio of ALDH^+^ cells in OVCAR3 and OVCAR8 cells, indicating involvement of secreted proteins (**Figure** **7**A; Figure S15A,B, Supporting Information).

Our results, as depicted in the Venn diagram, highlight the overlapping genes among four distinct datasets: Odc1 knockout (KO) BMDMs with ACs down‐regulated genes, RAW264.7 cells with ACs up‐regulated genes, Macro_c2 up‐regulated genes, and secreted protein‐coding genes. Spp1 was identified as the only common gene across all datasets (Figure 7B). To validate this finding, we performed Western blotting and flow cytometry analysis, both of which confirmed that efferocytosis significantly increases OPN levels in macrophages. (Figure 7C; Figure S15C, Supporting Information). Survival analysis in public datasets showed that *SPP1* expression shortened the overall survival of ovarian cancer patients (Figure S15D, Supporting Information), and similarly, in our cohort, OPN^+^ CD68^+^ cells also correlated with shorter OS and PFS (Figure 1K).

Moreover, analysis from the Gepia2 database showed a positive correlation between *SPP1* and genes associated with cancer stemness (Figure 7D; Figure S15E, Supporting Information), and OPN^+^ CD68^+^ cells were positively correlated with CD44^+^ EPCAM^+^ cells in our cohort (Figure 7E). To determine whether OPN was necessary to promote efferocytosis–mediated ovarian cancer stem‐like phenotypes, we treated ovarian cancer cells with recombinant human OPN and assessed the stem‐like phenotype. Exogenous OPN enhanced the ratio of ALDH^+^ cells in ovarian cancer cells (Figure 7F; Figure S16A, Supporting Information), consistent with previous reports.^[^ ^26^ ^]^ Conversely, neutralizing OPN in efferocytotic CM reduced the ALDH^+^ ratio (Figure 7G; Figure S16B,C, Supporting Information) and decreased CSC frequency in treated OC cells (Figure 7H; Figure S16D, Supporting Information). Because the OPN‐CD44 axis promotes ovarian cancer stemness,^[^ ^26^ ^]^ we examined the level of CD44 in our ovarian cancer cell lines (Figure S17A, Supporting Information). Our results showed that CD44 blockade and knockdown of CD44 in ovarian cancer cells inhibited the effect of OPN and efferocytotic CM (Figure S17B‐E, Supporting Information). These data indicate that efferocytotic macrophage‐derived OPN is necessary for cancer stemness driven by efferocytosis.

We hypothesized that the overexpression of OPN in efferocytotic macrophages relied on efferocytosis‐induced upregulation of ODC1 and polyamines. In our cohort, OPN^+^ CD68^+^ cells were positively correlated with ODC1^+^ CD68^+^ cells, and TUNEL ^+^ OPN^+^ CD68^+^ cells were positively correlated with TUNEL ^+^ ODC1^+^ CD68^+^ cells in ovarian cancer tissues (Figure 7I). Polyamines have been reported to combine with DNA, RNA, and proteins to participate in biological processes.^[^ ^10^, ^27^, ^28^ ^]^ Indeed, combined analysis of metabolites and transcripts showed that putrescine levels (but not spermidine) strongly correlated with *SPP1* mRNA expression (Figure 7J). qRT‐PCR and western blotting analyses confirmed a decrease in *SPP1* and OPN levels in DFMO‐treated macrophages, which could be rescued by putrescine (Figure 7K,L; Figure S18ASupporting Information). Given that putrescine is reported to stabilize mRNA,^[^ ^10^ ^]^ we hypothesized that putrescine, produced from ornithine by ODC1, might maintain *SPP1* stability, thereby promoting OPN overexpression. To test this, we treated macrophages with actinomycin‐D and analyzed *SPP1* expression at various time points. qRT‐PCR data showed that the decay of *SPP1* was slowed by ACs, accelerated by DFMO treatment, and rescued by putrescine treatment of DFMO‐treated cells (Figure S18B, Supporting Information), indicating that efferocytosis and putrescine metabolism regulated by ODC1 may impact *SPP1* RNA stability. Furthermore, putrescine rescued the downregulation of OPN induced by DFMO in efferocytotic macrophages (Figure 7M). Putrescine also rescued the decrease in the ALDH^+^ cell ratio, CSC frequency of OC cells, and colony formation induced by DFMO (Figure 7N–P; Figure S18C‐F, Supporting Information), and the decrease in the ALDH^+^ cell ratio induced by ODC1 knockdown of efferocytotic macrophages. In summary, our results demonstrate that the efferocytosis‐ODC1‐putrescine pathway enhances the stability of *SPP1* mRNA and upregulates OPN to promote ovarian cancer cell stemness.

### 2.8 ODC1 Inhibitor Sensitizes Tumors to Cisplatin and Mitigates CSC Enrichment in PDX Model

**Figure 8:**
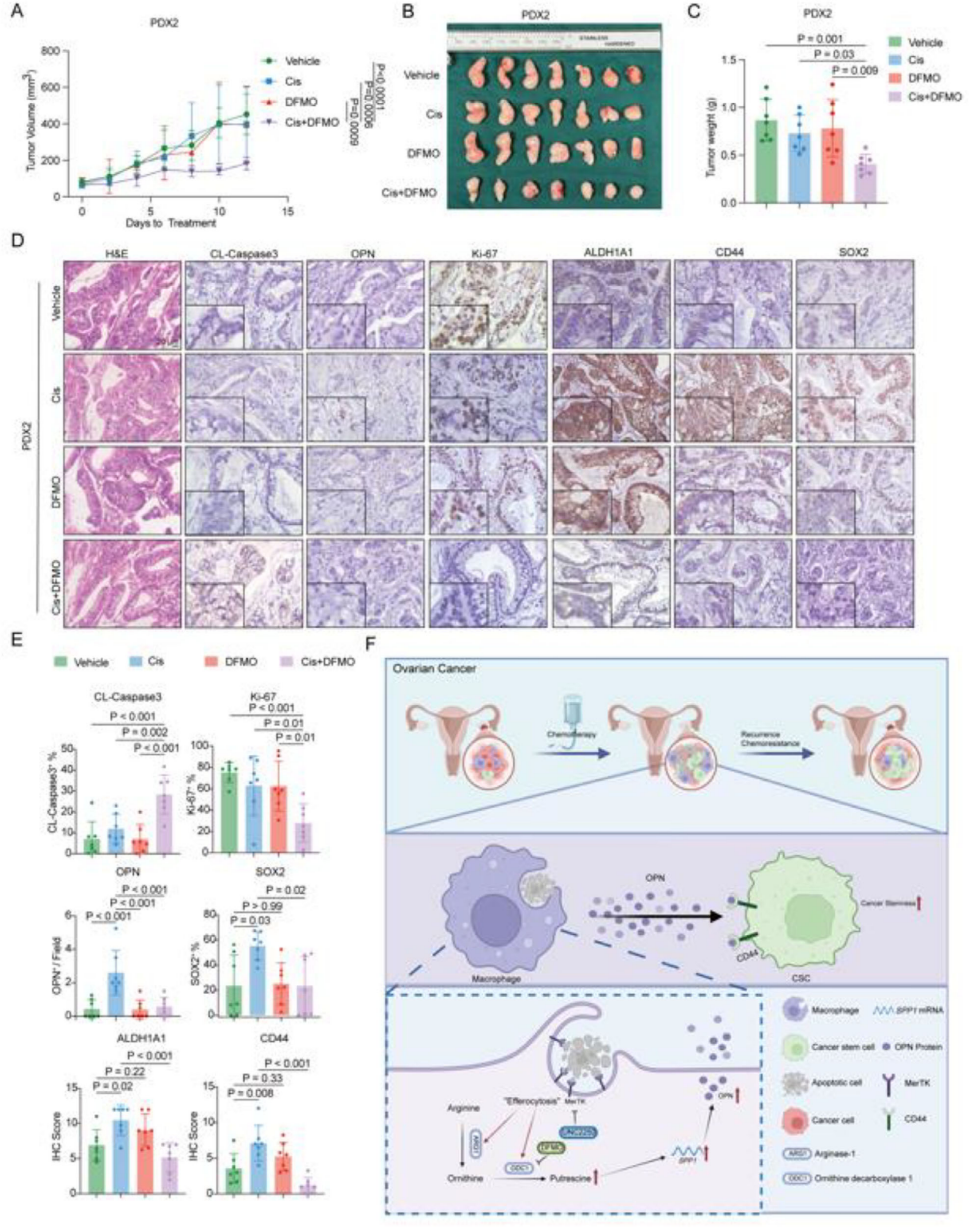
ODC1 blockade boosts anti‐tumor effects of cisplatin and inhibits cisplatin‐induced CSCs enrichment. A) Tumor growth curves for patient‐derived xenograft (PDX) mice treated with vehicle (n = 7), cisplatin (n = 7), DFMO (n = 7), or cisplatin combined with DFMO (n = 7). Tumor volumes were measured every 2 days. Statistical significance was assessed using two‐way ANOVA with Dunnett's multiple comparisons test. B) Representative images of tumors from PDX mice treated with vehicle (n = 7), cisplatin (n = 7), DFMO (n = 7), or cisplatin combined with DFMO (n = 7). C) Tumor weights of (B) Statistical significance was assessed using one‐way ANOVA and Dunnett's multiple comparisons test. D) Representative H&E (Hematoxylin and eosin) staining images of tumors from PDX mice treated with vehicle (n = 7), cisplatin (n = 7), DFMO (n = 7), or cisplatin combined with DFMO (n = 7). And representative IHC (immunohistochemistry) images of cleaved‐caspase3 staining, OPN staining, Ki67 staining, ALDH1A1 staining, CD44 staining, and SOX2 staining in tumors from PDX mice treated with vehicle (n = 7), cisplatin (n = 7), DFMO (n = 7), or cisplatin combined with DFMO (n = 7). The images in the lower left corner with double the magnification. E) Bar graph showed the statistical results of (D) Statistical significance was one‐way ANOVA and Dunnett's multiple comparisons test. F) Schematic of how efferocytosis fuels polyamine metabolism, upregulates OPN expression, and promotes cancer stemness (Created by biorender.com). All data are presented as Mean ± SD.

Our data establish that efferocytotic macrophages promote ovarian cancer stemness through the efferocytosis–ODC1–putrescine–OPN signaling axis during chemotherapy. While several agents targeting efferocytosis are under clinical evaluation,^[^ ^29^ ^]^ the FDA‐approved ODC1 inhibitor DFMO is clinically available for neuroblastoma treatment.^[^ ^30^ ^]^ We have demonstrated that DFMO enhanced cisplatin efficacy and inhibited CSC enrichment in syngeneic models. To further assess its translational potential, we employed PDX models generated from mucinous ovarian cancer and evaluated the therapeutic effect of DFMO combined with cisplatin (Figure S19A‐B Supporting Information). Neither DFMO nor cisplatin alone significantly reduced tumor growth in the PDX models; however, their combination markedly suppressed tumor progression and decreased tumor weight relative to monotherapies (**Figure** **8**A–C). Correspondingly, IHC revealed that the combination treatment reduced the Ki67^+^ cell ratio as well as the number of OPN^+^ cells, and increased the cleaved‐caspase3^+^ cell ratio (Figure 8D,E). To investigate the enrichment of cancer stemness under treatment, IHC for ALDH1A1, CD44, and SOX2 was performed. As expected, cisplatin induced upregulation of ALDH1A1 levels, CD44 levels, and the SOX2^+^ cell ratio in tumors. In contrast, tumors from the PDX under combination treatments showed a significant reduction in these CSC markers (Figure 8D,E). These observations support the notion that DFMO combined with chemotherapy enhances chemotherapy effectiveness by halting the enrichment of cancer stemness.

## 3 Discussion

Chemotherapy‐induced enrichment of cancer stem cells plays a pivotal role in the recurrence and chemoresistance of EOC. While prior studies have focused on the selection of pre‐existing CSCs or dedifferentiation of cancer cells as mechanisms of enrichment,^[^ ^3^ ^]^ our findings reveal that chemotherapy‐induced efferocytosis is a key contributor to CSC enrichment. Efferocytosis‐driven upregulation of ODC1 and the subsequent production of putrescine enhanced the RNA levels of *SPP1* and the protein levels of OPN in macrophages, thereby fueling ovarian cancer stemness through the OPN‐CD44 axis (Figure 8F). These findings highlight the importance of efferocytosis in the enrichment of cancer stem cells and identification of the efferocytosis‐putrescine‐OPN axis, and suggest potential therapeutic targets for overcoming chemoresistance and recurrence in EOC.

Efferocytosis is a central biological process that balances immune responses and tissue repair to maintain homeostasis.^[^ ^15^, ^31^ ^]^ Some studies have reported efferocytosis‐induced immune suppression and cancer progression.^[^ ^16^, ^32^ ^]^ In our study, enrichment analyses using both single‐cell RNA sequencing and bulk RNA sequencing consistently demonstrated the immune‐inhibitory capacity of efferocytotic macrophages. During cisplatin‐based chemotherapy, MerTK inhibition significantly extended the regrowth‐free time and enhanced the efficacy of chemotherapy. These effects may be partially attributed to immune activation. However, Werfel et al. found that MerTK inhibition did not block immunosuppressive changes in response to tumor cell death.^[^ ^16^ ^]^ Therefore, we hypothesized that in addition to immunosuppression, efferocytosis might exert other effects that contribute to tumor regrowth and cisplatin resistance. Tissue repair macrophages can activate stem cells to repair tissues and promote regeneration. For instance, macrophages phagocytose dying hepatocytes and secrete Wnt3a into liver progenitor cells to facilitate tissue repair and regeneration.^[^ ^9^ ^]^ macrophages phagocytose dying hepatocytes and secrete Wnt3a into liver progenitor cells to facilitate tissue repair and regeneration.^[^ ^9^ ^]^ Given that CSCs are a major driver of drug resistance and tumor recurrence,^[^ ^33^, ^34^, ^35^ ^]^ we tested the hypothesis that efferocytosis might activate CSCs. Our findings revealed that efferocytotic macrophages secrete OPN to promote stemness in ovarian cancer cells. This discovery highlights the previously underappreciated role of efferocytosis in cancer progression beyond its immunosuppressive effects.

Efferocytosis induces metabolic reprogramming in macrophages to facilitate tissue repair.^[^ ^10^, ^36^, ^37^, ^38^ ^]^ Our data demonstrate that efferocytosis drives a shift in arginine metabolism, promoting the conversion of arginine to putrescine. Yurdagul et al. demonstrated that efferocytotic macrophages metabolize AC‐derived arginine and ornithine to putrescine.^[^ ^10^ ^]^ Astuti et al. demonstrated that efferocytosis upregulated ARG1 expression.^[^ ^32^ ^]^ Consistently, our data showed that arginine was metabolized into putrescine, and the associated metabolic enzymes, including ARG1 and ODC1, were upregulated. Odc1 is a key rate‐limiting enzyme in polyamine metabolism.^[^ ^10^, ^23^ ^]^ We reported that efferocytosis promotes ODC1 overexpression, enhancing putrescine levels. However, the underlying mechanisms remain unclear. ODC1 is a direct transcriptional target of MYC.^[^ ^39^ ^]^ A recent study showed that nucleotides derived from the hydrolysis of apoptotic cell DNA activate the DNA‐PKcs‐mTORC2/Rictor pathway, which increases Myc expression in efferocytotic macrophages.^[^ ^40^ ^]^ This pathway may contribute to the upregulation of ODC1. Putrescine has been reported to promote RNA stability.^[^ ^10^ ^]^ Our results revealed a positive correlation between putrescine levels and SPP1 mRNA expression, and preliminary experiments indicated that putrescine promoted the stability of *SPP1* mRNA. Moreover, using the ODC1 inhibitor DFMO in both in vivo and in vitro experiments, we linked polyamine metabolism in macrophages to cancer stemness, further elucidating the role of efferocytosis in promoting cancer progression.

*SPP1* and *CXCL9* are crucial determinants and indicators of tumor‐associated macrophage polarity.^[^ ^19^ ^]^ Consistently, these two polarized subtypes of macrophages were found in our data and showed opposite survival associations. Surprisingly, the *Spp1* ^+^ macrophage ratio was upregulated in the post‐chemotherapy group. SPP1^+^ and *CXCL9* ^+^ macrophages have been identified as phagocytic macrophages.^[^ ^21^ ^]^ However, compared to *CXCL9* ^+^ macrophages, *SPP1* ^+^ macrophages are unable to process and present antigens, as observed in both this study^[^ ^21^ ^]^ and our results. We linked efferocytosis, polyamine metabolism, and *SPP1* ^+^ macrophages in ovarian cancer, providing new ideas for targeting such macrophages. However, whether the proportion of SPP1^+^ macrophages can be reduced by targeting polyamine metabolism and efferocytosis in other cancers remains unclear.

DFMO, an irreversible inhibitor of ODC1, has been approved for the treatment of human African trypanosomiasis. The U.S. Food and Drug Administration approved the use of DFMO for the treatment of high‐risk neuroblastomas in adults and children who have had at least a partial response to multimodal therapy, including anti‐GD2 immunotherapy.^[^ ^30^, ^41^ ^]^ However, in in vitro experiments, the CRISPR knockdown effect of ODC1 was very modest in 1100 human cell lines that were not sensitive to the loss of function of ODC1, and the absence of ODC1 was not associated with the efficacy of DFMO treatment in vitro.^[^ ^42^ ^]^ Some early studies confirmed that DFMO inhibited the proliferation and induced apoptosis of ovarian cancer cells.^[^ ^43^, ^44^ ^]^ The inconsistency between ex vivo and in vivo experiments suggests that the mechanism of action of DFMO in vivo may be different from that in vitro. One study reported that mouse CD8^+^ T cells deficient in ODC1 inhibited the growth of triple‐negative breast cancer cells.^[^ ^45^ ^]^ In a mouse ovarian cancer model, DFMO in combination with azacitidine increased the proportion of M1 macrophages in tumors and inhibited tumor growth.^[^ ^46^ ^]^ Our study extends these findings by showing that DFMO prevents chemotherapy‐induced CSC enrichment and overcomes chemoresistance in both immunodeficient and immunocompetent ovarian cancer models. DFMO's established safety profile makes it an attractive candidate for combination therapy with platinum‐based chemotherapy in clinical trials

## 4 Experimental Section

### Human Samples

The protocol for the human study was reviewed and approved by the Ethics Committee of the Union Hospital, Tongji Medical College, Huazhong University of Science and Technology (Wuhan, China), and informed consent was obtained from all patients. All patients were pathologically diagnosed with primary ovarian cancer, without other malignant diseases or conditions associated with abnormal immunity or hematopoietic function. Human ovarian cancer tissue specimens were obtained from patients with ovarian cancer who had undergone surgical resection. These specimens were used to establish PDX models, perform mIHC analysis, or generate patient‐derived organoids (PDOs). Blood samples were collected from patients diagnosed with benign gynecological diseases to obtain peripheral blood mononuclear cells (PBMCs) and isolate CD14^+^ monocytes.

### Establishment of PDX

NVSG female mice (NOD‐Prkdcscid Il2rgtm201(‐/‐)/V, VIEWSOLID BIOTECH) were used to establish ovarian cancer patient‐derived xenograft (PDX) models. Tumors were obtained from patients with ovarian cancer, after which the tumor tissues were dissected into smaller fragments under aseptic conditions. The tumor fragments were immediately implanted subcutaneously into mice to generate an ovarian cancer PDX model. The clinical information of patients whose ovarian tumor samples were used to establish PDX models is provided in Table S1 (Supporting Information).

### Single‐Cell Sequencing

After establishing the PDX model, intraperitoneal injections of vehicle, cisplatin (3 mg kg^−1^, MCE, HY‐17394), paclitaxel (10 mg kg^−1^, MCE, HY‐B0015), or a combination of cisplatin (3 mg kg^−1^) and paclitaxel (10 mg kg^−1^) were administered every 3 days. Following treatment, subcutaneous tumors were collected from treated mice for single‐cell sequencing. The tumors were dissected into fragments—2–4 mm^3^ fragments. Subsequent digestion was performed following the manufacturer's protocols for the human tumor dissociation kit (Miltenyi Biotec, 130‐095‐929) and gentleMACS Dissociator (Miltenyi Biotec, 130‐093‐235). The single‐cell suspension was loaded into chromium microfluidic chips using Chromium Single Cell 30 v3 chemistry and barcoded using a 10× Chromium Controller (10X Genomics). RNA was reverse‐transcribed, and sequencing libraries were constructed using the Chromium Single Cell 30 v3 reagent kit (10X Genomics), following the manufacturer's instructions. Sequencing was performed on the Illumina NovaSeq platform (Beijing Novogene Technology Co., Ltd.).

### Single‐Cell RNA Sequencing Data Processing

FastQC (Version 0.12.0) was used to assess the quality of raw reads and to remove low‐quality reads when necessary. Raw reads were demultiplexed and mapped to the reference genome mm10 using the 10X Genomics Cell Ranger pipeline with default parameters. Seurat (R package version 4.1.1) was used for downstream analyses. Cells with fewer than 500 or more than 6000 detected genes, as well as those with a mitochondrial content exceeding 20%, were excluded. SCTransform was used to normalize library sizes. PCA dimensionality reduction was performed using the RunPCA function in Seurat. Multicellular samples were identified using DoubletFinder (version 2.0.3) and were removed. Cells were clustered using the FindNeighbors and FindClusters functions with a resolution of 0.1 and visualized using RunUMAP in Seurat. Marker genes were identified using the FindAllMarker function. Subsequently, clusters were annotated based on marker genes, including fibroblasts (*Col1a1*, *Col1a2*, *Pdgfra*, *Dcn*, *Acta2*), macrophages (*Ptprc*, *Cd14, Ly6c2, Cd68, Lyz2*), endothelial cells (*Pecam1, Vwf, Cdh5*), dendritic cells (*Ptprc, Cd74, H2‐Aa, H2‐Ab1, H2‐Eb1*), and neutrophils (*Ptprc, S100a8, S100a9, Ly6g, Csf3r*). Macrophages were selected for further analyses. After normalization and PCA, harmony (version 0.1.0) was applied to correct batch effects. Additionally, the macrophages were reclustered at a resolution of 0.1. Subclusters were identified using marker genes determined using the FindAllMarkers function in Seurat. All the parameters used, except those mentioned above, were used as defaults. The results were visualized using ggplot2 (version 3.4.3), scRNAtoolVis (version 0.0.4), and SCP (version 0.5.1).

### Multiplex Immunohistochemical (mIHC)

Slides of the ovarian cancer tissue chip were incubated with anti‐EPCAM (ab223582, Abcam, 1:1000, RRID:AB_2762366), anti‐CD44 (#37259, Cell Signaling Technology, 1:500, RRID:AB_2750879), anti‐CD68 (ab213363, Abcam, 1:1000, AB_2801637), anti‐OPN (ab214050, Abcam, 1:1000, AB_2894860), and anti‐ODC1 (ab270268, Abcam, 1:500) antibodies using the Think color‐6 color mIHC kit (Freethinking, FH36100R) according to the manufacturer's instructions, followed by terminal deoxynucleotidyl transferase nick‐end labeling (TUNEL) staining using the TUNEL staining kit (KGA1401‐20, Keygen Biotech) according to the manufacturer's instructions. Finally, the slides were counterstained with DAPI to visualize the nuclei. The slides were scanned using a slice scanner (Pannoramic MIDI; 3Dhistech, Hungary), and the images were analyzed using HALO Highplex FL (Indica Labs; Albuquerque, NM). The percentage of positive cells was calculated using HALO. The clinical and pathological parameters of the patients for mIHC analysis are provided in Table S3 (Supporting Information).

### Efferocytosis Assays

A2780 and OVACR3 cells were treated with 50 µm Cisplatin and 50 nm Paclitaxel for 48h to get apoptotic ovarian cancer cells. The Apoptotic ovarian cancer cells were incubated with 5 µm Did (MCE, HY‐D1028) for 30 min and added to macrophages stained with anti‐human CD45‐V500 (BD, 560777, RRID:AB_1937324) or anti‐mouse F4/80‐BV421 (Biolegend, 123131, RRID:AB_10901171) at a ratio of 5:1, then incubated for 24h or 3 h at 37  °C with 5% CO2, washed third with PBS, and scraped with cell scrapers to analyze the efferocytosis capacity by flow cytometry. CD45^+^ and Did^+^ cells, or F4/80^+^ and Did^+^ cells, were identified as efferocytotic macrophages. Each experiment was performed with at least three biological replicates. The FlowJo software (10.4.1) was used for all data analyses.

### Preparation of Macrophage‐Conditioned Medium (CM)

Macrophages were incubated with the apoptotic ovarian cancer cells at a ratio of 1:5, treated with vehicle, 2 µm Cytochalasin D (Invitrogen, PHZ1063), 10 µm UNC2250 (MCE, HY‐15797 ) for 24h at 37  °C with 5% CO2, then washed third with PBS to remove apoptotic cells and drugs. Efferocytotic macrophages were treated with 1 mM DFMO (MCE; HY‐B0744) for 24h, then washed third with PBS to remove DFMO. These macrophages were cultured for 48h in serum‐free RPMI‐1640, and then the culture medium was collected as a macrophage‐conditioned medium (CM) for ovarian cancer cell culture. The collected CM was filtered using a 0.22‐mm filter (Biosharp, BS‐PES25‐22‐S) and stored at −80  °C.

### Flow Cytometry

Flow cytometry was used to detect the stem cell markers CD44 and CD24 using anti‐CD44‐FITC (Elabscience, E‐AB‐F1100C) and anti‐CD24‐BV421 (Biolegend, 311121, RRID:AB_10915556) in ovarian cancer cells. CD44^+^ and CD24^−^ ovarian cancer cells have been identified as ovarian cancer stem cells. The ALDEFLUOR Kit (STEMCELL Technologies, catalog no. 01700) was used to determine ALDH activity in ovarian cancer cells, following the manufacturer's instructions. ALDH^+^ ovarian cancer cells were identified as ovarian cancer stem cells (CSCs). The CD44^+^ and CD24^−^ cell rates and ALDH+ cell rates were measured using a flow cytometer. Anti‐OPN (Proteintech, 22952‐1‐AP, RRID:AB_2783651) was used to analyze the OPN levels in macrophages. Each experiment was performed with at least three biological replicates. The FlowJo software (10.4.1) was used for all data analyses.

### Sphere Formation Assay

Ovarian cancer cells cultured with macrophage CM were resuspended and placed into the 6‐well ultralow plate (Corning, 3471) in serum‐free DMEM/F12 medium supplemented with 20 ng mL^−1^ human EGF (R&D, 236‐EG), 20 ng mL^−1^ human bFGF (R&D, DFB50), and 2% B27 supplement (BasoMedia, S440J7) for 7–14 days at 37 °C with 5% CO_2_. The stem cell medium was supplemented every three days, and the spheres were counted on the final day. Each experiment was performed with at least three biological replicates.

### Extreme Limiting Dilution Analysis (ELDA)

OVCAR8 cells cultured with macrophage CM were resuspended at a density of 100 cells/well, 50 cells/well, 10 cells/well, and 1 cell/well. SKOV3 cells cultured with macrophage CM were resuspended at a density of 100 cells/well, 50 cells/well, 10 cells/well, and 5 cells/well placed into a 96‐well ultralow plate (Corning,7007) in sphere formation medium for 7–14 days at 37 °C with 5% CO_2_. Sphere formation was assessed on the final day, and the data were input into the ELDA online software (http://bioinf.wehi.edu.au/software/elda/) for the analysis of CSC frequency and statistical significance.^[^ ^47^ ^]^

### Statistical Analysis

GraphPad Prism 9 software was used for statistical analysis, IC50 calculations, and survival analysis. For all survival analyses, patients were divided into low‐ratio and high‐ratio groups based on the optimal cut‐off value calculated using the R package survminer (version 0.5.0). Statistical significance was determined using the log‐rank (Mantel‐Cox) test. For comparisons between two groups, Student's t‐test or the non‐parametric Mann‐Whitney U test was employed, depending on data distribution. For multiple group comparisons, one‐way or two‐way analysis of variance (ANOVA) was used, followed by appropriate post‐hoc tests to evaluate differences. Spearman's correlation was applied to assess the relationship between two continuous variables. Significance was defined as P < 0.05. Data are presented as means ± standard deviation (SD), with error bars indicating SD. All data were derived from at least three independent experiments.

### Ethics Approval Statement

This study was approved by the Ethics Committee of the Union Hospital, Tongji Medical College, Huazhong University of Science and Technology (Wuhan, China) (project number: [2024] (118)). Written informed consent was obtained from all patients. The animal study was carried out in compliance with the guidance of the Animal Care Committee of the Tongji Medical College, Huazhong University of Science and Technology (Wuhan, China) (approval number: [2024]4767).

## Conflict of Interest

The authors declare no conflict of interest.

## Author Contributions

Wenhan Li, Feiquan Ying., and Xinkai Pang contributed equally to this work. W.L., J.C., and S.S. contributed to the conceptualization of the study. Methodology was developed by W.L., F.Y., T.L., and P.L. The investigation was carried out by X.P., Q.W., G.L., Y.G., and X.X. Visualization was performed by L.H., J.X., X.L., X.W., and S.T. Funding was acquired by S.D., F.Y., L.C., and J.C. The project was supervised by J.C., Y.Z., L.C., and Y.W. W.L. and X.P. prepared the original draft, and J.C. and W.L. were responsible for review and editing.

## Supporting information



Supporting Information

Supporting Information

Supporting Information

Supporting Information

Supporting Information

Supporting Information

## Data Availability

Single‐cell RNA sequencing and bulk RNA sequencing data generated in this study are publicly available in the Genome Sequence Archive (GSA) under the accession numbers HRA011200, CRA025015, and CRA025002. All other raw data are available upon request from the corresponding authors.
