# Supplementary material for: Efferocytosis‐Driven Polyamine Metabolism in Macrophages Enhances Cancer Stem Cell Enrichment after Chemotherapy in Ovarian Cancer
Source: Adv Sci (Weinh). 2025 Nov 21;13(8):e12508. doi: 10.1002/advs.202512508 (PMC12884752; doi:10.1002/advs.202512508)
Supplement: Supplementary file 1 — Supporting Information [file ADVS-13-e12508-s006.pdf]

Bulk RNA sequencing

Total RNA was extracted from subcutaneous tumors of PDX models undergoing chemotherapy and from RAW264.7 cells incubated with or without apoptotic cells using RNAiso Plus (#9109, Takara, Japan). The extracted RNA was applied to Oligo(dT) magnetic beads to enrich mRNA with poly A tails. Subsequently, the mRNA was randomly fragmented in the presence of divalent cations using NEB Fragmentation Buffer. Fragmented mRNA was transcribed into cDNA, which was amplified to construct the sequencing library. After quality assessment and pooling, the library was sequenced on an Illumina NovaSeq 6000 platform.

Online datasets download and processing

RNA-sequencing data from TCGA-OV cohorts was downloaded using the TCGAbiolinks (v2.16.4). Clinical data for these patients was downloaded using the TCGA clinical data resource. Clinical data and sequencing data of GSE49997, GSE143897, GSE227100, GSE222557, and sequencing data of GSE119273, and GSE131860 were downloaded from the NCBI GEO database. For single-cell RNA-seq data of GSE222557, the cells identified as monocytes and macrophages in the study by Gao et al.(19) were analyzed in our study.

Deconvolution

Deconvolution was performed using CIBERSORTx. Single-cell RNA-sequencing data from PDX undergoing chemotherapy cycles was used to generate a single-cell reference

sample matrix. This consisted of fibroblasts, endothelial cells, macrophages, dendritic cells, and neutrophils. This reference sample matrix was then used to generate a signature matrix file, using the default CIBERSORTx settings. Cell fraction prediction was then performed in relative mode, using the signature matrix and 'S-mode' batch correction.

GSVA (Gene Set Variation Analysis), GSEA (Gene Set Enrichment Analysis), GO (Gene Ontology), and KEGG (Kyoto Encyclopedia of Genes and Genomes) enrichment analysis

GSVA was performed using the GSVA R package (version 1.46.0). 10 gene sets were used to identify macrophage subtype signatures enriched in different macrophage subclusters. The lists of genes utilized for GSVA analysis in single-cell RNA-seq data were provided in Supplementary Table 2. Macrophage_c1 marker genes *(C1qa, C1qb, C1qc, Fcrls*), macrophage_c2 marker genes (*Thbs1*, *Chil3*, *Srgn*, *Arg1*, *Spp1*, *Lgals3*), macrophage_c3 marker genes (*Maf*, *Cx3cr1*, *Ccl12*, *Ccl4*, *Kif2*), macrophage_c4 marker genes (*Rsad2*, *Isg15*, *Ccl5, Irf7*, *Ms4a4c*), macrophage_c5 marker genes (*Ccl8*, *Wfdc17*, *Sepp1, Ctsd*, *Ccl6*), macrophage_c6 marker genes (*Fbln2*, *Col1a2*, *Col3a1, Col1a1*, *Mysm1*) were used to identify signature in macrophage and macrophage incubated with apoptotic cells. By using clusterProfiler (version 4.14.6), GSEA analysis was performed for each cell subpopulation and RAW264.7 was incubated with apoptotic cells or not. KEGG and GO enrichment analysis was performed for DEGs

using clusterProfiler. The results were visualized using SCP, Complexheatmap, and ggplot2.

Cell culture

Human ovarian cancer cell lines OVCAR3 (RRID: CVCL_0465, High-Grade Serous Carcinoma), OVCAR8 (RRID: CVCL_1629, Ovarian High-Grade Serous Carcinoma), SKOV3 (RRID: CVCL_0532, Ovarian Serous Carcinoma), ES-2 (RRID: CVCL_3509, Ovarian Clear Cell Adenocarcinoma), A2780 (RRID: CVCL_0134, Ovarian Endometrioid Adenocarcinoma) were purchased from the China Center for Type Culture Collection (CCTCC, China). OVCAR3 was cultured in the RPMI-1640 (BasalMedia, L210KJ) with 20% fetal bovine serum (Biochannel, BC-SE-FBS01), and OVCAR8 was cultured in the RPMI-1640 with 10% fetal bovine serum. SKOV3, ES-2, and A2780 were cultured in the DMEM/F12 (BasalMedia, L310KJ) with 10% fetal bovine serum. Human myeloid cell line THP-1 (RRID: CVCL_0006) was purchased from the CCTCC and cultured in the RPMI-1640 with 10% fetal bovine serum. All human cell lines were cultured in media with 0.5% penicillin/streptomycin (BasalMedia, S110JV). Mouse macrophage cell line Raw 264.7 (RRID: CVCL_0493) was purchased from CCTCC and cultured in DMEM with 10% fetal bovine serum. Mouse ovarian cancer cell line ID8 (RRID: CVCL_IU14) was purchased from CCTCC and ID8-HM subline was established after cycles in vivo selection(20). All cells were cultured at 37 °C with 5% CO_2. The cell lines we used in the experiments were authenticated via short tandem repeat profiling at Shanghai Biowing Applied

Biotechnology Co., LTD. (China). All cells have undergone mycoplasma testing. All cell experiments are conducted under mycoplasma-free conditions.

Transfection

RNA interference CD44-specific siRNA was purchased from JTSBIO Co., Ltd (Wuhan,China), with target sequences (si-NC CCUCCA CCGAGACCUUAAATT, si-1 GCUGACCUCUGCAAGGCUUTT, and si-2 CCACUGCUUAUGAAGGAAATT) Transfection was performed using Lipofectamine 3000 (Invitrogen) according to the manufacturer's protocol. Knockdown of ODC1 in macrophages was constructed by using the lentivirus expressing short hairpin RNA targeting ODC1 (shNC TTCTCCGAACGTGTCACGT, sh-1 GCCGACGATCTACTATGTGAT, and sh-2 CCTTGTAAACAAGTATCTCAA).

Macrophage preparation

For THP-1 derived macrophage preparation, THP-1 cells were plated in 6 wells-plates at 10^6 cells/ well followed by incubation with 150 ng PMA (S7791, Selleck) in the medium for 24 h. PBMCs were isolated from blood samples for monocyte-derived macrophage preparation. $CD14^+$ monocytes were isolated from PBMCs using the MojoSort™ Human $CD14^+$ Monocytes Isolation Kit (480048, Biolegend) based on the manufacturer's instructions, plated in 6 wells-plates at 10^6 cells/well, and incubated with medium containing 50ng/ml M-CSF (RP01221, Abclonal). Human monocytes were ascertained using flow cytometry and anti-human CD14-PE/Dazzle 594 (398713,

Biolegend, RRID:AB_2890846). All macrophages were ascertained using flow cytometry and anti-human CD68-APC (333809, Biolegend, RRID:AB_10567107) (Figure S4E).

Apoptosis assays

The cancer cells were treated with cisplatin or paclitaxel for 48 hours, and PBS for cisplatin as well as DMSO for paclitaxel were used as solvent control. Then, the cancer cells were stained by 7-AAD and Annexin V-PE by using an Apoptosis Detection Kit (BD, 559763) and the cell apoptotic rate was measured by the flow cytometer. The percentage of apoptotic cells was determined by the percentage of Annexin V-positive cells. Each experiment was performed in at least three biological replicates. FlowJo software (10.4.1) was used for all data analyses.

Quantitative reverse transcription- polymerase chain reaction (qRT-PCR)

Total RNA was extracted from cells using the FreeZol Reagent (R711-01, Vazyme), and reverse transcription was performed with the ABScript III RT Master Mix (RK20429, Abclonal) in accordance with the manufacturer's instructions. qRT-PCR was conducted with the SYBR Green Fast qPCR Mix (RK21203, Abclonal) on a qTOWER3 Real-Time PCR System (analytikjena, Germany). The sequences of the qRT-PCR primers are listed in Supplementary Table 4. Each assay was performed with at least three biological replicates.

Immunohistochemistry (IHC)

Briefly, paraffin slides were dehydrated through a graded alcohol series, followed by antigen retrieval in 0.01 M citrate buffer (pH = 6.0) or tris-EDTA buffer (pH = 9.0), then blocked with 3% H2O2 and blocked with 5% goat serum for 20 min at 37 °C. The slides were then incubated with primary antibodies including anti-CD44 (#37259, Cell Signaling Technology, 1:500), anti-ALDH1A1 (15910-1-AP，Proteintech, 1:200), anti-SOX2 (A0561, Abclonal, 1:200), anti-KI67 (27309-1-AP, Proteintech, 1:2000) and anti-Cleaved caspase-3 (#9664, Cell Signaling Technology, 1:400) at 4 °C overnight. On the following day, the slides were incubated at room temperature with the corresponding biotinylated secondary antibodies and horseradish peroxidase-conjugated streptavidin. The slides were then stained with diaminobenzidine chromogen and hematoxylin. Finally, the slides were dehydrated and mounted, and images were collected and analyzed.

MTT assay

Ovarian cancer cells cultured with macrophage CM were seeded onto 96-well plates at a density of 5,000 cells per well. The cells were then exposed to various concentrations of cisplatin or paclitaxel for 48 h to evaluate the cytotoxicity of cisplatin or paclitaxel to cervical cancer cells. MTT reagent (3-(4,5-Dimethylthiazol-2-yl)-2,5-diphenyltetrazolium bromide, at a final concentration of 10%; Servicebio, Wuhan, China) was then added to each well and incubated further for 4 h at 37°C before DMSO (Biosharp, Wuhan, China) was added to dissolve the MTT formazan product. Absorbance at 570 nm was measured with a microplate reader (MD, i3x, USA), and

cellular survival fractions were calculated by normalizing the optical density of treated wells to that of untreated controls. The dose-response curves were plotted by using GraphPad Prism (version 9.0.0, GraphPad Software, USA). Each experiment was performed with at least three biological replicates.

Establishment of patient-derived organoids (PDOs)

Fresh OC (Ovarian cancer) specimens were surgically resected, minced into small pieces (<1 mm^3), and enzymatically digested to generate single-cell suspensions. These suspensions were subsequently mixed with Matrigel and plated onto 24-well plates. The cells were cultured in Advanced DMEM/F12 medium supplemented with the following components: 1% penicillin-streptomycin, 1× Glutamax (Gibco, 35050061), 1% HEPES (Stemcell, 07200), 1× B27 (Stemcell, 05731), 100 ng/mL Noggin (Peprotech, 120-10C), 50 ng/mL EGF (Peprotech, AF-100-15), 100 ng/mL R-spondin 1 (Peprotech, 120-38), 10 ng/mL FGF-10 (Peprotech, 10026), 10 ng/mL FGF2 (Peprotech, 100-18B), 0.02 μg/mL Wnt3a (R&D Systems, 5036-WN), 0.05 μg/mL human NRG1 (R&D Systems, 5898-NR), 10 mM nicotinamide (Sigma Aldrich, N0636), 1.25 mM N-acetylcysteine (Sigma Aldrich, A9165), 10 nM 17-β-Estradiol (R&D Systems, 2824), 10 μM SB202190 (Sigma Aldrich, S7076), 500 nM A83-01 (Sigma Aldrich, SML0788), and Y-27632 dihydrochloride (Stemcell, 72302). The PDOs were treated with macrophage CM and efferocytotic macrophage CM for 48 h. The Organoids were harvested, embedded in paraffin, sectioned, and subjected to immunohistochemistry (IHC) to assess protein levels.

Western blotting analysis

Total cellular proteins was extracted with RIPA (Beyotime Biotechnology, China) lysis buffer containing proteinase inhibitor cocktail (APExBIO, USA), centrifuged at 12,500×g for 15 min, and quantified via a BCA assay kit (Beyotime Biotechnology, China). Samples were separated by 10% sodium dodecyl sulfate-polyacrylamide gel electrophoresis (SDS-PAGE) and transferred onto polyvinylidene difluoride (PVDF) membranes. The membranes were then blocked with 5% nonfat milk in Tris-buffered saline with Tween 20 (TBST) for 2 h at room temperature and incubated with primary antibodies at 4 °C overnight. After incubation of the peroxidase conjugated secondary antibody for 120 minutes, there was visualization of the antigen-antibody reaction by enhanced chemiluminescence (ECL, HYC0316, HYCEZMBIO) under the ChemiDoc XRS +. Antibodies including anti-GAPDH (A19056, Abclonal, 1:10000), anti-β-actin (AC026, Abclonal, 1:10000), anti-ODC1 (A3898, abclonal, 1:1000), anti-OPN (A21084, abclonal, 1:1000), anti-SOX2 (A0561, Abclonal, 1:200), HRP-conjugated Goat anti-Rabbit IgG (H+L) (AS014, Abclonal, 1:10000) and HRP-conjugated Goat anti-Rabbit IgG (H+L) (AS003, Abclonal, 1:10000) were used in Western blotting.

LC-MS/MS analysis

Sample preparation and extraction

Macrophages were incubated with the apoptotic ovarian cancer cells at a ratio of 1:5 for 24 h followed by ACs removal and incubation for 2 h. The efferocytotic

macrophages and macrophages incubated without Acs were detached for LC-MS/MS analysis. The samples stored at -80 °C refrigerator was thawed on ice. A 500 μL solution (Methanol: Water = 4:1, V/V) containing internal standard was added into the cell sample and vortexed for 3 min. The sample was placed in liquid nitrogen for 5 min and on the dry ice for 5 min, and then thawed on ice and vortexed for 2 min. This freeze-thaw circle was repeated three times in total. The sample was centrifuged at 12000 rpm for 10 min (4°C). A 300 μL of supernatant was collected and placed in -20°C for 30 min. The sample was then centrifuged at 12000 rpm for 3 min (4 °C). A 200 μL aliquots of supernatant were transferred for LC-MS/MS analysis.

T3 UPLC Conditions

The sample extracts were analyzed using an LC-ESI-MS/MS system (UPLC, ExionLC AD, https:// sciex.com.cn/; MS, QTRAP® System, https://sciex.com/). The analytical conditions were as follows, UPLC: column, Waters ACQUITY UPLC HSS T3 C18 (1.8 μm, 2.1 mm*100 mm); column temperature, 40 °C; flow rate, 0.4 mL/min; injection volume, 2μL; solvent system, water (0.1% formic acid): acetonitrile (0.1% formic acid); solvent B gradient program, 5% to 20 % in 2 min, increased to 60 % in the following 3 min, increased to 99 % in 1 min and held for 1.5 min, then come back to 5 % within 0.1 min, held for 2.4 min.

ESI-QTRAP-MS/MS

LIT and triple quadrupole (QQQ) scans were acquired on a triple quadrupole-linear ion

trap mass spec- trometer (QTRAP), QTRAP® LC-MS/MS System, equipped with an ESI Turbo Ion-Spray interface, oper- ating in positive and negative ion mode and controlled by Analyst 1.6.3 software (Sciex). The ESI source operation parameters were as follows: source temperature 500 °C; ion spray voltage (IS) 5500 V (positive), 4500 V (negative); ion source gas I (GSI), gas II (GSII), curtain gas (CUR) were set at 55, 60, and 25.0 psi, respectively; the collision gas (CAD) was high. Instrument tuning and mass calibration were performed with 10 and 100 μmol/L polypropylene glycol solutions in QQQ and LIT modes, respectively. A specific set of MRM transitions were monitored for each period according to the metabolites eluted within this period.

PCA

Unsupervised PCA (principal component analysis) was performed by statistics function prcomp within R (www.r-project.org). The data was unit variance scaled before unsupervised PCA.

Differential metabolites selected

For two-group analysis, differential metabolites were determined by VIP (VIP > 1) and P-value (P-value < 0.05, Student's t test). VIP values were extracted from OPLS-DA result, which also contain score plots and permutation plots, was generated using R package MetaboAnalystR. The data was log transform (log2) and mean centering before OPLS-DA. In order to avoid overfitting, a permutation test (200 permutations) was performed.

KEGG annotation and enrichment analysis of metabolites

Identified metabolites were annotated using KEGG Compound database (http://www.kegg.jp/kegg/ compound/), annotated metabolites were then mapped to KEGG Pathway database (http://www.kegg.jp/ kegg/pathway.html). Pathways with significantly regulated metabolites mapped to were then fed into MSEA (metabolite sets enrichment analysis), their significance was determined by hypergeometric test's p-values.

Colony formation assays

Cervical cancer cells were seeded onto 6-well plates at 1,000 cells per well and incubated with fresh medium for 1 weeks, the colonies were fixed with 4% paraformaldehyde for 30 min, stained with 0.5% crystal violet for 30 min, and photographed.

Animal experiments

All animal experiments were conducted in accordance with the guidelines approved by the Institutional Animal Care and Use Committee of Tongji Medical College, Huazhong University of Science and Technology. All protocols were approved by the Institutional Animal Care and Use Committee of the Tongji Medical College, Huazhong University of Science and Technology. All mice used in this study were maintained under pathogen-free conditions at the animal experiment center at 22 °C and 60% humidity on a 14 hour-light and dark cycle. Syngeneic models of ID8-HM cells were established by subcutaneous, intraperitoneal, or orthotopic transplantation. For the ID8-HM cell injection assays, ID8-HM cells (2×10^6) were directly injected subcutaneously or

intraperitoneally into 5-week-old female C57BL/6J mice. For the SKOV3 cell injection assays, SKOV3 cells (2×10^6) were directly injected subcutaneously into 5-week-old female BALB/c Nude mice. For ID8-HM orthotopic transplantation assays, tumor tissue blocks of subcutaneous tumors derived from ID8-HM cells were transplanted into the ovarian bursae of C57BL/6J mice to establish orthotopic tumors. For intraperitoneal injection tumor experiments, mice were randomly assigned to groups on the 3rd day post-injection and received treatment. For orthotopic transplantation tumor experiments, mice were randomly assigned to groups on the 14th day ≠ post-transplantation and received treatment. Intraperitoneal and orthotopic tumors were monitored for tumor growth using an *in vivo* imaging system (IVIS, Lumina II, Caliper Life Science, USA). The radiance value was measured using Living Image® software (version 4.3.1, Caliper Life Science, USA). For subcutaneous injection tumor experiments, when the xenograft reached approximately 50 mm^3, the mice were randomly assigned into groups and received treatment. For PDX experiments, when the xenograft reached approximately 50 mm^3, the mice were randomly assigned to groups and received treatment. Tumor size was measured every 2 days, and the volume was calculated using the following formula: Volume (mm^3) = (length $\times$ width^2)/2. The following substances were used in the treatment of mice: UNC2250 (10 mg/kg, oral gavage, once daily), paclitaxel (10 mg/kg, intraperitoneal administration, every three days), cisplatin (3 mg/kg, intraperitoneal administration, every three days), and DFMO (1% in drinking water).

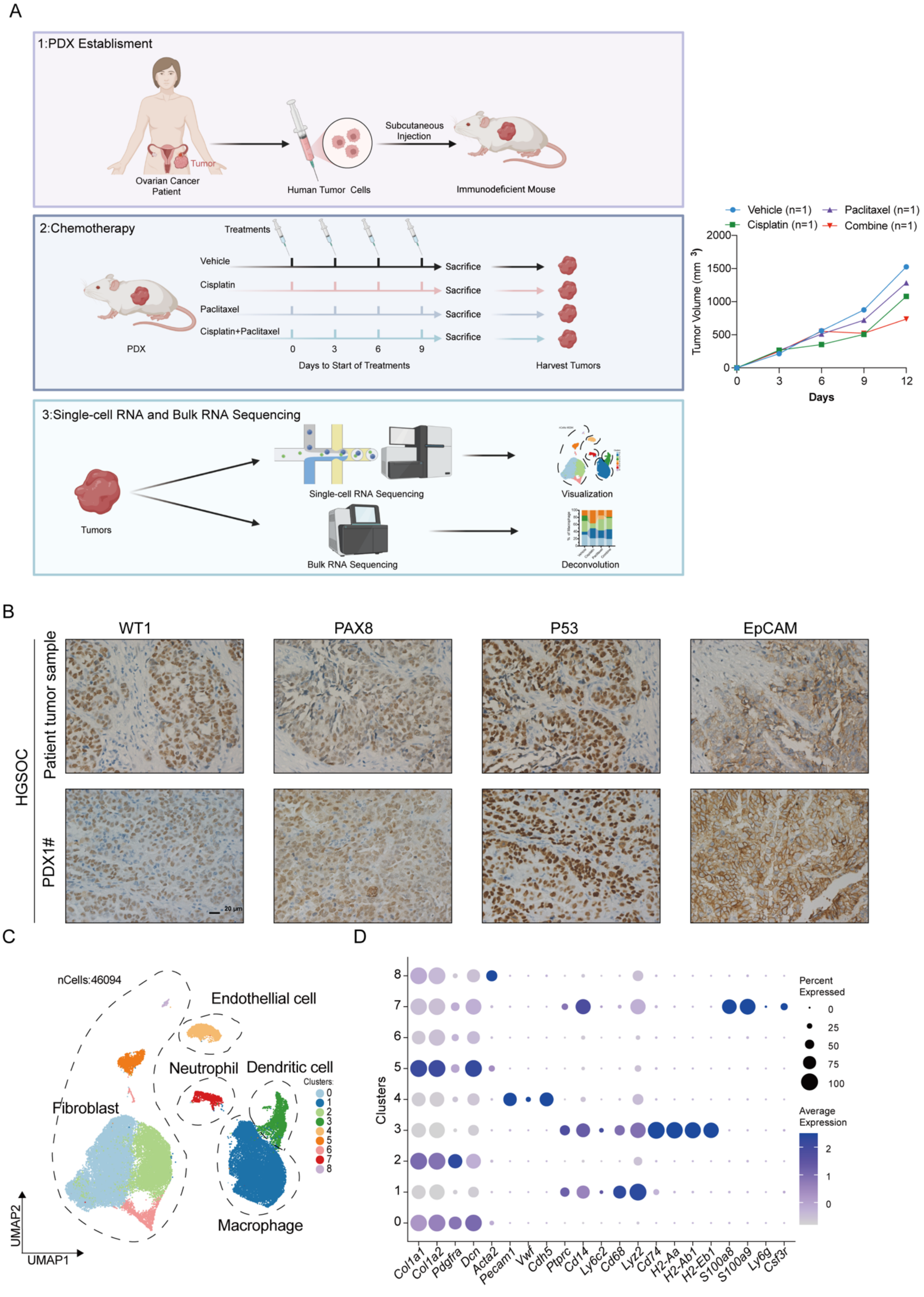

Figure S1: Single-cell sequencing analysis in PDX undergoing cycles of

chemotherapy.

A, Flowchart of PDX (Patient-derived xenograft) treatment, RNA sequencing, and analysis (Flowchart was created by BioRendr.com).

B, Identification of PDX pathological characters by IHC (Immunohistochemistry).

C, Uniform Manifold Approximation and Projection (UMAP) of all cells from the scRNA-seq (Single-cell sequencing) of PDX. Points were colored by different clusters.

D, Dot plot showing expression levels of Fibroblast markers (*Col1a1*, *Col1a2*, *Pdgfra*, *Dcn*, *Acta2*), endothelial cell markers (*Peacam1*, *Vwf*, *Cdh5*), immune cell marker (*Ptprc1*), macrophage markers (*Cd14*, *Ly6c2*, *CD68*, *Lyz2*), Dendritic cell markers (*Cd74*, *H2-Aa*, *H2-Ab1*, *H2-Eb1*), Neutrophil (*S100a8*, *S100a9*, *Ly6g*, *Csf3r*) in different cell clusters.

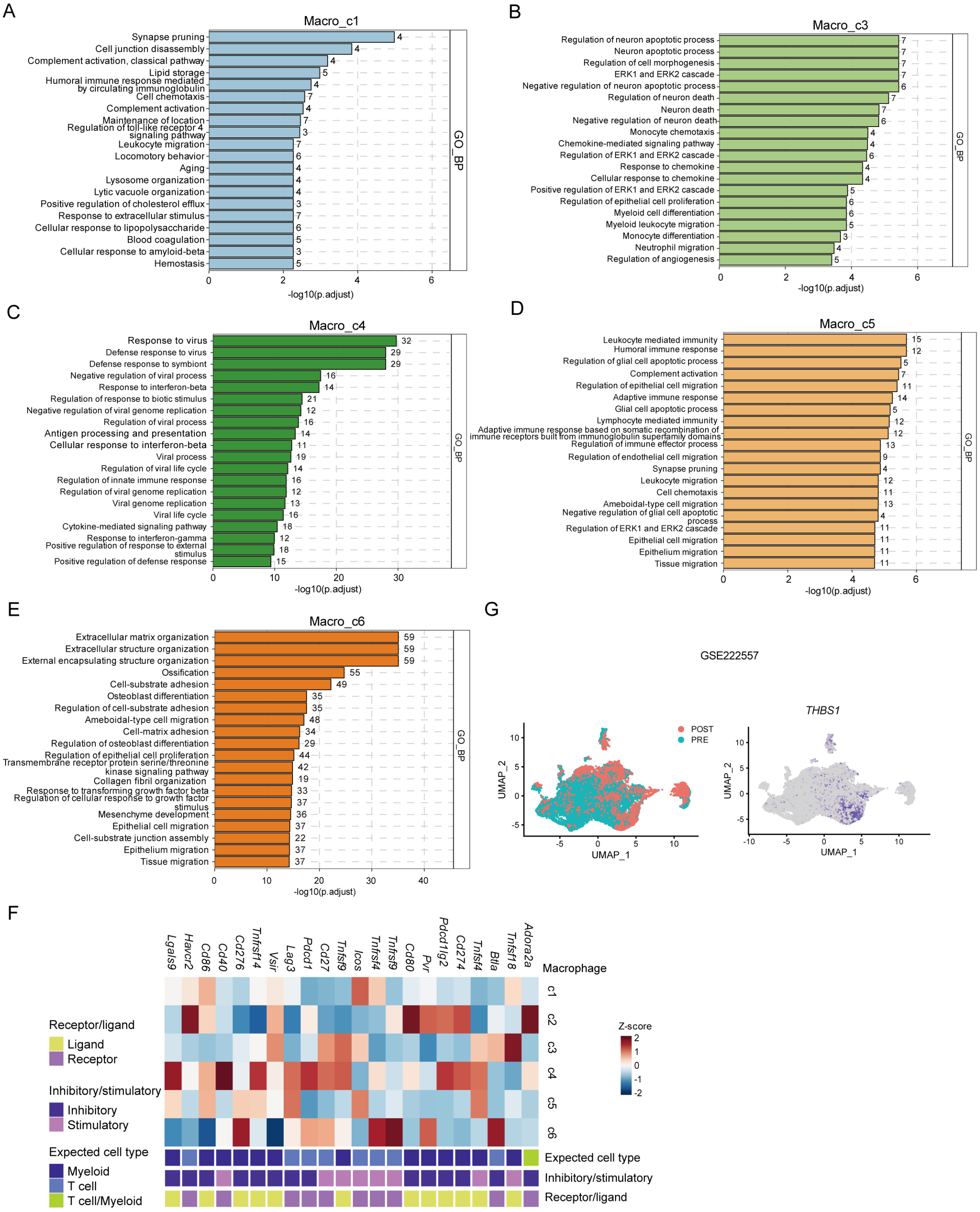

Figure S2: Enrichment analysis in macrophage clusters

A-E, GO:BP enrichment analysis in Macro_C1, and C3-C6 based on their DEGs (differential expression genes).

F, Heatmap showing inhibitory/stimulatory receptors and ligands expression levels among macrophage clusters.

G, UMAP of myeloid cells in GSE222557 showing pre- and post-chemotherapy characteristics and THBS1 expression levels.

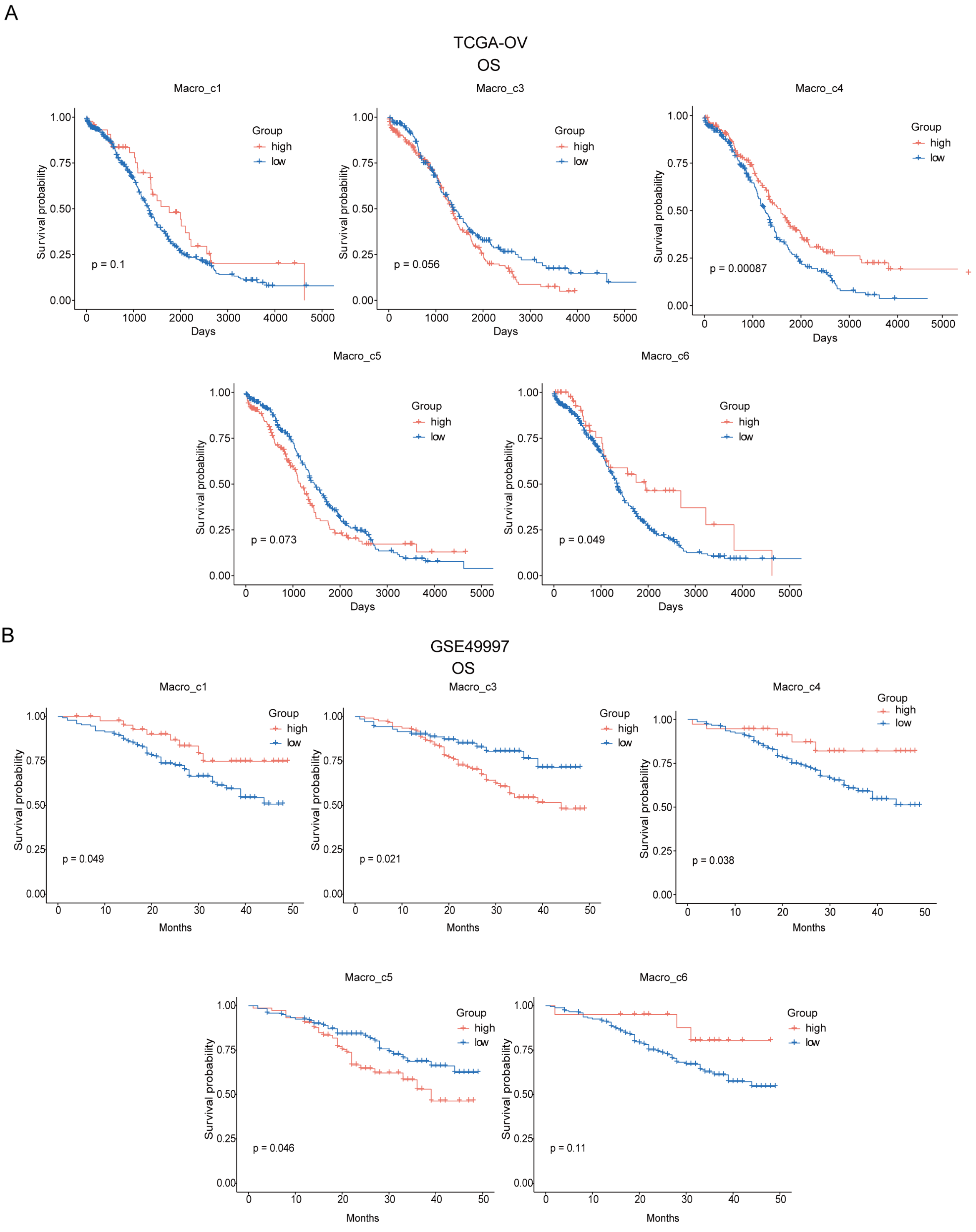

Figure S3: Survival analyses in TCGA-OV and GSE49997

A, Kaplan-Meier analysis of overall survival (OS) for ovarian cancer patients in TCGA-OV between macro_c1, c3-c6 high ratio group and their low ratio group. Statistical significance was tested using log-rank (Mantel-Cox) test.

B, Kaplan-Meier analysis of overall survival (OS) for ovarian cancer patients in GSE49997 between macro_c1, c3-c6 high ratio group and their low ratio group. Statistical significance was tested using log-rank (Mantel-Cox) test.

For all survival analyses, patients were divided into low-ratio and high-ratio groups based on the optimal cut-off value calculated using the R package survminer (versionz0.5

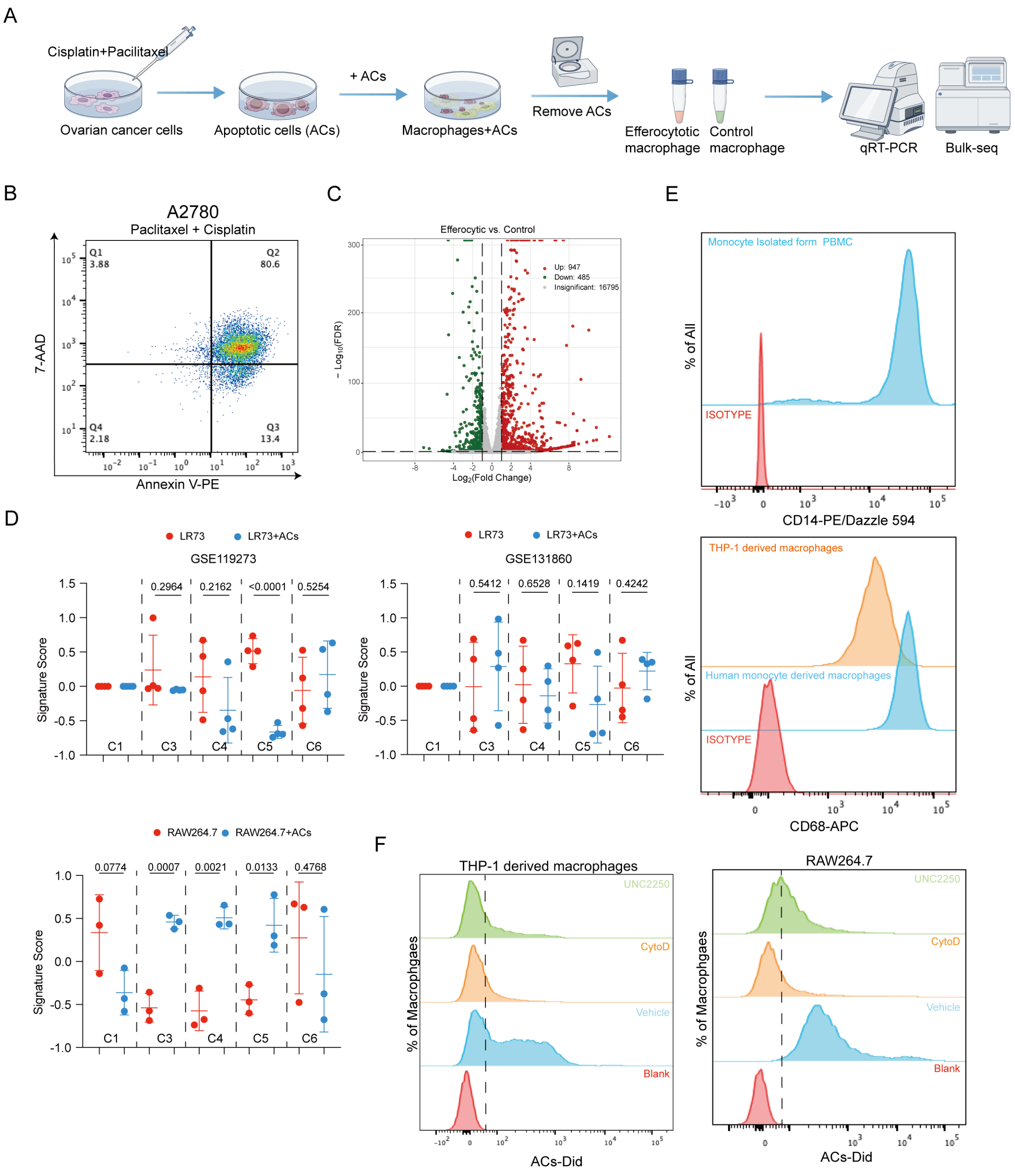

Figure S4: Efferocytosis assays *in vitro*

A, Flowchart of Efferocytosis assays *in vitro*.

B, Representative flow cytometry analysis images of apoptosis assay of A2780 treated with 50 μM cisplatin and 50 nM paclitaxel.

C, The volcano plot of genes in RAW264.7 cells (n=3) and RAW264.7 cells + ACs (n=3).

D, Macrophage c1, c3-c6 signature scores between macrophages and macrophages + ACs.

E, TOP, CD14 expression levels of monocytes isolated from PBMC were ascertained by using flow cytometry. Bottom, CD68 expression levels of THP-1 and monocyte-derived macrophages were ascertained by using flow cytometry.

F, Validation of UNC2250 and CytoD efficacy of inhibiting efferocytosis.

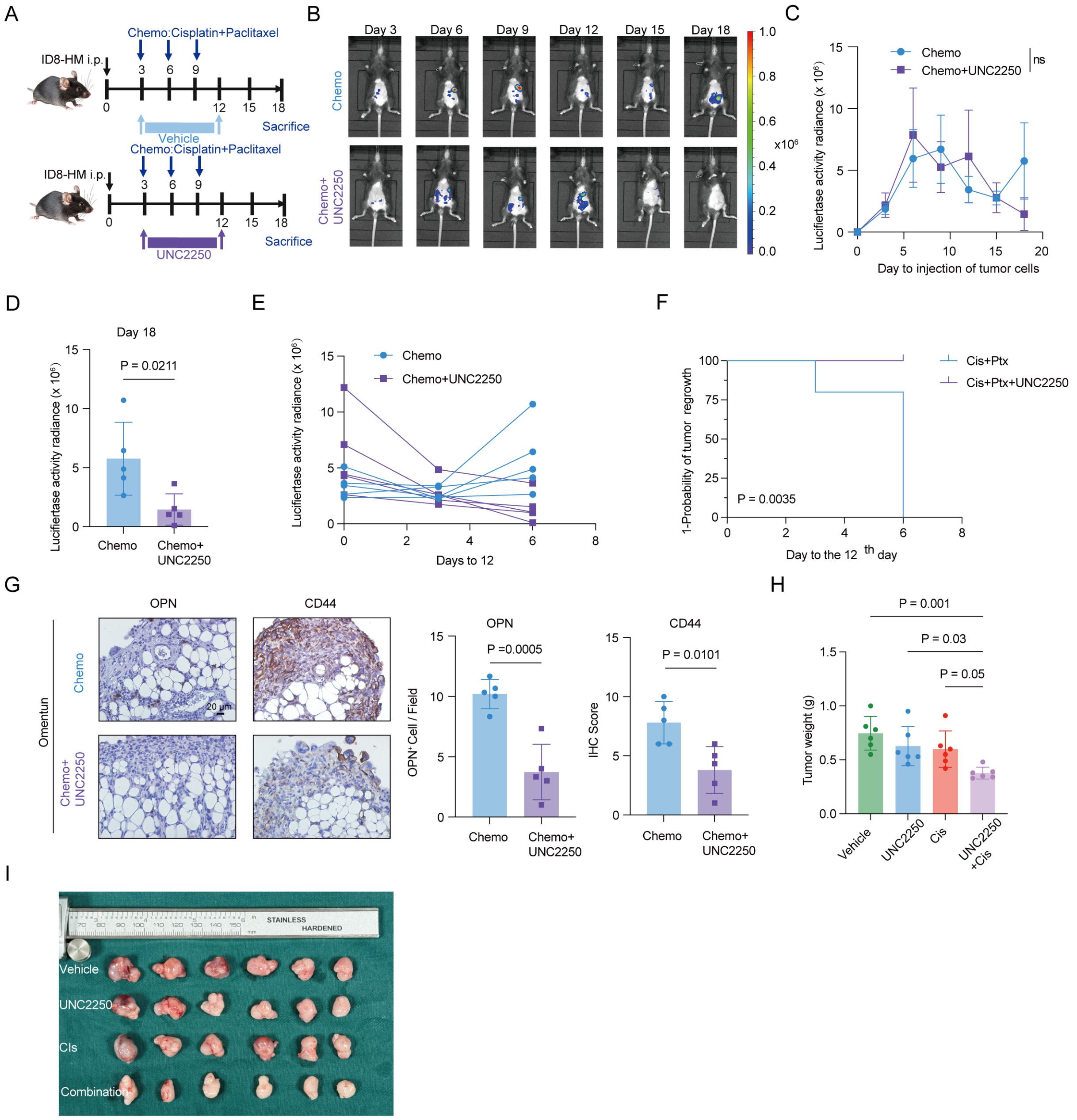

Figure S5: UNC2250 inhibits tumor regrowth post-chemotherapy,

A, Treatment of ID8-HM tumor-bearing mice using cisplatin and paclitaxel combined with UN2250 or vehicle. ID8-HM cells were intraperitoneally injected into C57BL/6J mice. Treatment of UNC2250 or vehicle was initiated on the 3rd day after the injection and sustained for a duration of 9 days. Treatment with cisplatin and paclitaxel was initiated on the 3rd day after the injection of tumor cells, then injected every 3 days for a duration of 9 days.

B, Representative images of the intraperitoneal tumor xenografts treated with cisplatin and paclitaxel combined with UN2250 or vehicle. Tumor burden was assessed by performing in vivo imaging using a live - imaging system on a schedule of every 3 days.

C, Tumor growth curve of mice treated with cisplatin and paclitaxel combined with UN2250 (n=5) or vehicle (n=5). Statistical significance was tested using two-way ANOVA. ns, not significant.

D, Bar graph showing the tumor burden of **A** at the end of the experiment. Statistical significance was tested using Student's t-test.

E, Tumor growth curve of mice in **B** after the 12th day.

F, Kaplan-Meier analysis of tumor regrowth in **B**. Statistical significance was tested using the log-rank (Mantel-Cox) test.

G, Representative IHC images of OPN and CD44 staining in the Omentum tumors treated with chemotherapy or chemotherapy plus UNC2250. Bar graphs showing CD44, and the numbers of OPN^+ cells in the Omentum tumors. Statistical significance was tested using Student's t-test.

H, Representative images of tumors from mice with SKOV3 treated with vehicle, cisplatin, UNC2250, or cisplatin combined with UNC2250.

I, Tumor weights of **H.** Statistical significance was assessed using one-way ANOVA and Dunnett's multiple comparisons test.

All data are presented as Mean ± SD

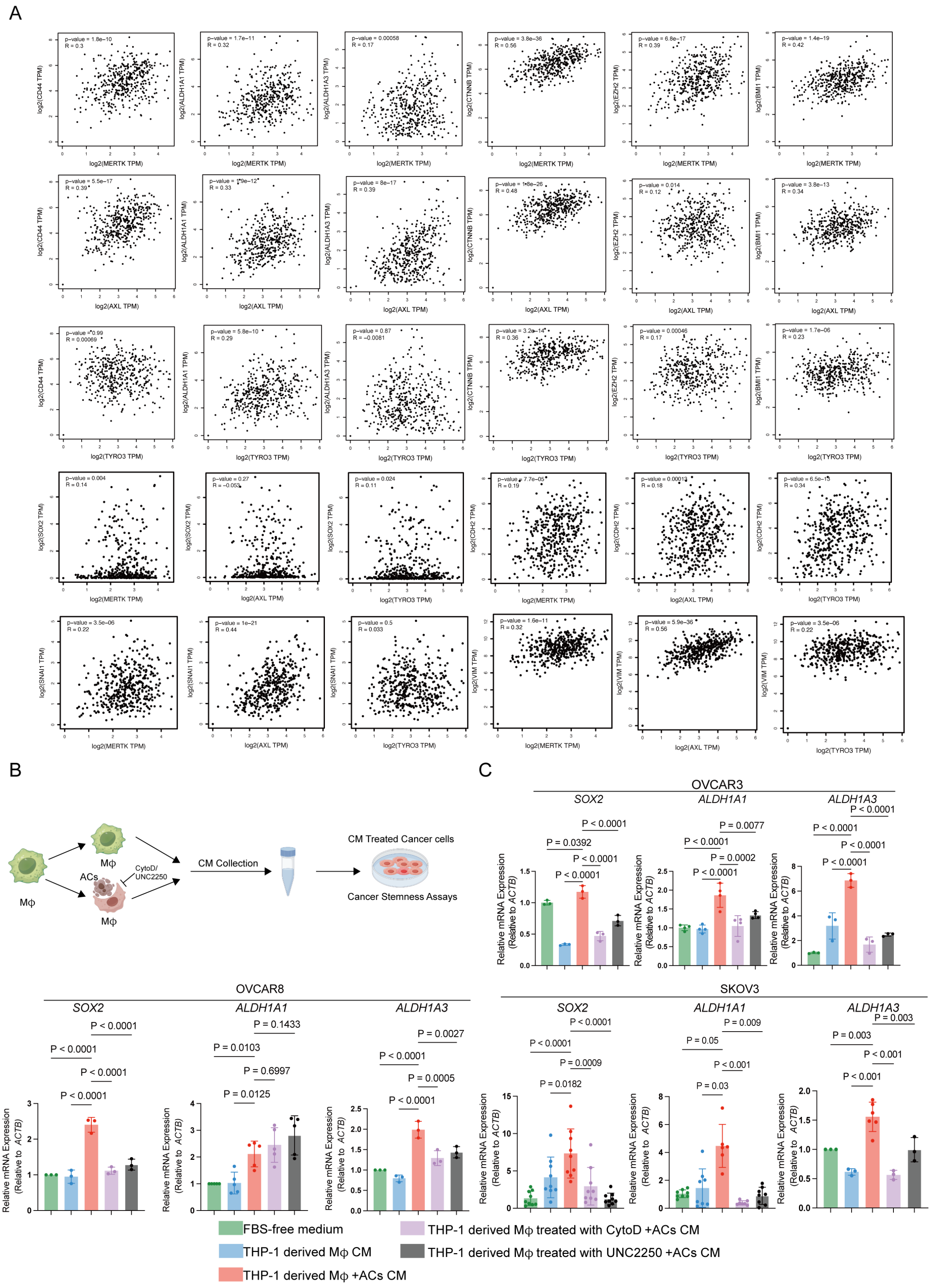

Figure S6: Efferocytosis is associated with cancer stemness

A, Correlation analysis of efferocytosis-associated genes (*MERTK*, *AXL*, *TYRO3*) and Cancer stemness-associated genes (*CD44*, *ALDH1A1*, *ALDH1A3*, *CTNNB*, *EZH2*, *BMI1*, *SOX2*, *CDH2*, *SNAI1*, *VIM*) in TCGA-OV. Statistical significance was tested using Spearman correlation analysis.

B, Flowchart of preparation of the conditioned mediums.

C, Bar graphs showing the relative mRNA expression (relative to *ACTB*) of *SOX2*, *ALDH1A1*, *ALDH1A3* of OVCAR8, OVACR3, and SKOV3 treated with FBS-free medium, and THP-1 derived Mφ CM, THP-1 derived Mφ +ACs CM, THP-1 derived Mφ treated with CytoD +ACs CM, THP-1 derived Mφ treated with UNC2250 +ACs CM separately for 48 hours by qRT-PCR. Statistical significance was tested using one-way ANOVA and Dunnett's multiple comparisons test.

All data are presented as Mean ± SD

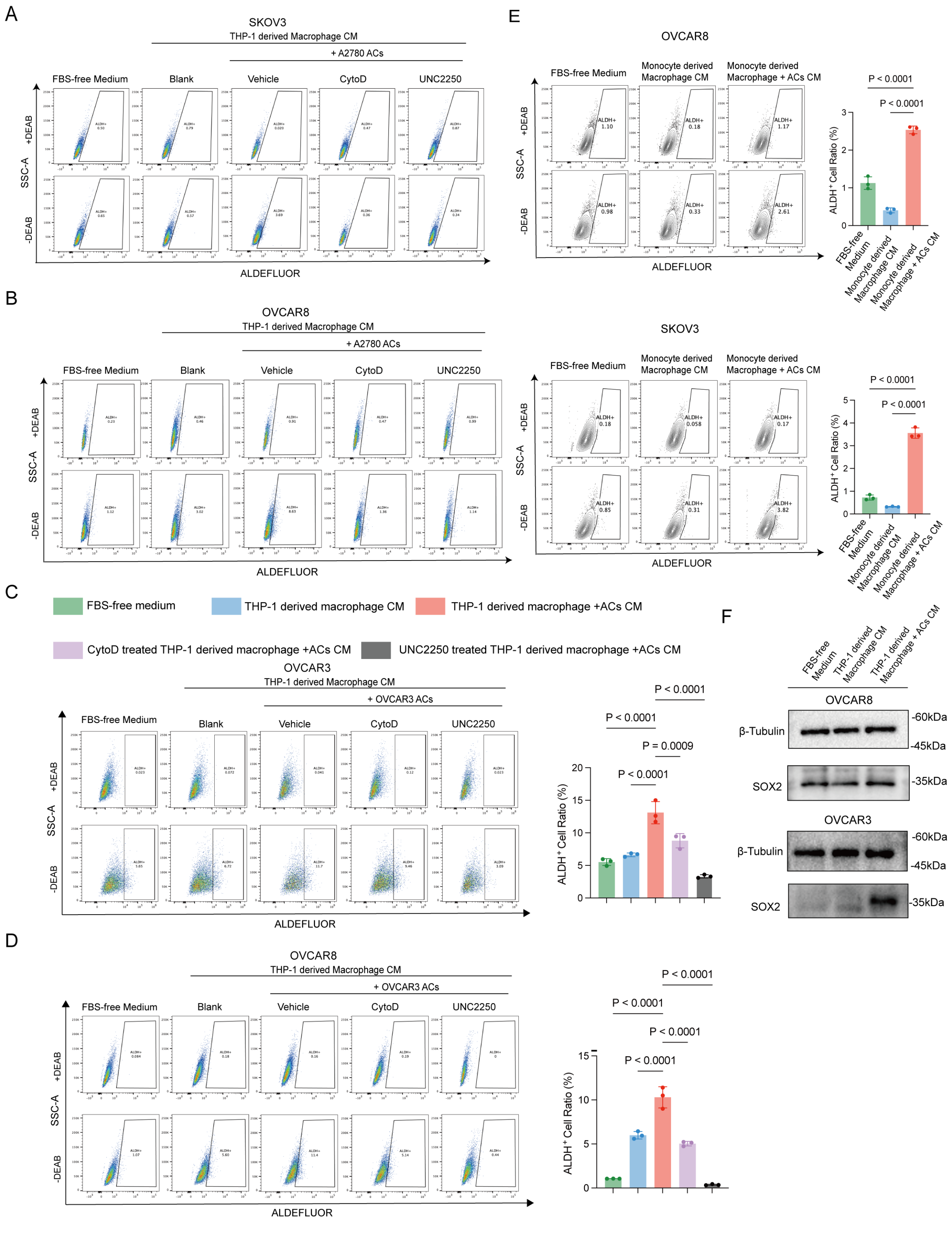

Figure S7: Efferocytotic macrophage CM expands ALDH$^+$ OC cell ratio

A, Representative flow cytometry images of ALDEFLUOR assays in SKOV3 treated with FBS-free medium, THP-1 derived Mφ CM, THP-1 derived Mφ +A2780 ACs CM, THP-1 derived Mφ treated with CytoD +A2780 ACs CM, THP-1 derived Mφ treated

with UNC2250 +A2780 ACs CM separately for 48 hours. The ALDH$^+$ gate was identified by the DEAB group.

B, Representative flow cytometry images of ALDEFLUOR assays in OVCAR8 treated with FBS-free medium, THP-1 derived Mφ CM, THP-1 derived Mφ +A2780 ACs CM, THP-1 derived Mφ treated with CytoD +A2780 ACs CM, THP-1 derived Mφ treated with UNC2250 +A2780 ACs CM separately for 48 hours. The ALDH$^+$ gate was identified by the DEAB group.

C, Representative flow cytometry images of ALDEFLUOR assays in OVCAR3 treated with FBS-free medium, THP-1 derived Mφ CM, THP-1 derived Mφ +OVCAR3 ACs CM, THP-1 derived Mφ treated with CytoD + OVCAR3 ACs CM, THP-1 derived Mφ treated with UNC2250 + OVCAR3 ACs CM separately for 48 hours. The ALDH$^+$ gate was identified by the DEAB group. The bar graphs showed the ALDH$^+$ cell ratio in OVCAR3 treated with different mediums. Statistical significance was tested using one-way ANOVA and Dunnett's multiple comparisons test.

D, Representative flow cytometry images of ALDEFLUOR assays in OVCAR8 treated with FBS-free medium, THP-1 derived Mφ CM, THP-1 derived Mφ +OVCAR3 ACs CM, THP-1 derived Mφ treated with CytoD + OVCAR3 ACs CM, THP-1 derived Mφ treated with UNC2250 + OVCAR3 ACs CM separately for 48 hours. The ALDH$^+$ gate was identified by the DEAB group. The bar graphs showed the ALDH$^+$ cell ratio in OVCAR8 treated with different mediums. Statistical significance was tested using one-way ANOVA and Dunnett's multiple comparisons test.

E, Representative flow cytometry images of ALDEFLUOR assays in OVCAR8 and SKOV3 treated with FBS-free medium, monocyte-derived Mφ CM, monocyte-derived Mφ +A2780 ACs CM separately for 48 hours. The ALDH+ gate was identified by the DEAB group. The bar graphs showed the ALDH+ cell ratio in OVCAR8 and SKOV3 treated with different mediums. Statistical significance was tested using one-way ANOVA and Dunnett's multiple comparisons test. All data are presented as Mean ± SD.

F, Representative western blotting images of SOX2 levels in OVCAR8 and OVCAR3 cells, treated with FBS-free medium, THP-1 derived Mφ CM, THP-1 derived Mφ +A2780 ACs CM

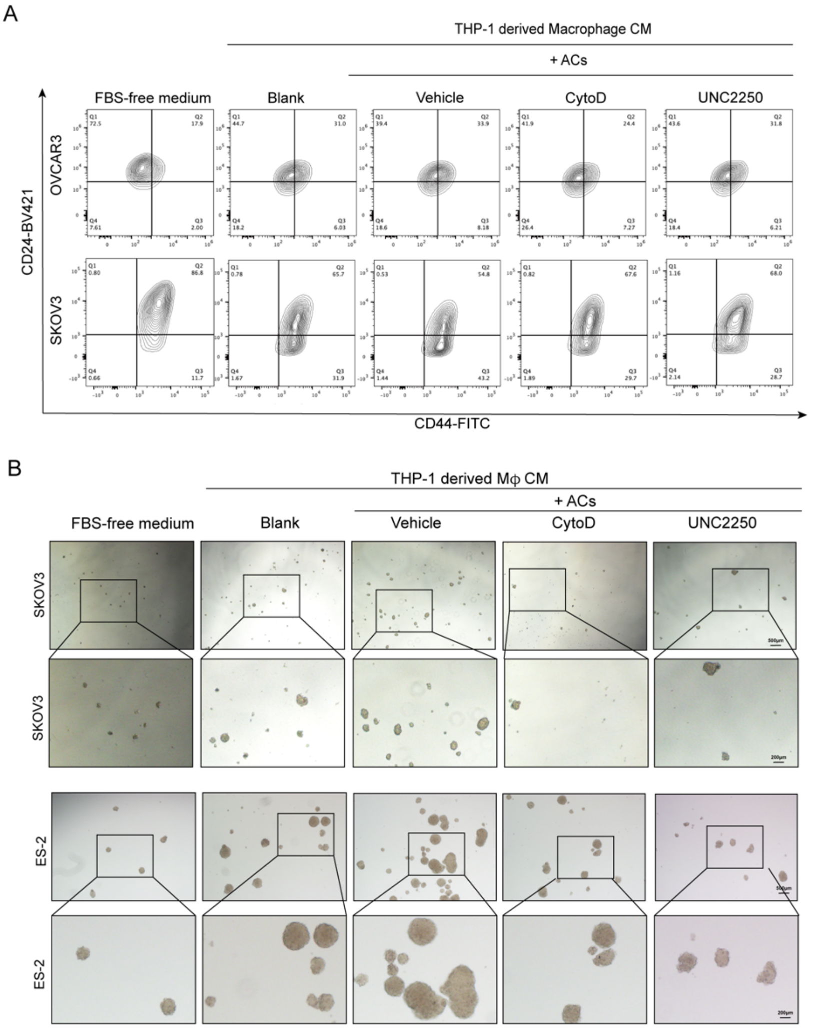

Figure S8: Efferocytotic macrophage CM expands CD24$^-$ CD44$^+$ OC cell ratio and promotes sphere formation

A, Representative flow cytometry images of CD24 and CD44 in OVCAR3 and SKOV3 treated with FBS-free medium, THP-1 derived Mφ CM, THP-1 derived Mφ +ACs CM, THP-1 derived Mφ treated with CytoD +ACs CM, THP-1 derived Mφ treated with UNC2250 +ACs CM separately for 48 hours.

B, Representative images of sphere formation assays in SKOV3 and ES-2 treated with FBS-free medium, THP-1 derived Mφ CM, THP-1 derived Mφ +ACs CM, THP-1

derived Mφ treated with CytoD +ACs CM, THP-1 derived Mφ treated with UNC2250 +ACs CM separately for 48 hours.

A

ELDA of SKOV3

Number of cells	Number of spheres	
	THP-1 derived Mφ CM	THP-1 derived Mφ +ACs CM
100	8/8	8/8
50	8/8	8/8
10	0/8	4/8
5	0/8	3/8
CSC Frequency	29.7	11.9
P.value	0.0478	

ELDA of OVCAR8

Number of cells	Number of spheres	
	THP-1 derived Mφ CM	THP-1 derived Mφ +ACs CM
100	9/10	10/10
50	10/10	10/10
10	6/10	8/10
1	0/11	4/11
CSC Frequency	22.07	5.14
P.value	0.000657	

B

ELDA of SKOV3

Number of cells	Number of spheres	
	Monocyte derived Mφ CM	Monocyte derived Mφ +ACs CM
100	10/11	11/11
50	9/11	11/11
10	1/11	4/11
5	0/11	0/11
CSC Frequency	43.2	20.5
P.value	0.0457	

ELDA of OVCAR8

Number of cells	Number of spheres	
	Monocyte derived Mφ CM	Monocyte derived Mφ +ACs CM
100	9/11	11/11
50	11/11	11/11
10	11/11	10/11
1	0/11	4/11
CSC Frequency	19.26	3.37
P.value	0.0000331	

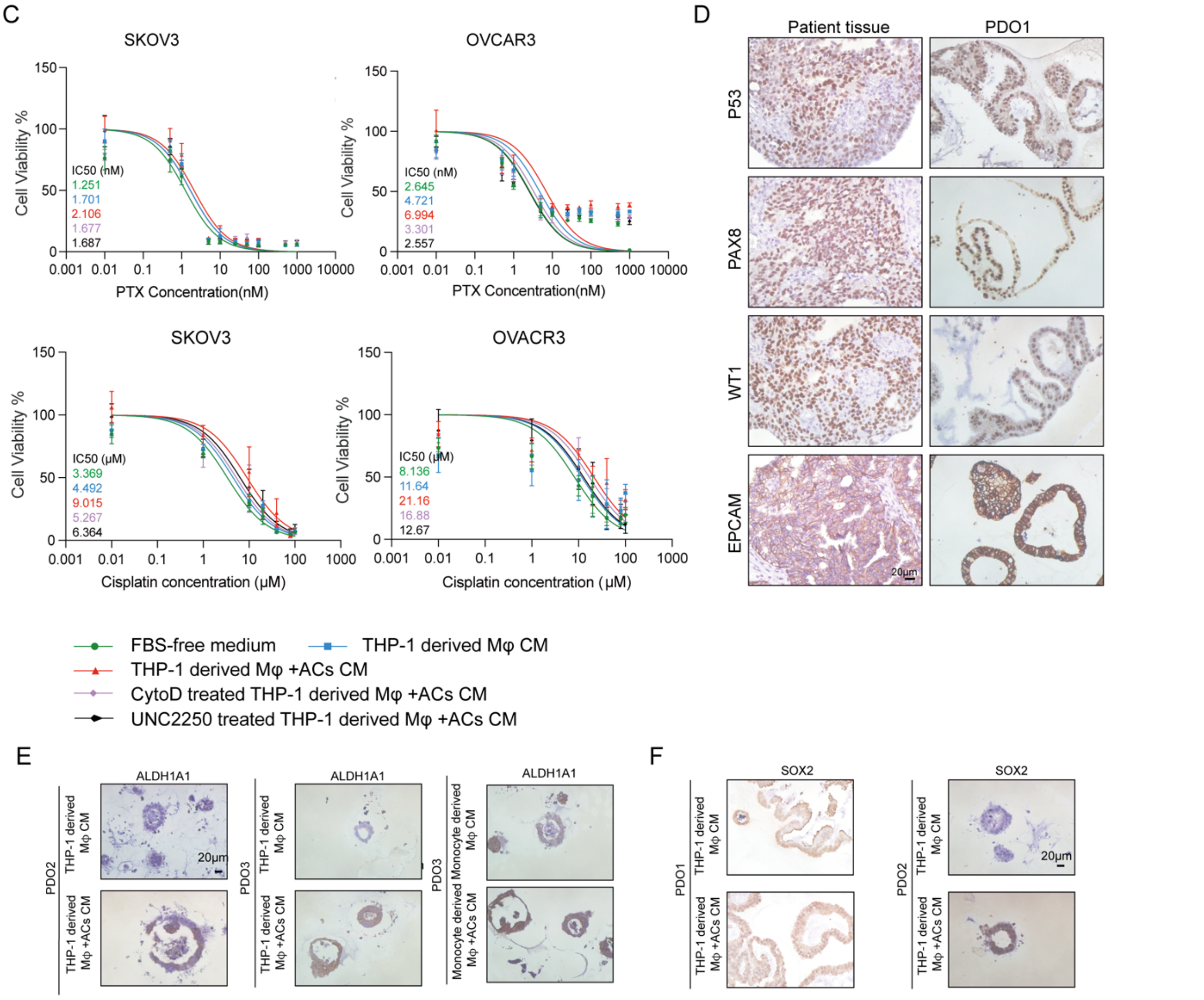

Figure S9: Efferocytotic macrophage CM confers OC cell cancer stemness and chemoresistance

A, Statistics tables of ELDA experiments in OVCAR8 and SKOV3 treated with THP-1 derived Mφ CM and THP-1 derived Mφ +ACs CM.

B, Statistics tables of ELDA experiments in OVCAR8 and SKOV3 treated with monocyte-derived Mφ CM, monocyte-derived Mφ + ACs CM separately.

C, MTT assays showing OC cell viability treated with various concentrations of cisplatin and Paclitaxel. IC_{50} was calculated by using GraphPad.

D, Identification of PDO pathological characters by IHC.

E, Representative IHC images of ALDH1A1 levels in POD treated with Mφ CM and Mφ +ACs CM for 48 hours.

F, Representative IHC images of SOX2 levels in POD treated with Mφ CM and Mφ +ACs CM for 48 hours.

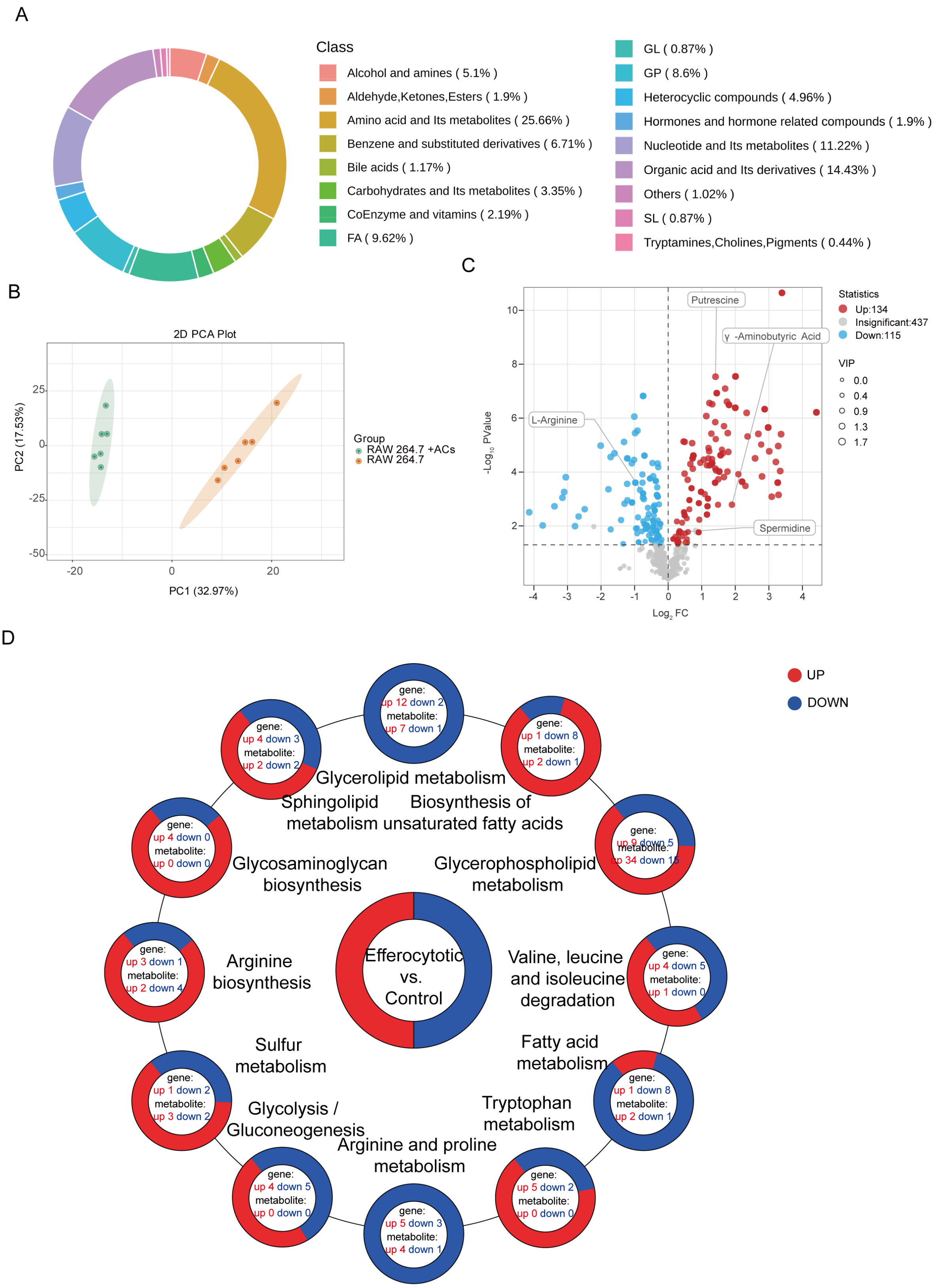

A
Class
Alcohol and amines (5.1%)
Aldehyde,Ketones,Esters (1.9%)
Amino acid and Its metabolites (25.66%)
Benzene and substituted derivatives (6.71%)
Bile acids (1.17%)
Carbohydrates and Its metabolites (3.35%)
CoEnzyme and vitamins (2.19%)
FA (9.62%)
GL (0.87%)
GP (8.6%)
Heterocyclic compounds (4.96%)
Hormones and hormone related compounds (1.9%)
Nucleotide and Its metabolites (11.22%)
Organic acid and Its derivatives (14.43%)
Others (1.02%)
SL (0.87%)
Tryptamines,Cholines,Pigments (0.44%)
B
2D PCA Plot
PC2 (17.53%)
PC1 (32.97%)
Group
RAW 264.7 +ACs
RAW 264.7
C
Putrescine
γ -Aminobutyric Acid
L-Arginine
Spermidine
Statistics
Up:134
Insignificant:437
Down:115
VIP
0.0
0.4
0.9
1.3
1.7
$-Log_{10}$ PValue
Log_2 FC
D
UP
DOWN
Efferocytotic vs. Control
Glycerolipid metabolism
gene: up 12 down 2 metabolite: up 7 down 1
Biosynthesis of unsaturated fatty acids
gene: up 1 down 8 metabolite: up 2 down 1
Glycerophospholipid metabolism
gene: up 9 down 5 metabolite: up 34 down 15
Valine, leucine and isoleucine degradation
gene: up 4 down 5 metabolite: up 1 down 0
Fatty acid metabolism
gene: up 1 down 8 metabolite: up 2 down 1
Tryptophan metabolism
gene: up 5 down 2 metabolite: up 0 down 0
Arginine and proline metabolism
gene: up 5 down 3 metabolite: up 4 down 1
Glycolysis / Gluconeogenesis
gene: up 4 down 5 metabolite: up 0 down 0
Sulfur metabolism
gene: up 1 down 2 metabolite: up 3 down 2
Arginine biosynthesis
gene: up 3 down 1 metabolite: up 2 down 4
Glycosaminoglycan biosynthesis
gene: up 4 down 0 metabolite: up 0 down 0
Sphingolipid metabolism
gene: up 4 down 3 metabolite: up 2 down 2

Figure S10: Efferocytosis drives metabolism reprogramming in macrophage

A, Circle diagram showing the metabolic classes in the metabolome of RAW 264.7 and RAW 264.7 + ACs.

B, PCA plot of metabolome in RAW 264.7 (n=6) and RAW 264.7 + ACs (n=6).

C, Volcano plot of metabolome in RAW 264.7 (n=6) and RAW 264.7 + ACs (n=6).

D, Diagram showing the transcriptionally enriched metabolic pathway (p-value <0.05) and its differential genes and metabolites.

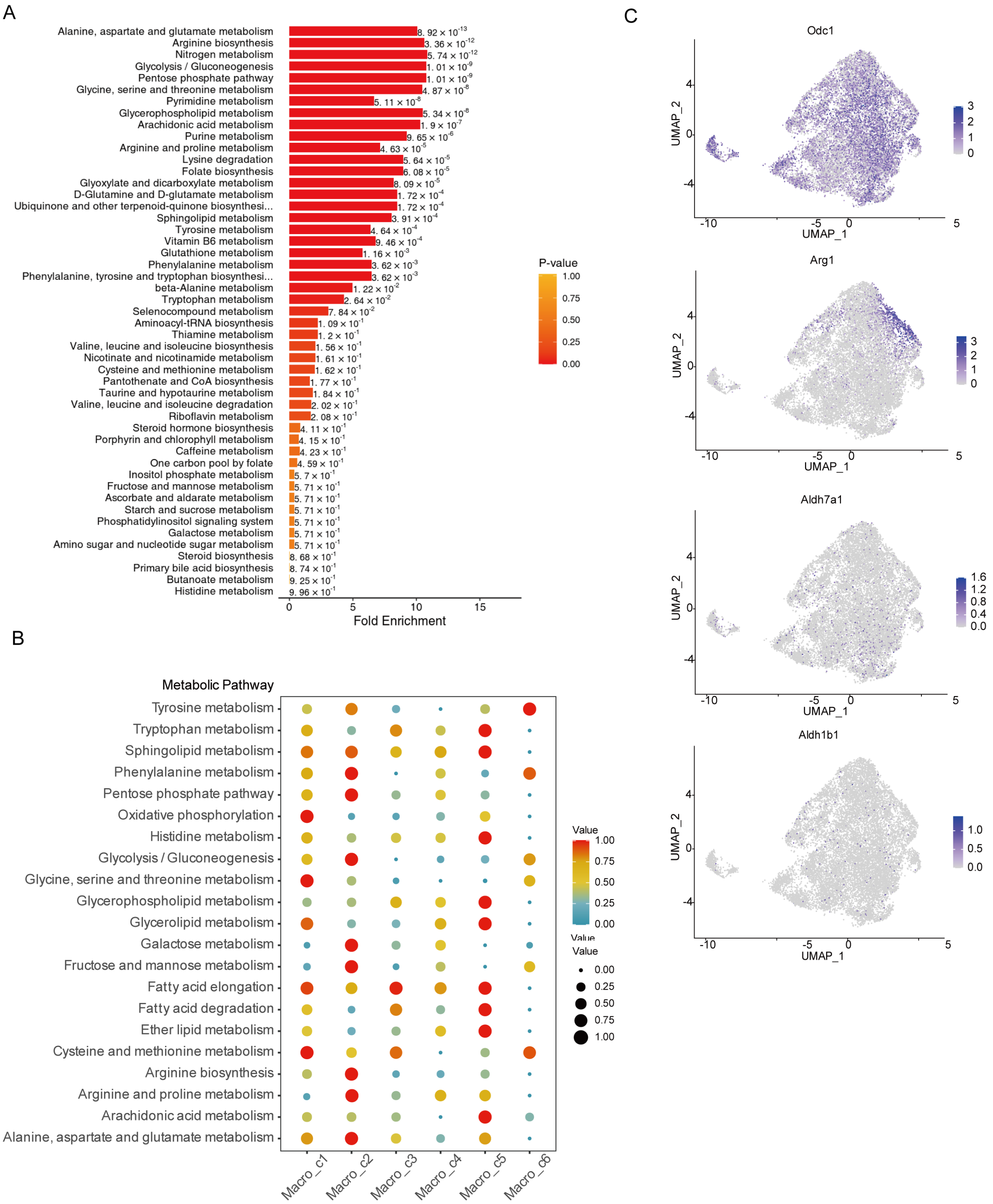

Figure S11: Efferocytosis drives metabolism reprogramming in macrophage

A, Diagram showing the MSEA results of the metabolome in RAW264.7 and RAW264.7+ ACs

B, Dot plot showing the scMetabolism results of the Metabolic Pathway in macro_c1-c6.

C, Feature plot showing the expression of Odc1, Arg1, Aldh7a1 and Aldh1b1 in macrophages.

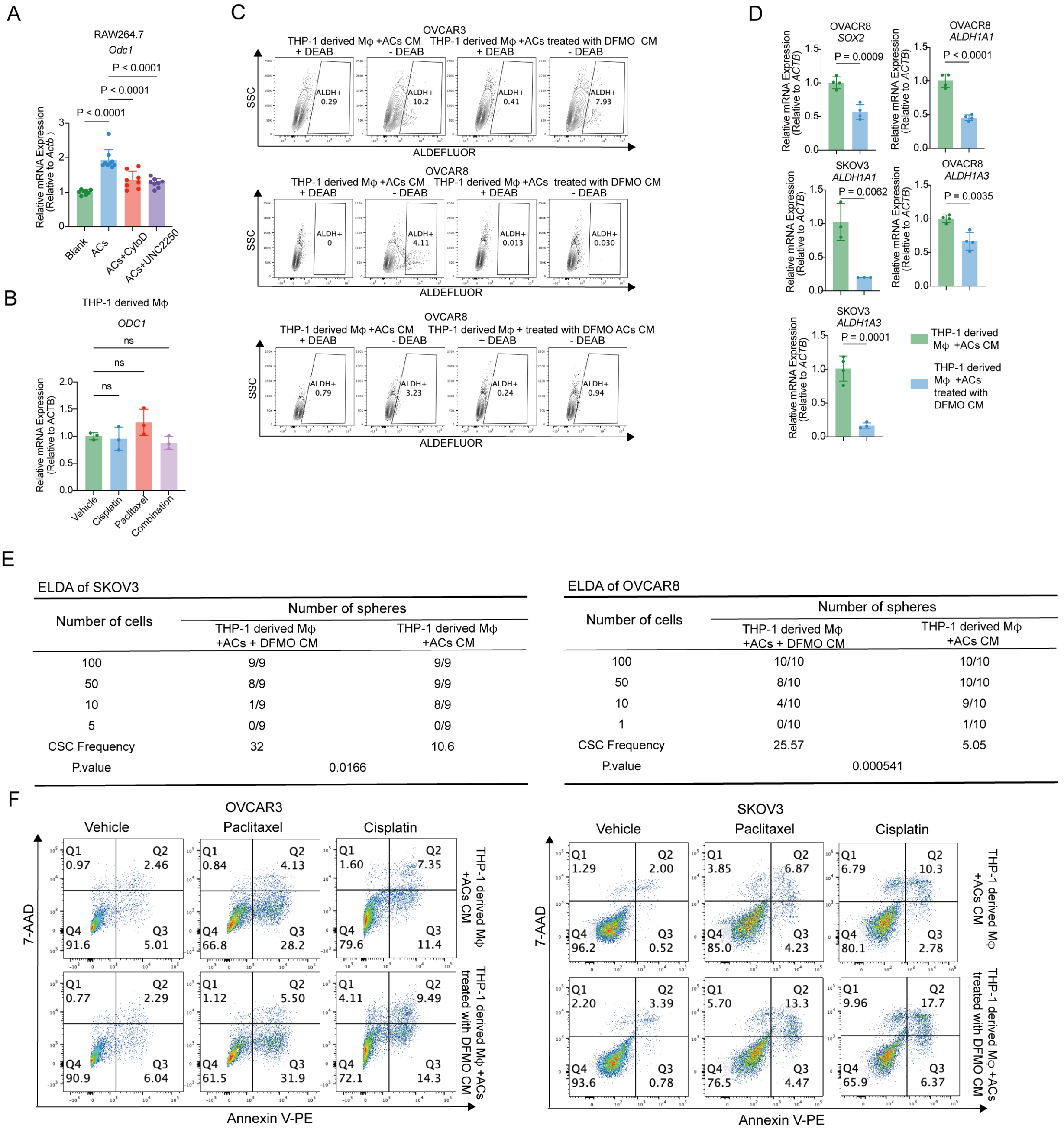

ELDA of SKOV3

Number of cells	Number of spheres: THP-1 derived Mφ +ACs + DFMO CM	THP-1 derived Mφ +ACs CM
100	9/9	9/9
50	8/9	9/9
10	1/9	8/9
5	0/9	0/9
CSC Frequency	32	10.6
P.value	0.0166	

ELDA of OVCAR8

Number of cells	Number of spheres: THP-1 derived Mφ +ACs + DFMO CM	THP-1 derived Mφ +ACs CM
100	10/10	10/10
50	8/10	10/10
10	4/10	9/10
1	0/10	1/10
CSC Frequency	25.57	5.05
P.value	0.000541	

Figure S12: DFMO inhibits cancer stemness induced by efferocytosis

A, qRT-PCR analysis of relative *Odc1* mRNA expression in Raw264.7 with different treatments (n=8). Statistical significance was tested using one-way ANOVA and Dunnett's multiple comparisons test.

B, qRT-PCR analysis of relative *ODC1* mRNA expression in THP-1 derived Mφ with different Chemo-treatments (n=3). Statistical significance was tested using one-way ANOVA and Dunnett's multiple comparisons test.

C, Representative flow cytometry images of ALDEFLUOR assays in OVCAR3 treated with THP-1 derived Mφ +ACs CM, THP-1 derived Mφ+ACs treated with DFMO CM.

D, Bar graphs showed the relative mRNA expression (relative to *ACTB*) of cancer stemness-associated genes of OC cells treated with THP-1 derived Mφ +ACs CM, THP-1 derived Mφ+ACs treated with DFMO CM separately for 48 hours. Statistical significance was tested using the Student t test.

E, Statistics tables of ELDA experiments in OVCAR8 and SKOV3 treated with THP-1 derived Mφ +ACs CM, THP-1 derived Mφ+ACs treated with DFMO CM separately.

F, Representative flow cytometry analysis images of apoptotic asssays in OVCAR3 and SKOV3 treated with THP-1 derived Mφ +ACs CM, THP-1 derived Mφ+ACs treated with DFMO CM, then received vehicle, paclitaxel, and cisplatin treatments.

All data are presented as Mean ± SD.

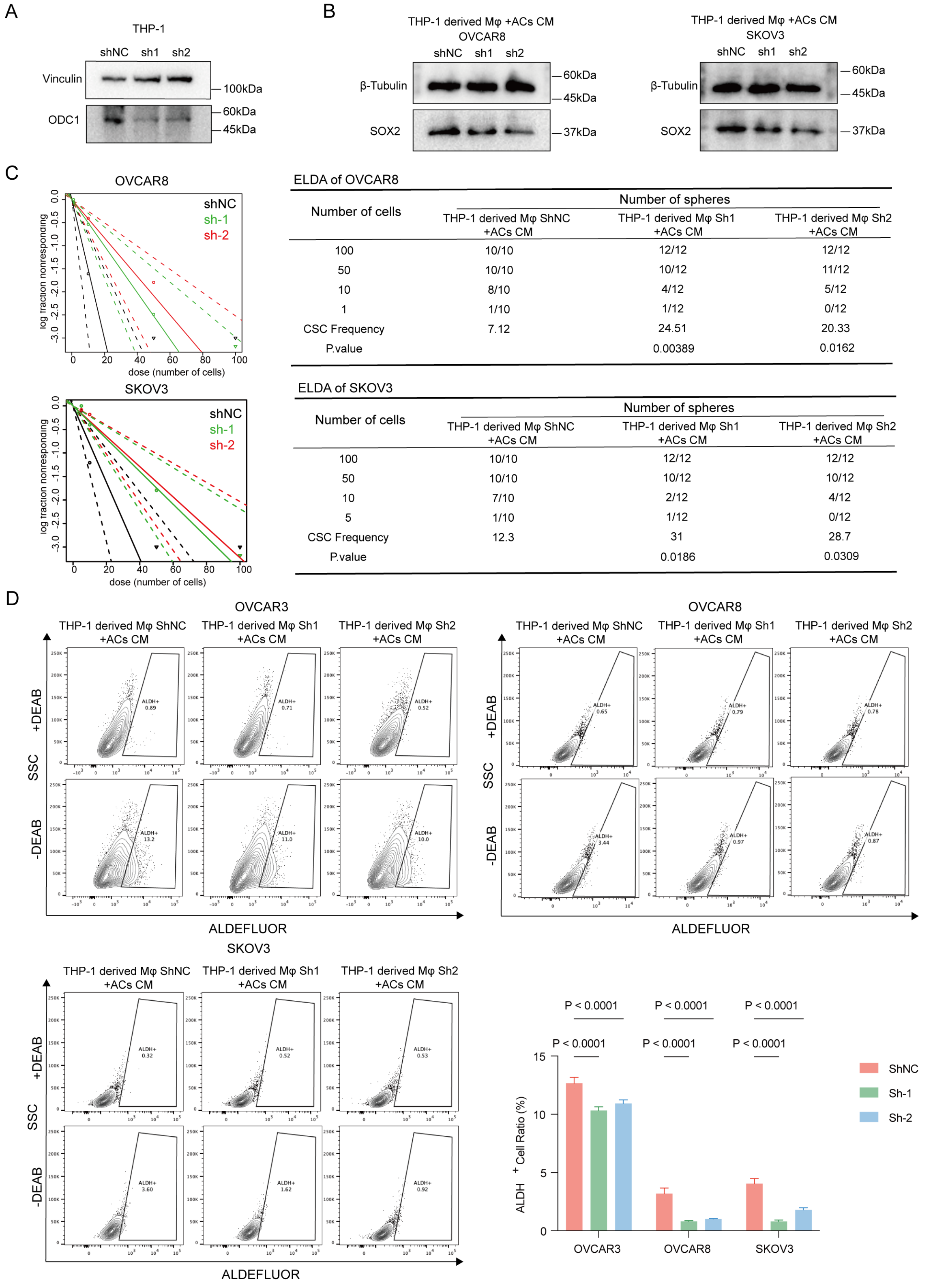

ELDA of OVCAR8

Number of cells	Number of spheres: THP-1 derived Mφ ShNC +ACs CM	THP-1 derived Mφ Sh1 +ACs CM	THP-1 derived Mφ Sh2 +ACs CM
100	10/10	12/12	12/12
50	10/10	10/12	11/12
10	8/10	4/12	5/12
1	1/10	1/12	0/12
CSC Frequency	7.12	24.51	20.33
P.value		0.00389	0.0162

ELDA of SKOV3

Number of cells	Number of spheres: THP-1 derived Mφ ShNC +ACs CM	THP-1 derived Mφ Sh1 +ACs CM	THP-1 derived Mφ Sh2 +ACs CM
100	10/10	12/12	12/12
50	10/10	10/12	10/12
10	7/10	2/12	4/12
5	1/10	1/12	0/12
CSC Frequency	12.3	31	28.7
P.value		0.0186	0.0309

Figure S13: ODC1 knockdown in macrophages inhibits cancer stemness induced by efferocytosis

A, Representative western blotting images of ODC1 levels in THP-1 derived Mφ.

B, Representative western blotting images of ODC1 levels in OVCAR8 and SKOV3 treated with THP-1-derived Mφ with ODC1 knockdown + ACs CM.

C, ELDA experiments in OVCAR8 and SKOV3 treated with THP-1-derived Mφ with ODC1 knockdown + ACs CM,.

D, ALDEFLUOR assays in ovarian cancer cells treated with THP-1 derived Mφ shNC +ACs CM, THP-1 derived Mφ sh-1 +ACs CM, and HP-1 derived Mφ sh-2 +ACs CM.

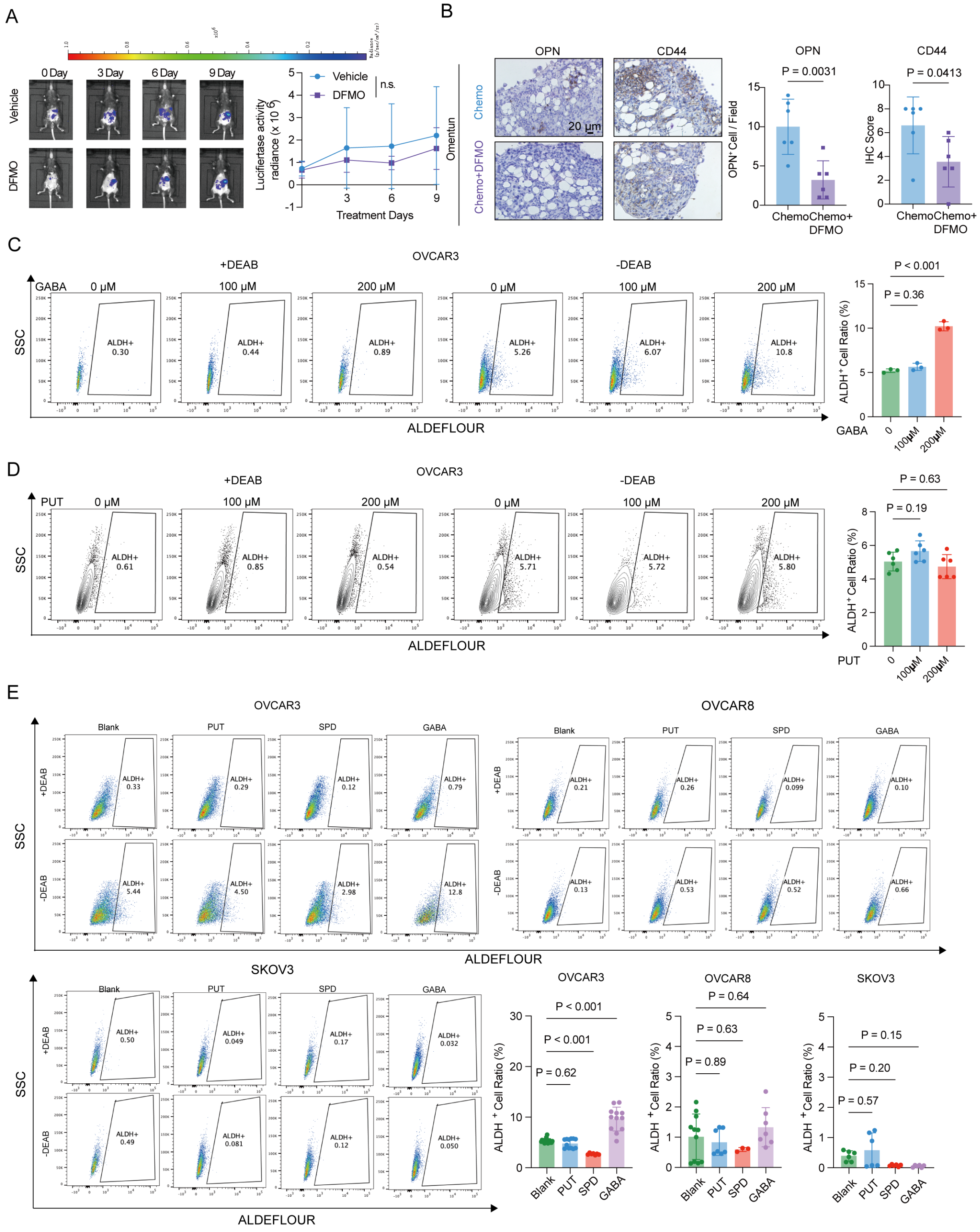

Figure S14: Polyamine associated metabolites have no significant influence on ALDH$^+$ cell ratio of OC cells.

A, Representative images of the orthotopic tumor xenografts treated with DFMO or vehicle. Tumor burden was assessed by performing in vivo imaging using a live - imaging system on a schedule of every 3 days. Tumor growth curves for mice treated with DFMO (n=4) or vehicle (n=4).

B, Representative IHC images of OPN and CD44 staining in the Omentum tumors treated with chemotherapy or chemotherapy plus DFMO. Bar graphs showing CD44, ALDH1A1 levels, and the numbers of OPN^{+} cells in the Omentum tumors. Statistical significance was tested using Student's t-test.

C, Representative flow cytometry images of ALDEFLUOR assays in OVCAR3 treated with GABA for 48 hours, and the bar graph of the ALDEFLUOR assays.

D, Representative flow cytometry images of ALDEFLUOR assays in OVCAR3 treated with putrescine for 48 hours, and the bar graph of the ALDEFLUOR assays.

E, Representative flow cytometry images of ALDEFLUOR assays in OVCAR3, OVCAR8, and SKOV3 treated with putrescine (200 μM), spermidine (50 μM), or GABA (200 μM), for 48 hours, and the bar graphs of the ALDEFLUOR assays. Statistical significance was tested using one-way ANOVA and Dunnett's multiple comparisons test.

All data are presented as Mean ± SD.

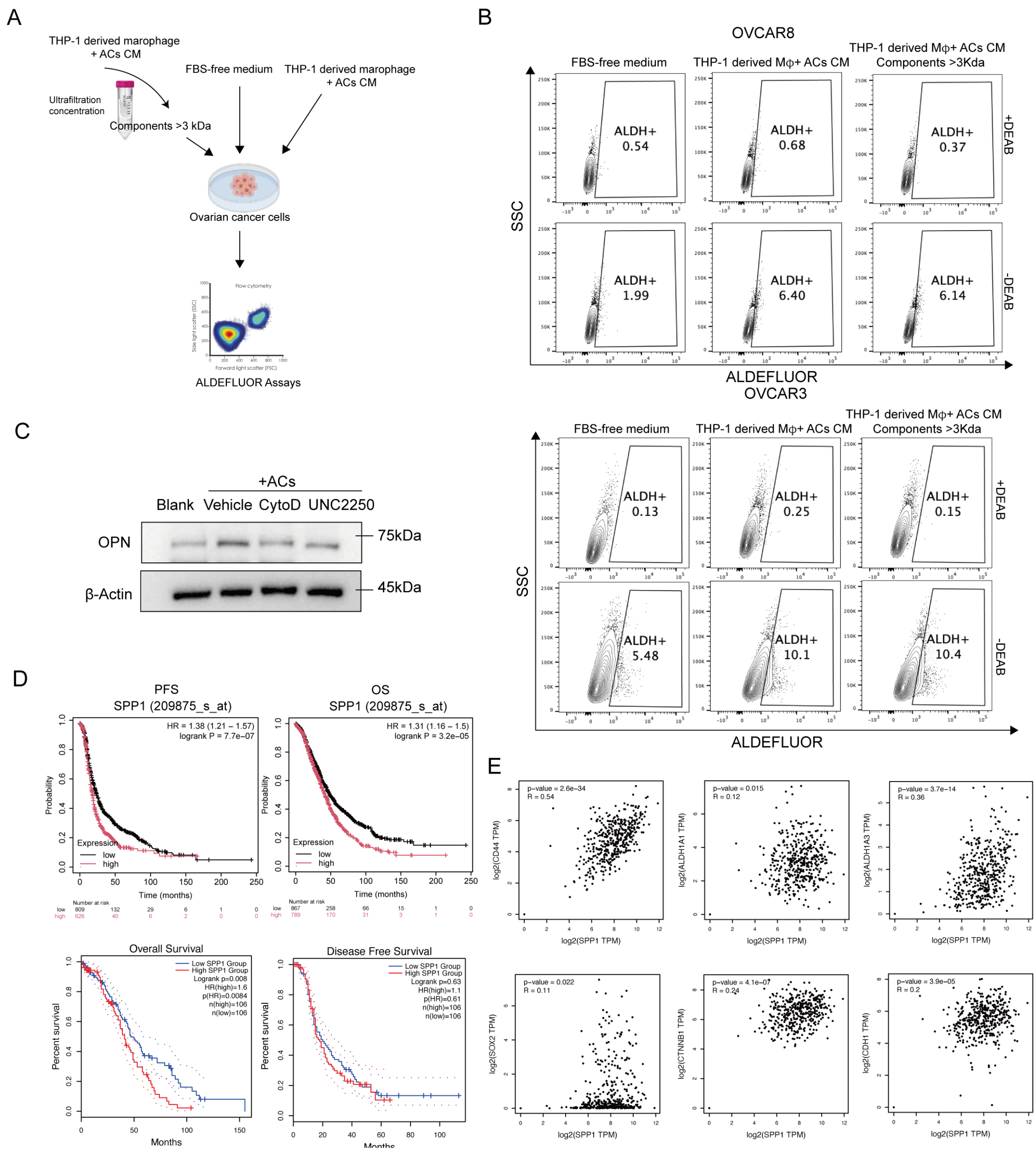

Figure S15: *SPP1* is associated with poor survival prognosis and cancer stemness in OC.

A, Flowchart of **B**.

B, Representative flow cytometry analysis of ALDEFLUOR assays in OVCAR3 and OVCAR8 treated with FBS-free medium, THP-1 derived Mφ +ACs CM, THP-1 derived Mφ+ACs CM components > 3 KDa separately for 48 hours. Statistical significance was tested using one-way ANOVA and Dunnett's multiple comparisons test. All data are presented as Mean ± SD.

C, Representative western blotting image of OPN levels in THP-1 derived Mφ with different treatments.

D, Survival analysis in ovarian cancer patients by using GEPIA2 and KM-PLOTTER.

E, Correlation of *SPP1* expression and cancer stemness-related genes. Statistical significance was tested using Spearman correlation analysis.

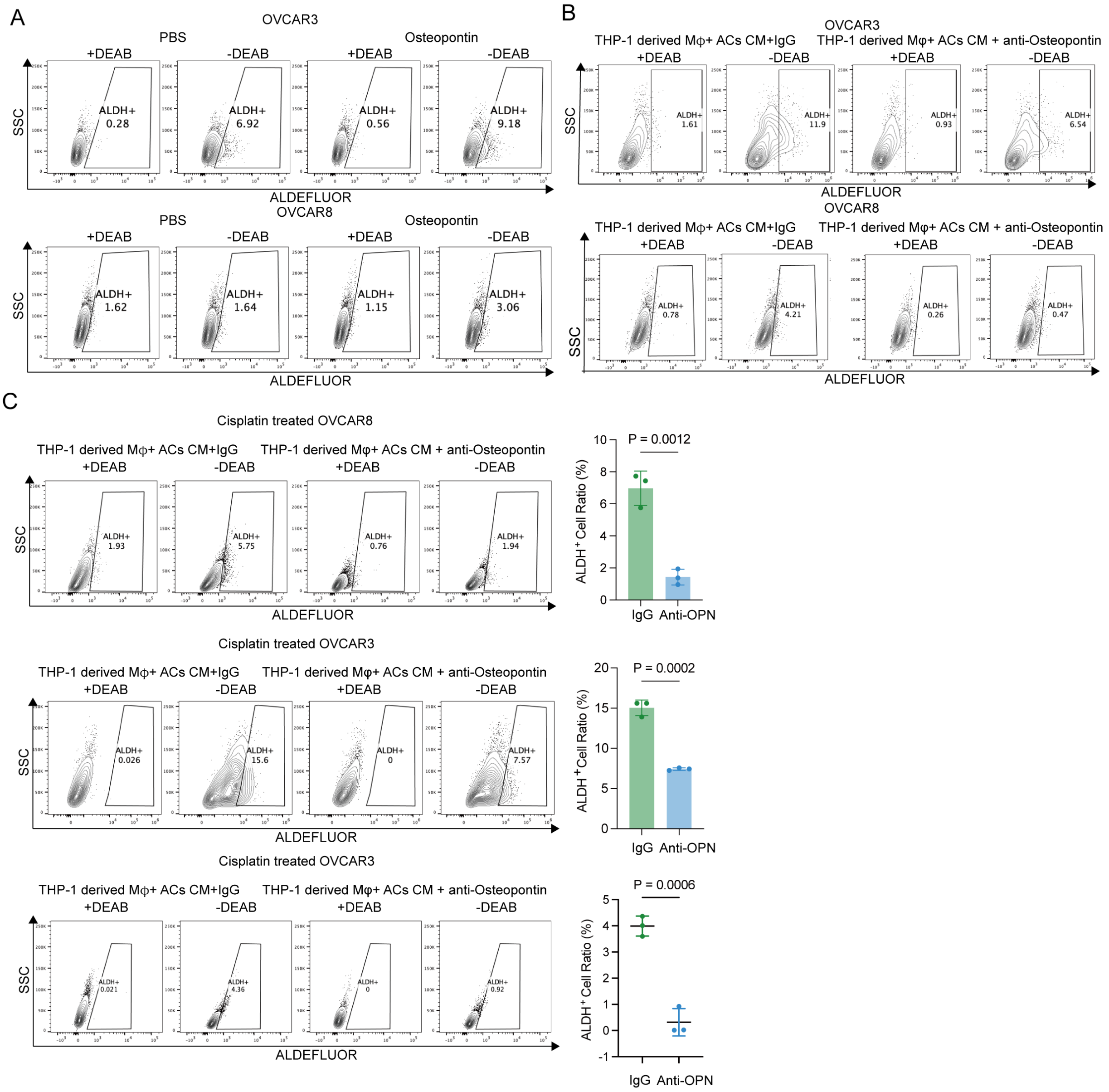

D

ELDA of SKOV3 incubated with THP-1 derived Mφ +ACs CM

Number of cells	Number of spheres	
	IgG	Anti-OPN
100	10/10	8/10
50	9/10	8/10
10	5/10	1/10
5	2/10	1/10
CSC Frequency	19.1	49.5
P.value	0.0092	

ELDA of OVCAR8 incubated with THP-1 derived Mφ +ACs CM

Number of cells	Number of spheres	
	IgG	Anti-OPN
100	10/10	10/10
50	10/10	5/10
10	8/10	3/10
1	4/10	0/10
CSC Frequency	5.04	43.03
P.value	<0.0001	

Figure S16: Efferocytotic Macrophage secretes OPN to promote cancer stemness in OC cells

A, Representative flow cytometry images of ALDEFLUOR assays in OVCAR3 and OVCAR8 treated with PBS and recombinant human OPN.

B, Representative flow cytometry images of ALDEFLUOR assays in OVCAR3, OVCAR8 treated with THP-1 derived Mφ +ACs CM + IgG and THP-1 derived Mφ +ACs CM +anti-OPN.

C, Representative flow cytometry images of ALDEFLUOR assays in cisplatin-treated OVCAR3, OVCAR8, and SKOV3 treated with THP-1 derived Mφ +ACs CM + IgG and THP-1 derived Mφ +ACs CM +anti-OPN.

D, Statistics tables of ELDA experiments in OVCAR8 and SKOV3 treated with THP-1 derived Mφ +ACs CM +IgG and THP-1 derived Mφ +ACs CM +anti-OPN

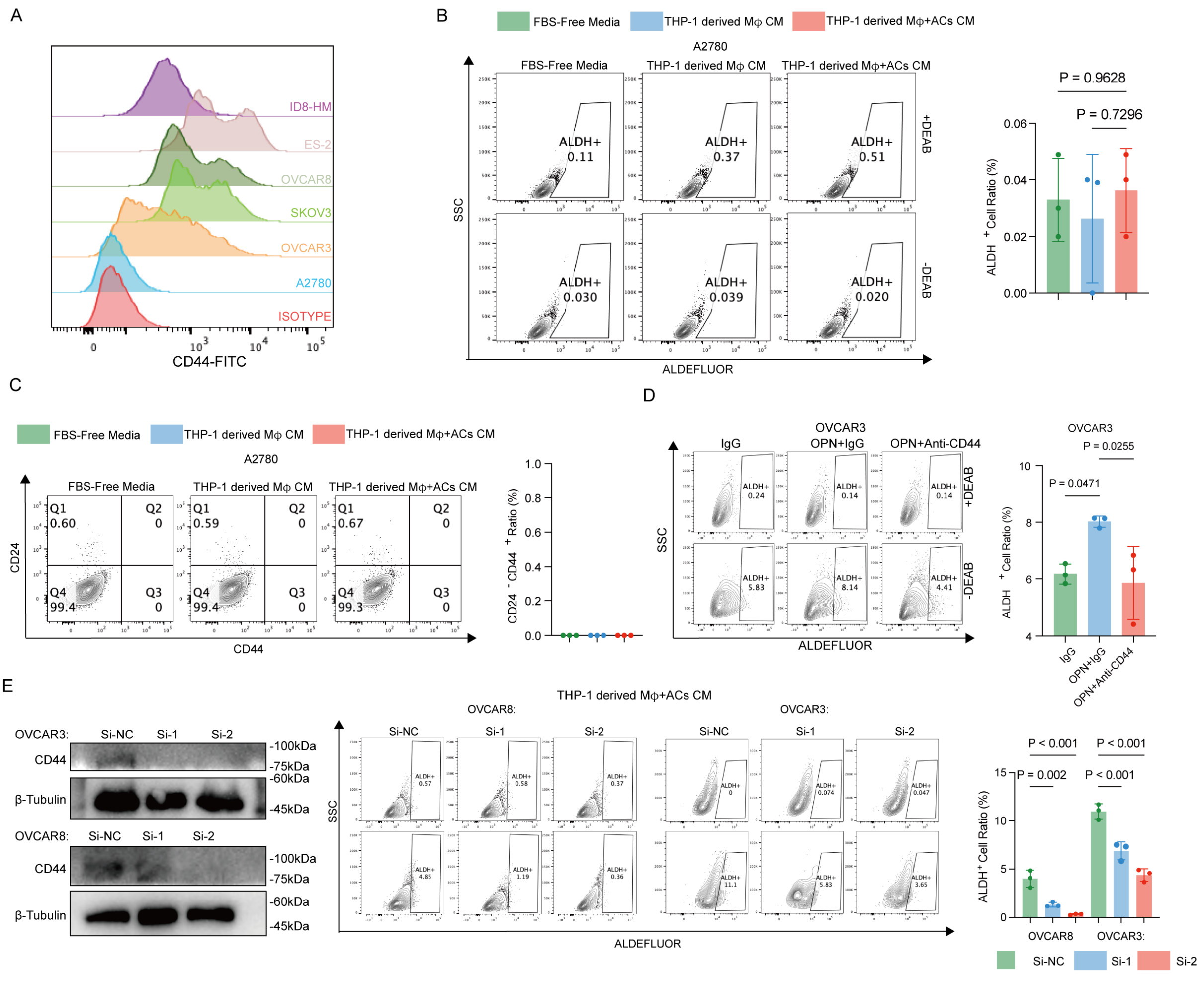

Figure S17: Efferocytosis confers cancer stemness relying on CD44 expression of OC cells

A, Representative flow cytometry images of CD44 expression in ID8-HM, ES-2, SKOV3, OVCAR3, OVCAR8, A2780.

B, Representative flow cytometry images of ALDEFLUOR assays in A2780 treated with FBS-free medium, THP-1 derived Mφ CM, THP-1 derived Mφ+ACs CM. The bar graphs showed the $ALDH^+$ cell ratio in A2780 treated with FBS-free medium, THP-1 derived Mφ CM, THP-1 derived Mφ+ACs CM. Statistical significance was tested using one-way ANOVA and Dunnett's multiple comparisons test.

C, Representative flow cytometry images of CD24 and CD44 in A2780 treated with FBS-free medium, THP-1 derived Mφ CM, THP-1 derived Mφ +ACs CM, and the bar graphs showing the result. Statistical significance was tested using one-way ANOVA and Dunnett's multiple comparisons test.

D, Representative flow cytometry analysis of ALDEFLUOR assays in OVCAR3 treated with IgG, OPN+IgG, OPN+anti-CD44. The bar graphs showing the result. Statistical significance was tested using one-way ANOVA and Dunnett's multiple comparisons test.

E, Representative western blotting images of OVCAR3 and OVCAR8 with knockdown of CD44. Representative flow cytometry analysis of ALDEFLUOR assays in OVCAR3 and OVCAR8 treated with THP-1 derived Mφ +ACs CM. The bar graphs showing the result. Statistical significance was tested using one-way ANOVA and Dunnett's multiple comparisons test.

All data are presented as Mean ± SD.

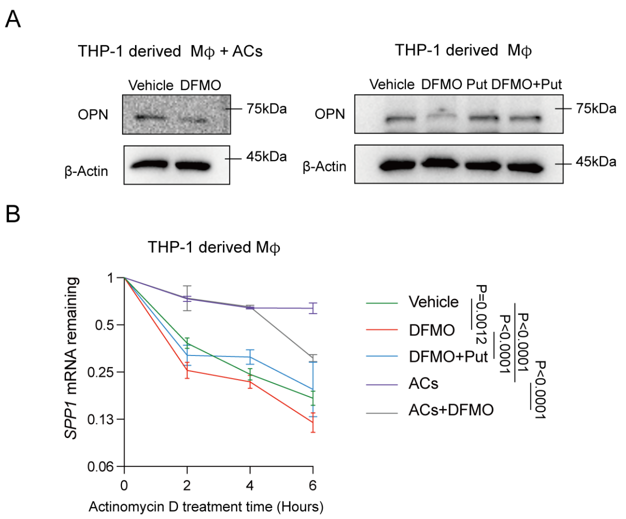

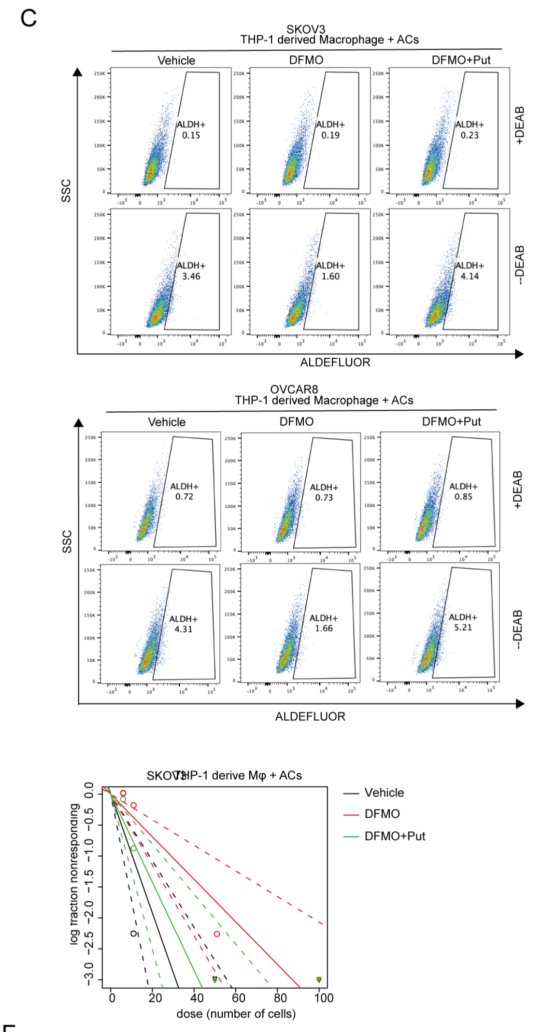

D

ELDA of OVCAR8 incubated with THP-1 derived Mφ +ACs

Number of cells	Number of spheres		
	Vehicle CM	DFMO CM	DFMO+Put CM
100	10/10	10/10	10/10
50	10/10	8/10	10/10
10	8/10	4/10	9/10
1	2/10	0/10	2/10
CSC Frequency	6.3	26.1	4.9
P.value		<0.0001	

ELDA of SKOV3 incubated with THP-1 derived Mφ +ACs

Number of cells	Number of spheres		
	Vehicle CM	DFMO CM	DFMO+Put CM
100	10/10	10/10	10/10
50	10/10	9/10	10/10
10	9/10	2/10	6/10
5	0/10	0/10	1/10
CSC Frequency	10.4	29.1	14.1
P.value		0.0346	

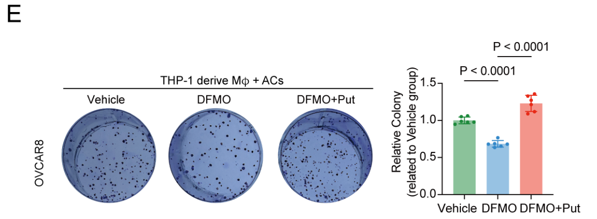

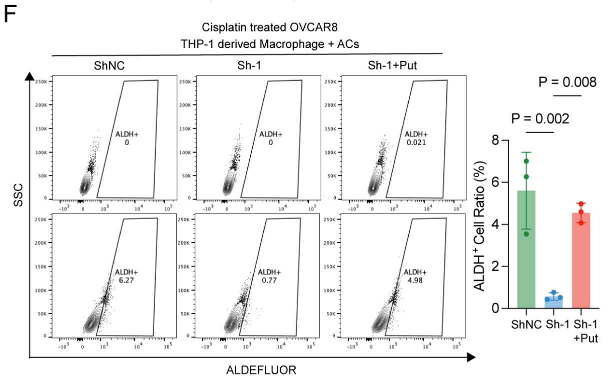

Figure S18: Inhibiting ODC1 in macrophages suppresses OPN expression and rescues CSC enrichment induced by efferocytosis.

A, Representative western blotting image of OPN levels in THP-1 derived Mφ +ACs treated with vehicle and DFMO and representative western blotting image of OPN levels in THP-1 derived Mφ treated with vehicle, DFMO, putrescine, and DFMO plus putrescine.

B, *SPP1* mRNA remaining in THP-1 derived Mφ with different treatments subjected to Actinomycin D treatments for different indicated durations as detected by qRT-PCR (n=4 independent experiments). Statistical significance was tested using two-way ANOVA and Šídák's multiple comparisons test, and performed at 6 hours.

C, Representative flow cytometry images of ALDEFLUOR assays in OVCAR8 and SKOV3 cells treated with THP-1 derived Mφ +ACs CM, THP-1 derived Mφ +ACs +DFMO CM, and THP-1 derived Mφ +ACs + DFMO +putrescine CM separately.

D, Statistics tables of ELDA experiments in OVCAR8 and SKOV3 treated with THP-1 derived Mφ +ACs CM, THP-1 derived Mφ +ACs +DFMO CM, and THP-1 derived Mφ +ACs + DFMO +putrescine CM separately.

E, Representative images of colony formation assays in OVCAR8 cells treated with THP-1 derived Mφ +ACs CM, THP-1 derived Mφ +ACs +DFMO CM, and THP-1 derived Mφ +ACs + DFMO +putrescine CM separately. Statistical significance was tested using one-way ANOVA and Dunnett's multiple comparisons test.

F. Representative flow cytometry images of ALDEFLUOR assays in cisplatin-treated OVCAR8 cells treated with THP-1 shNC derived Mφ +ACs CM, THP-1 sh-1 derived Mφ +ACs CM, and THP-1 sh-1 derived Mφ +Put +ACs CM. Statistical significance was tested using one-way ANOVA and Dunnett's multiple comparisons test.

All data are presented as Mean ± SD.

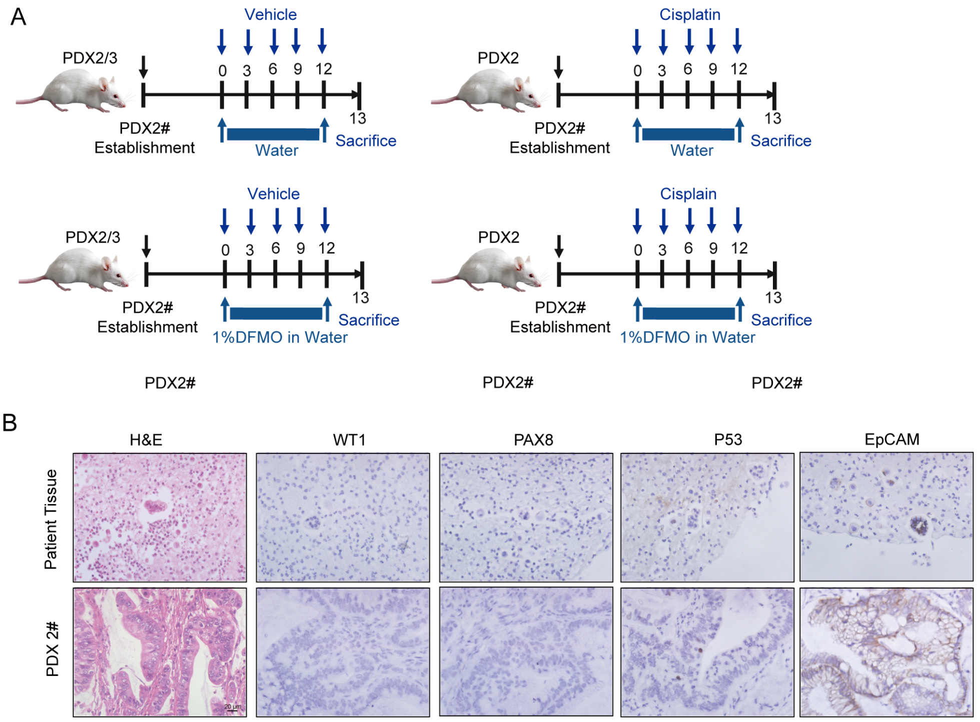

Figure S19: Inhibiting ODC1 suppress *SPP1* mRNA expression in macrophages

A, Treatment of PDX using vehicle, cisplatin, DFMO, and cisplatin combination with DFMO.

B, Identification of PDX pathological characters by IHC (Immunohistochemistry).
